# Knowledge graph–based thought: a knowledge graph–enhanced LLM framework for pan-cancer question answering

**DOI:** 10.1093/gigascience/giae082

**Published:** 2025-01-06

**Authors:** Yichun Feng, Lu Zhou, Chao Ma, Yikai Zheng, Ruikun He, Yixue Li

**Affiliations:** Hangzhou Institute for Advanced Study, University of Chinese Academy of Sciences, 310024 Hangzhou, China; Guangzhou National Laboratory, Guangzhou International Bio Island, 510005 Guangzhou, China; Guangzhou National Laboratory, Guangzhou International Bio Island, 510005 Guangzhou, China; Smartquerier Gene Technology (Shanghai) Co., Ltd., 200100 Shanghai, China; Guangzhou National Laboratory, Guangzhou International Bio Island, 510005 Guangzhou, China; BYHEALTH Institute of Nutrition & Health, 510663 Guangzhou, China; Shanghai Institute of Nutrition and Health, Chinese Academy of Sciences Shanghai, 200030 Shanghai, China; Hangzhou Institute for Advanced Study, University of Chinese Academy of Sciences, 310024 Hangzhou, China; Guangzhou National Laboratory, Guangzhou International Bio Island, 510005 Guangzhou, China

**Keywords:** pan-cancer knowledge graph, large language model, knowledge graph question answering, prompt engineering

## Abstract

**Background:**

In recent years, large language models (LLMs) have shown promise in various domains, notably in biomedical sciences. However, their real-world application is often limited by issues like erroneous outputs and hallucinatory responses.

**Results:**

We developed the knowledge graph–based thought (KGT) framework, an innovative solution that integrates LLMs with knowledge graphs (KGs) to improve their initial responses by utilizing verifiable information from KGs, thus significantly reducing factual errors in reasoning. The KGT framework demonstrates strong adaptability and performs well across various open-source LLMs. Notably, KGT can facilitate the discovery of new uses for existing drugs through potential drug–cancer associations and can assist in predicting resistance by analyzing relevant biomarkers and genetic mechanisms. To evaluate the knowledge graph question answering task within biomedicine, we utilize a pan-cancer knowledge graph to develop a pan-cancer question answering benchmark, named pan-cancer question answering.

**Conclusions:**

The KGT framework substantially improves the accuracy and utility of LLMs in the biomedical field. This study serves as a proof of concept, demonstrating its exceptional performance in biomedical question answering.

Key Points

- We introduce a framework combining large language models (LLMs) with knowledge graphs (KGs) to improve factual accuracy in LLM reasoning.
- Our system is a flexible architecture that seamlessly integrates various LLMs.
- Utilizing a pan-cancer knowledge graph, we have proposed the first knowledge graph question answering benchmark in the field of biomedicine.
- Case studies reveal our method enhanced LLMs in addressing biomedical challenges such as drug repositioning, resistance research, individualized treatment, and biomarker analysis.
- The method performs favorably in comparison to existing methods.

## Introduction

With the increasing prominence of large language models (LLMs) in the field of artificial intelligence, the advent of influential models such as ChatGPT [1] and Llama [2] consequently catalyze the development of a wide array of applications in biomedicine and health care. However, LLMs still face the challenge of factual hallucination, where they generate incorrect statements due to limited inherent knowledge [3]. Factual hallucination presents a significant challenge for the practical use of LLMs, especially in real-world scenarios where factual accuracy is crucial. Consequently, there is a growing focus on addressing factual hallucinations in LLMs within the field of natural language processing (NLP) [4, 5].

LLMs often struggle to capture and access factual knowledge, primarily due to 3 aspects: the inability to comprehend questions due to the lack of contextual information, the insufficient knowledge to generate accurate answers, and the incapacity to recall specific facts [6]. Consequently, researchers consider the fine-tuning technique as a solution to address these issues. For example, MedAlpaca [7] builds upon medical data to fine-tune Stanford Alpaca for applications related to medical question answering and dialogue. ChatDoctor [8] is designed to simulate a conversation between a doctor and a patient by fine-tuning LLaMA with medical literature. Additionally, Med-PaLM [9] shows promising performance on the MedQA exam based on clinical corpora and human feedback. Meanwhile, aiming at the Chinese medical domain, LLMs such as BenTsao [10], DoctorGLM [11], and HuatuoGPT [12] are developed on the Chinese medical dialogue data. More recently, Zhongjing [13] and ChiMed-GPT [14] adopted full pipeline training from pretraining, SFT, to reinforcement learning with human feedback (RLHF) [15]. While fine-tuning can reduce hallucinations in LLMs, it brings about considerable training expenses. Additionally, it poses a critical challenge known as catastrophic forgetting. This issue manifests when a model forgets its previously learned information as a consequence of parameter modifications during the acquisition of new tasks. This forgetfulness results in a deterioration of performance on prior tasks, consequently constraining the model’s practical applicability [16, 17].

In addition to fine-tuning, researchers also enhance the output of LLMs through the field of prompt engineering. Prompt engineering focuses on the creation and optimization of prompts to improve the effectiveness of LLMs across various applications and research domains [18]. It can enhance the capabilities of LLMs in a wide range of complex tasks, including question answering, sentiment classification, and commonsense reasoning. Chain-of-thought (CoT) prompts [19] enable complex reasoning capabilities by incorporating intermediate reasoning steps. The Automatic Prompt Engineer (APE) proposes an automatic prompt generation method aimed at enhancing the performance of LLMs [20]. Prompt engineering offers a straightforward approach to harnessing the potential of LLMs without fine-tuning.

**Figure 1:**
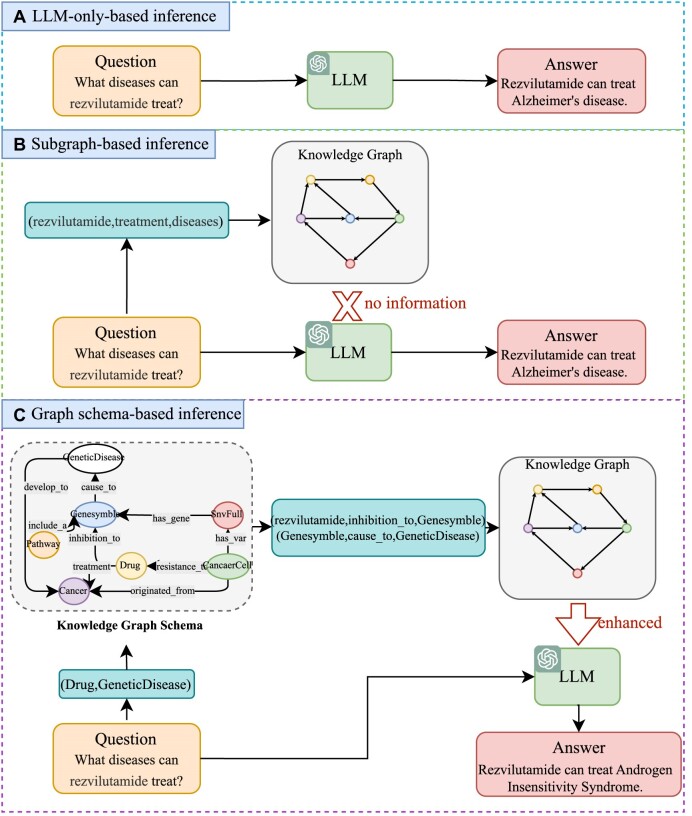
Illustrative examples contrasting our work with previous efforts. (A) LLM-only-based inference, answering questions solely through the inherent knowledge of LLMs. (B) Subgraph-based inference, enhancing LLMs by retrieving the knowledge from KGs based on the question. If intermediate entities are not provided in the multihop question, no appropriate knowledge can be retrieved. (C) Graph schema–based inference, enhancing retrieval capabilities by reasoning intermediary entity types on the schema of the KG, using the knowledge of the KG to enhance LLMs’ responses.

On the other hand, knowledge graphs (KGs) are repositories of vast quantities of high-quality structured data, offering the potential to effectively mitigate the issue of factual hallucinations when integrated with LLMs. Hence, employing KGs for question answering can enhance the precision of the responses and furnish a dependable foundation for the factual verification of information produced by LLMs. Knowledge graph question answering (KGQA) has long been a hot research topic. Before the advent of LLMs, certain studies [21–23] typically begin by retrieving a subgraph related to the question to reduce the search space, then perform multihop reasoning on this basis. This retrieval-plus-reasoning paradigm has shown its advantages over direct reasoning across the entire KG [24, 25]. Additionally, researchers tackle KGQA by parsing the question into a structured query language (e.g., SPARQL) and using a query engine to obtain accurate answers [26, 27]. UniKGQA [28] introduces a unified fine-tuning framework for retrieval and reasoning, more closely linking these 2 stages. However, traditional KGQA methods usually perform poorly in accurate semantic understanding and high-quality text generation due to the lack of LLMs for retrieval and reasoning. Hence, recent research is increasingly utilizing external KGs to enhance LLMs in addressing KGQA challenges. For instance, StructGPT [29] navigates through knowledge graphs by identifying pathways from an initial seed entity to the target answer entity, while Think-on-Graph (ToG) [30] introduces iterative exploration of the knowledge graph, which can become inefficient with very large KGs. Additionally, Reasoning on Graphs (RoG) [31] necessitates fine-tuning to accurately generate and plan the relation paths. KG-GPT [32] opts for retrieving an entire subgraph from the knowledge graph and then deduces the answer through inference. Although these methods have achieved gratifying results in general areas, as shown in Fig. 1B, when the intermediate entity in the multihop question is unknown, it is impossible to retrieve the appropriate knowledge from the KG.

In this article, we introduce an innovative framework called knowledge graph–based thought (KGT), which integrates LLMs with KGs through employing LLMs for reasoning on the schema of KGs to mitigate factual hallucinations of LLMs, as shown in Fig. 1C. Unlike traditional methods, KGT does not directly retrieve factual information based on the question. Instead, it uses LLMs to infer entity information on the schema of the knowledge graph, generating an optimal subgraph based on key information directly extracted from the question and inferred information from the schema. Subsequently, the optimal subgraph is used to infer the answer to the question through LLMs. KGT requires no fine-tuning, offers seamless integration with multiple LLMs, and is plug-and-play, facilitating easy deployment. It demonstrates generalizability, making it adaptable for use with diverse knowledge graphs. This framework is tailored for wide-ranging applications in numerous biomedical challenges, such as (i) enhancing clinical decision-making for physicians and medical organizations, (ii) delivering medical advice to patients and health care providers, (iii) uncovering crucial biomarkers for early disease detection and tailored therapy, and (iv) exploring novel therapeutic applications for existing medications through insights into their mechanisms, side effects, and the biological processes of associated diseases. Furthermore, we utilize the SmartQuerier Oncology Knowledge Graph (SOKG), a pan-cancer knowledge graph developed by SmartQuerier, to create a benchmark for the KGQA task within biomedicine, named pan-cancer question answering (PcQA). We release this benchmark and its accompanying knowledge graph, which is a subgraph of the SOKG, in [33]. This benchmark is currently the sole question-answering dataset available in the domain of biomedical knowledge graphs.

## Materials and Methods

### Knowledge graph introduction

In this work, we tackle the problem of logical reasoning over the KG $\mathcal {K}: {\it E} \times {\it R}$ that store entities (*E*) and relations (*R*). Without loss of generality, KG can be organized as a set of triplets $\lbrace ({\it e}_1, {\it r}, {\it e}_2)\rbrace \subseteq \mathcal {K}$, where each relation ${\it r} \in {\it R}$ exists between the pair of entities $(e_1, e_2) \in {\it E} \times {\it E}$. We define a relational path $\lbrace ({\it t}_1, {\it r}, {\it t}_2)\rbrace$ as a sequence of entity types (*T*) and the relation between them, where $(t_1, t_2) \in {\it T} \times {\it T}$. In contrast, a relational chain $\lbrace ({\it e}_1, {\it r}, {\it e}_2)\rbrace$ refers to a specific set of relational triplets between entities. To further enrich the KG, attribute information is included through pairs $({\it e}, {\it attr})$, where ${\it attr}$ represents an attribute associated with an entity ${\it e}$, thereby enhancing the KG’s semantic richness and precision by incorporating detailed characteristics of each entity.

Within the specialized realm of pan-cancer research, we use a subgraph of the SOKG that provides detailed oncological information. As depicted in Table 1, SOKG includes a collection of over 3 million entities, which is substantially larger than the entity count in the compared knowledge graphs, SynLethKG [34] and SDKG [35], with 540,012 and 165,062 entities, respectively. Furthermore, SOKG’s nearly 6 million unique concept relations exceed those of SynLethKG and SDKG, which have 2,231,921 and 727,318 relations, respectively. Additionally, SOKG includes 98 distinct attribute types, enriching data comprehension and improving the efficiency and precision of queries, a capability not matched by SynLethKG or SDKG, which do not include comparable attributes. For this research, we utilize only a subgraph of the SOKG, which is available as open data [33], while the full knowledge graph remains proprietary.

**Table 1.** Comparison of SOKG with SynLethKG and SDKG

	Entity types	Relational types	Nodes	Edges	Attributes
SynLethKG	11	24	54,012	2,231,921	0
SDKG	7	12	165,062	727,318	0
**SOKG**	**24**	**21**	**3,640,259**	**10,656,273**	**98**

### Task description

In order to tackle a diverse array of challenges in the field of biomedicine, we have designed 4 categories of problems: 1-hop problems, multihop problems, intersection problems, and attribute problems, as illustrated in Table 2. Based on these 4 types of tasks, we leverage the SOKG to establish a benchmark for the KGQA task within biomedicine, named PcQA. Unlike KGQA tasks in general domains, such as MetaQA [36] and FACTKG [37], which typically provide the entity types of intermediate entities, KGQA problems in the biomedical domain often do not have any information about intermediate entities. Instead, the information about intermediate entities must be inferred from the question itself rather than being directly provided, as shown in Supplementary Table S1. Additionally, our PcQA dataset includes attributes such as whether a drug is targeted therapy or if a mutated gene is oncogenic. This makes our tasks slightly more challenging and better suited to the actual needs of biomedical KGQA.

**Table 2.** Four different reasoning types of task. Each reasoning type may include overlapping questions, so the sum across the 4 different reasoning types of the task may exceed the total number of questions

Reasoning type	Claim example	Graph	Question number
One-hop	What types of cancer can be treated with diethylstilbestrol?	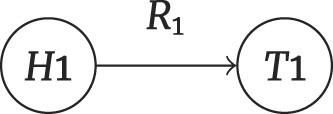	243
Multihop	What genetic mutations are present in adenoid cystic carcinoma?	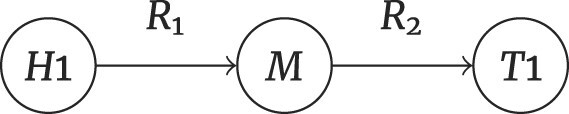	124
Intersection	Which drugs are ALK in basaloid large cell carcinoma of the lung sensitivity to?	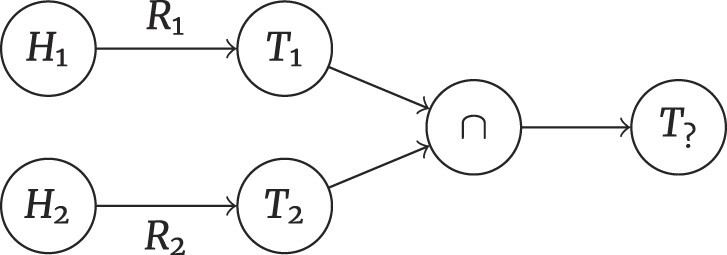	37
Attribute	What is the maximum age for recruitment of clinical trials for patients with meningioma?	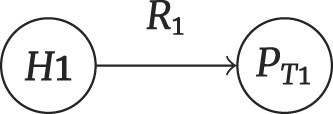	59

#### One-hop problems

One-hop problems involve single-relation chain reasoning, where the objective is to deduce the tail entity $ {\it T}_{?}$ given a head entity $ {\it H}_{1}$ and a relation $ {\it R}_{1}$, or to infer the relation $ {\it R}_{?}$ when a head entity $ {\it H}_{1}$ and a tail entity $ {\it T}_{1}$ are known, as depicted in equations (1) and (2).


(1) \begin{eqnarray*}
H_{1} + R_{1} \rightarrow T_{?}
\end{eqnarray*}

(2) \begin{eqnarray*}
H_{1} + T_{1} \rightarrow R_{?}
\end{eqnarray*}

#### Multihop problems

Multihop problems involve multiple-relation chain reasoning that can be broadly categorized into 2 types. The first category involves deducing potential relationships between entities by navigating through indirect relations. By examining the indirect relations ($ {\it R}_{1} , {\it R}_{2}$) between a head entity $ {\it H}_{1}$ and a tail entity $ {\it T}_{1}$, it is possible to infer an unknown or potential relation $ {\it R}_{?}$ linking them directly. This inference process is encapsulated in the following equation:


(3) \begin{eqnarray*}
H_{1} + T_{1} \rightarrow R_{1} + R_{2} \rightarrow R_{?}
\end{eqnarray*}

The second category extends the reasoning to include the discovery of entities themselves by following a path from a head entity through intermediate relations to a final tail entity. Starting with a head entity $ {\it H}_{1}$, coupled with an indirect relation $ {\it R}_{1}$, an intermediary entity $ {\it M}$ can be inferred. This intermediary entity $ {\it M}$ is then applied with an indirect relation $ {\it R}_{2}$ to deduce the final tail entity $ {\it T}_{?}$. This inference process is summarized in the following equation:


(4) \begin{eqnarray*}
H_{1} + R_{1} \rightarrow M + R_{2} \rightarrow T_{?}
\end{eqnarray*}

#### Intersection problems

Intersection problems refer to taking the intersection of multiple relational chains. Two head entities ($ {\it H}_{1} , {\it H}_{2}$) lead to the deduction of 2 types of tail entities ($ {\it T}_{1} , {\it T}_{2}$) based on different relations ($ {\it R}_{1} , {\it R}_{2}$). The final tail entity $ {\it T}_{?}$ is determined by intersecting these 2 types of tail entities ($ {\it T}_{1} , {\it T}_{2}$). This inference process is summarized as following:


(5) \begin{eqnarray*}
H_{1} + R_{1} \rightarrow T_{1}
\end{eqnarray*}

(6) \begin{eqnarray*}
H_{2} + R_{2} \rightarrow T_{2}
\end{eqnarray*}

(7) \begin{eqnarray*}
T_{1} \cap T_{2} \rightarrow T_{?}
\end{eqnarray*}

#### Attribute problems

Attribute problems refer to the attribute information of the entity, where the task involves retrieving the attributes of a known head entity $ {\it H}_{1}$ or determining whether the tail entity $ {\it T}_{1}$, identified through a known head entity $ {\it H}_{1}$ and relation $ {\it R}_{1}$, satisfies the attributes specified in the query, as illustrated in equations (8) and (9).


(8) \begin{eqnarray*}
H_{1} \rightarrow P_{H_{1}}
\end{eqnarray*}

(9) \begin{eqnarray*}
H_{1} + R_{1} \rightarrow P_{T_{1}}
\end{eqnarray*}

### Datasets

In the continuously evolving field of biomedical research, the integration of LLMs with KGs offers a more efficient and effective method for knowledge discovery and utilization, particularly in advancing cancer research. Nonetheless, we note a scarcity of appropriate datasets for evaluating these sophisticated methodologies within this field. To address this, we leverage the SOKG to establish a benchmark for the KGQA task within biomedicine, named PcQA. Our questions were carefully crafted by experts based on the content of the knowledge graph. GPT-4 [38] was then employed to generate Cypher queries, which were used to retrieve answers from the knowledge graph. The generated Cypher queries and corresponding answers underwent an initial review by a biomedical PhD candidate, who manually verified and corrected the dataset against the knowledge graph. Finally, the entire dataset was thoroughly reviewed by 2 biomedical experts to ensure its accuracy and reliability. This multistep process was meticulously designed to uphold the highest standards of quality throughout the dataset creation. This dataset, along with the accompanying knowledge graph, is completely open source [33]. The PcQA includes 405 data entries, covering a wide range of applications in the field of pan-cancer research, including genetic predisposition to cancer, medication treatment planning, drug repositioning, identification of potential drug targets, studies on drug resistance, and predictions of cancer progression and metastasis. By deeply exploring cancer-related reasoning and information retrieval challenges, this dataset can inspire researchers and clinicians to gain a deeper understanding of cancer and explore more effective treatment methods.

### KGT framework

**Figure 2:**
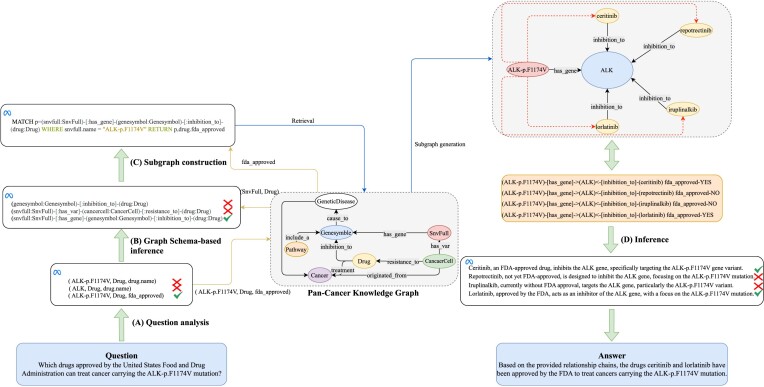
Framework of KGT. (A) Question analysis. Decompose the question and extract its key information. (B) Graph schema–based inference. Input the types of the head and tail entities into the graph schema of the knowledge graph, complete the graph reasoning, and obtain the optimal relational path. (C) Subgraph construction. Generate a query statement and retrieve the subgraph. (D) Inference. Complete the final reasoning and output the results in natural language. Note: The symbol “$\times$” represents content that has been filtered out by the LLM, while “$\checkmark$” denotes the optimal content selected by the LLM.

The overall framework of KGT is laid out in Fig. 2. When users input their question in natural language, the first step is to analyze the question, extracting the main information with the goal of breaking down the question into smaller, more manageable units. This main information is then passed to an LLM, which applies graph reasoning on the schema graph of the knowledge graph, yielding the optimal relational path. Subsequently, a retrieval statement is generated, and a subgraph is constructed within the KG through search. The relational chains and attributes in the subgraph are then fed back into the LLM to finalize the reasoning and generate an output in natural language.

#### Question analysis

##### Key information extraction

The user inputs a question text (*Q*) in natural language, which is initially deconstructed and parsed. An LLM is applied to analyze the question, resulting in the identification of the head entity name (${\it H}_{n}$), the tail entity type (${\it T}_{t}$), and the attributes of tail entity (${\it T}_{a}$). The prompt for the LLM to extract key information from the question is presented in Supplementary Fig. S1.

##### Retrieving key information from the KG

Based on ${\it H}_{n}$, a fixed Cypher format is set to query the head entity type (${\it H}_{t}$), facilitating subsequent reasoning.

#### Graph schema–based inference

##### Construction of a graph based on KG schema

Based on the entity types (${\it E}_t$) and the relations (*R*) between them in the SOKG, an undirected graph $\mathcal {G}$ is established where ${\it E}_t$ serve as nodes $\mathcal {N}$ and *R* act as edges $\mathcal {P}$.

##### Candidate path search

Breadth-first search (BFS) is employed to identify the shortest paths connecting ${\it H}_{t}$ and ${\it T}_{t}$ from the constructed graph $\mathcal {G}$. Initiate the search at ${\it H}_{t}$, creating a queue to hold nodes encountered along the way. Simultaneously, form a set to track nodes that have been visited to avoid revisiting them. Insert ${\it H}_{t}$ into the queue. Continue processing as long as the queue remains nonempty, removing a node from the queue at each step. For each of its unvisited neighbors, enqueue the neighbor, mark it as visited, and log the pathway from ${\it H}_{t}$ to this neighbor. Upon arrival at ${\it T}_{t}$, use the accumulated path data to compile the set of shortest paths (*SPs*) from ${\it H}_{t}$ to ${\it T}_{t}$, with each individual path within the set referred to as an *SP*. The nodes in each *SP* represent entity types, while the edges denote the relationships between these entity types.

##### Optimal path selection

By utilizing embedding technology, textual information is mapped into a low-dimensional space, resulting in N-dimensional real-value vectors. The similarity between each *SP* and the *Q* is calculated based on their respective real-value vectors, with the *SP* exhibiting the highest similarity being selected as the optimal path (*OP*).


(10) \begin{eqnarray*}
\text{Similarity} (Q, SP) &=& \frac{Q \cdot SP}{\Vert Q\Vert \times \Vert SP\Vert } \\
&=& \frac{\sum _{i=1}^{n} (Q_i \times SP_i)}{\sqrt{\sum _{i=1}^{n} Q_i^2} \times \sqrt{\sum _{i=1}^{n} SP_i^2}}
\end{eqnarray*}

(11) \begin{eqnarray*}
\text{OP}={\underset{Q,SP}{\rm max}}\text{ Similarity} (Q, SP)
\end{eqnarray*}

#### Subgraph construction

##### Query statement generation

Input ${\it H}_{t}$, ${\it H}_{n}$, ${\it T}_{t}$, ${\it T}_{a}$, and *OP* into an LLM to generate a query statement, such as Cypher. Text2Cypher Prompt is presented in Supplementary Fig. S2.

##### Subgraph generation

Enter the query statement in the KG to obtain a reasonable subgraph.

#### Inference

##### Subgraph inference

Based on the relational chains and attribute data in the subgraph, determine the relevance to the question text. Prune any erroneous information, retaining only the correct relational chains.

##### Natural language output

The LLM divides the subgraph into multiple relational chains, each of which outputs a sentence in natural language, and then the LLM generates natural language output. LLMs Inference and Output Prompt is presented in Supplementary Fig. S3.

## Results

### Evaluation criteria

We use evaluators based on GPT-4 [38], BERTScore [39], and ROUGE [40] to assess the accuracy of the generated answers. As a scoring bot, GPT-4 evaluates and assigns scores based on the similarity in meaning between 2 sentences. GPT-4–based Evaluation Prompt is presented in Supplementary Fig. S4. BERTScore evaluates semantic similarity using context-sensitive embeddings, offering a comprehensive evaluation of language model outputs. ROUGE, on the other hand, evaluates the longest common subsequence (LCS) between the generated text and the reference text, focusing on sequence-based similarity to assess the fluency and the preservation of semantic content.

### Baselines

To assess the advantages of our framework, we compare it with several approaches that can be directly applied for KGQA tasks without fine-tuning. We introduce a straightforward baseline approach, named Base, which is similar to KG-GPT [32], currently the leading method in the KGQA field, excluding the sentence segmentation step of KG-GPT. Initially, this involves leveraging an LLM to retrieve relevant information from the KG by generating a query statement. Then, another LLM is used to answer the question with the retrieved information. To enhance the baseline, we incorporate CoT prompting [19] and in-context learning (ICL) techniques [41], collectively referred to as CoT&ICL. The prompts for these methods are illustrated in Supplementary Table S5. Additionally, we implement KG-GPT [32] to enhance the retrieval and reasoning capabilities of the LLMs. For a fair comparison, all methods are based on Code-Llama-13B [42].

To further underscore the efficacy of our framework, we conduct a comparative analysis of KGT, which is built upon Code-Llama-13B, against 2 highly capable large language models that are prominent in the general and biomedical domains: ChatGPT-3.5 [1] and Taiyi [43]. ChatGPT-3.5, a leader in tasks across the general domain, has exhibited competitive performance in a wide range of applications. To compensate for its limited biomedical knowledge, we employ 2 methodologies previously described, Base and CoT&ICL, as advanced baselines to augment ChatGPT-3.5’s capabilities. Taiyi, a cutting-edge LLM in biomedicine, pretrained on 2 trillion tokens, leverages its extensive biomedical knowledge base for direct question answering, bypassing the need for knowledge graph retrieval.

Due to the scarcity of KGQA datasets within the biomedical domain, all experiments are conducted on our newly proposed benchmark, named PcQA.

### Comparative analysis across different KGQA methods

We evaluated the capabilities of various methods based on Code-Llama-13B, with the experimental results presented in Table 3. The experimental results indicate that the Code-Llama-13B model, enhanced with KGT, consistently surpasses competing methods across all metrics assessed. Notably, KG-GPT improves the F1 score by 15.7% over previous methods CoT&ICL, while our method KGT increases the F1 score by 33% over KG-GPT. Because KG-GPT overlooks the impact of entity types and attributes on answers within the biomedical domain, this achievement positions our approach as a pioneering benchmark in biomedical KGQA, eclipsing previously established best practices.

**Table 3.** Comparison of results between KGT and other commonly used methods based on the Code-Llama-13B. The best results are displayed in bold for each indicator

			ROUGE (%)		
Method	GPT-4 Eval (%)	BERTScore (%)	Recall	Precision	F1 score
Base	46.6	85.3	25.3	28.5	24.5
CoT&ICL	57.9	88.8	38.9	39.4	37.6
KG-GPT	68.2	93.5	55.2	55.8	53.3
**KGT (ours)**	**92.4**	**97.7**	**87.4**	**87.7**	**86.8**

### Comparative analysis across diverse LLMs

We present a comparative study of KGT applied to Code-Llama-13B against 2 highly capable LLMs in the general and biomedical domains, with experimental results displayed in Table 4. Code-Llama-13B, enhanced by KGT, significantly outperforms its peers, achieving the highest marks in every assessment metric: a GPT-4 Eval score of 92.4, a BERTScore of 97.7, and a ROUGE F1 score of 86.8. Remarkably, our approach’s F1 score surpasses that of ChatGPT-3.5 with the Base method by 52.7%, the CoT&ICL method by 36.3%, and Taiyi’s base model by 67.3%. These results highlight KGT’s substantial contribution to improving the performance of large language models for the pan-cancer KGQA task. Even when integrated with open-source general models, KGT exhibits remarkable performance, outstripping both the recognized state-of-the-art closed-source large language models and those specifically tailored for the biomedical domain. This showcases KGT’s adeptness at parsing and leveraging knowledge graph data, setting a new standard for future research and applications in the field.

**Table 4.** Comparison of KGT based on Code-Llama-13B with results from other commonly used models. The best results are displayed in bold for each indicator

				ROUGE (%)		
Model	Method	GPT-4 Eval (%)	BERTScore (%)	Recall	Precision	F1 score
ChatGPT-3.5	Base	65.4	91.0	42.7	32.3	34.1
	CoT&ICL	70.3	93.3	57.0	50.6	50.5
Taiyi	\	40.6	85.3	15.4	39.6	19.5
**Code-Llama-13B**	**KGT (ours)**	**92.4**	**97.7**	**87.4**	**87.7**	**86.8**

### Assessing KGT’s effectiveness on diverse LLM platforms

**Figure 3:**
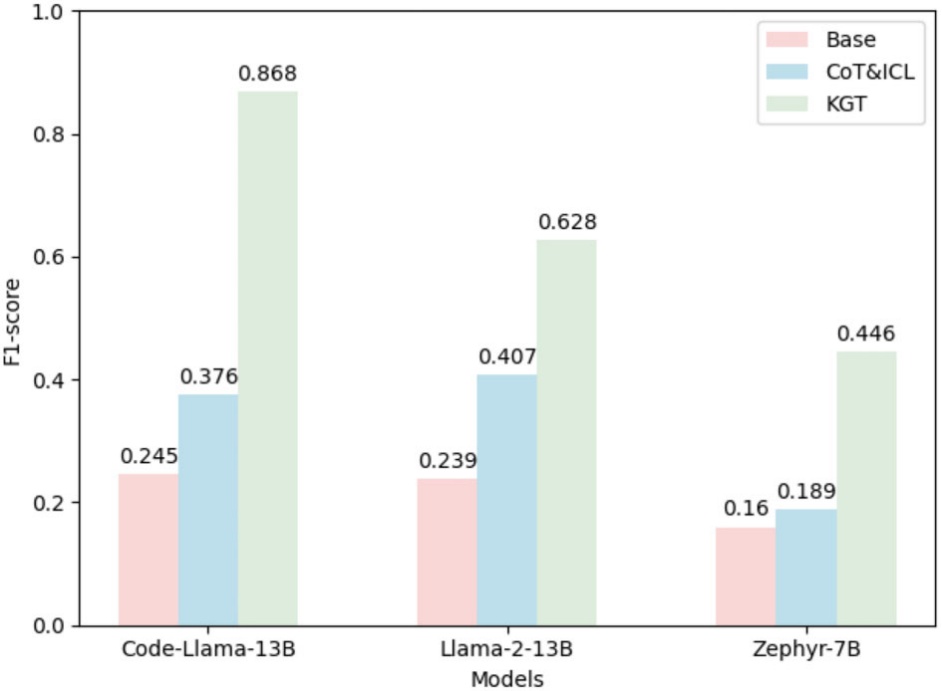
Performance of various models using different strategies.

To underscore the adaptability and effectiveness of our KGT framework when applied to a range of large language models, we conduct experiments on several LLMs: Zephyr [44], Llama-2 [2], and Code-Llama [42]. The outcomes, illustrated in Fig. 3, reveal that while the CoT&ICL techniques significantly boost performance in terms of F1 score, our KGT methodology delivers even more substantial enhancements across all evaluated models. This demonstrates not only the effectiveness of CoT&ICL as a performance-enhancing strategy but also highlights the superior advancements and impact of KGT, establishing its dominance and efficiency in knowledge graph question-answering tasks.

### Ablation study for dissecting the components of KGT

In our effort to illuminate the individual contributions of the components that constitute our KGT framework and their collective impact on enhancing the performance of LLMs, we define 4 foundational modules: (i) question analysis for the extraction of pivotal information, (ii) graph schema–based inference to identify the optimal relational chains in the knowledge graph, (iii) the generation of query statements to facilitate subgraph construction, and (iv) the inference process coupled with the articulation of results in natural language. This ablation study, grounded on the Code-Llama-13B model, is meticulously designed to evaluate the efficacy of these components. Since graph schema–based inference requires the process of question analysis, the question analysis module cannot be removed in isolation; simultaneously, subgraph construction is indispensable for knowledge graph retrieval. If the subgraph construction module is independently omitted, the outputs of the initial 2 modules will not impact the final results, making the isolated exclusion of this component illogical. Therefore, we introduce 3 specific ablated configurations for examination: (i) excluding graph schema–based inference (without GSBI), (ii) omitting both question analysis and graph schema–based inference (without QA&GSBI), and (iii) removing question analysis, graph schema–based inference, and subgraph construction (without QA&GSBI&SC), effectively bypassing the structured query of the SOKG and relying solely on the LLM’s inherent knowledge for question answering.

The results of the ablation study, as shown in Table 5, demonstrate that when we remove the GSBI, we observe a 20% decrease in the F1 score. Removing both GSBI and QA results in an additional 8.6% decrease in the F1 score compared to removing GSBI alone. Furthermore, removing GSBI, QA, and SC together leads to a 46% decrease in the F1 score compared to removing just GSBI and QA. The experiments reveal that SC is crucial; its absence forces the LLM to rely solely on its inherent knowledge, significantly reducing effectiveness. GSBI is also key, as it aids in navigating complex multihop questions by providing necessary intermediate entity information for subgraph construction. QA is equally essential, ensuring accurate identification of entities and properties for correct subgraph construction. All these variants underperform compared to the complete KGT, indicating that each of the 3 modules is vital for the final performance. Furthermore, such observations confirm that our KGT can indeed leverage knowledge to enhance the final performance of LLMs.

**Table 5.** Ablation study of the KGT framework under Code-Llama-13B

			ROUGE (%)		
Method	GPT-4 Eval (%)	BERTScore (%)	Recall	Precision	F1 score
KGT (ours)	92.4	97.7	87.4	87.7	86.8
Without GSBI	71.8	95.5	68.1	69.8	66.8
Without QA&GSBI	69.7	94.7	55.0	66.3	58.2
Without QA&GSBI&SC	24.7	77.4	14.8	12.3	12.2

### Implementation settings

Our knowledge graph is quite large, with a complex schema, and typically involves input tokens within 1,300. Our experiment does not require fine-tuning, and the inference time is related to the model size and computational resources. For example, when using our method, KGT, with the Code-Llama-13B model on an 80 GB A100 GPU, it occupies 33 GB of VRAM. Without any acceleration frameworks, the inference requires 4 passes, each taking around 20 seconds.

### Case studies

#### Drug repositioning

**Figure 4:**
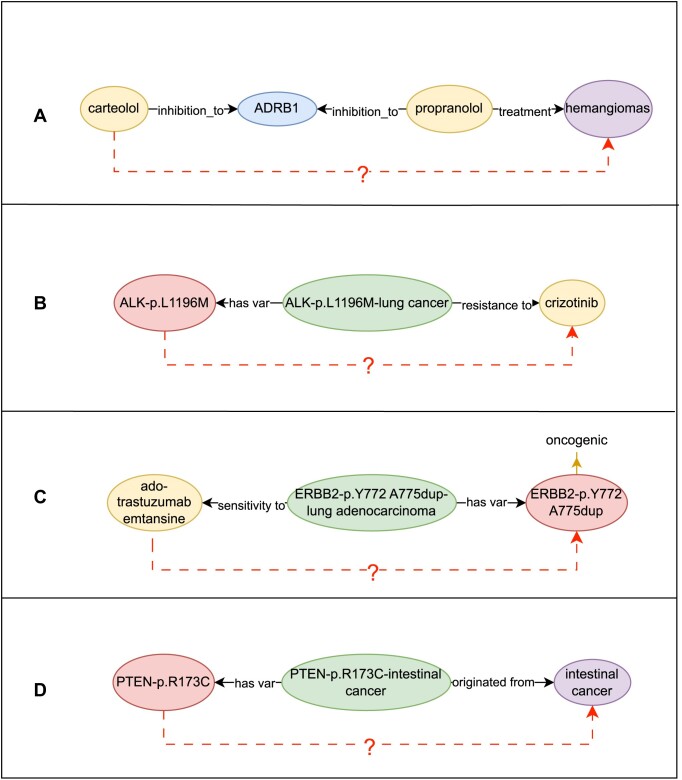
(A), (B), (C), and (D) respectively represent the relational diagrams of drug repositioning, drug resistance research, individualized treatment, and selection and understanding of biomarkers.

Drug repositioning emerges as a promising strategy to accelerate the process of drug development. This approach involves identifying new therapeutic uses for existing drugs, thereby saving time and resources typically required for bringing a new drug to market [45]. Our system is capable of investigating the potential repositioning of carteolol for the treatment of hemangiomas. The example is shown in Supplementary Table S2 and relational diagram is shown in Fig. 4A. Utilizing the system’s knowledge graph, a relational chain is delineated, illustrating that propranolol, another inhibitor of ADRB1, is effectively employed in the treatment of hemangiomas. The system harnesses this insight to formulate a hypothesis that carteolol, by virtue of its similar mechanism of inhibition, could be potentially repositioning for treating hemangiomas [46]. This hypothesis would serve as a precursor to clinical trials and research, potentially expediting the availability of an additional therapeutic option for patients with hemangiomas.

#### Drug resistance research

Drug resistance in cancer treatment poses a significant challenge in clinical oncology. Understanding the genetic basis of resistance can lead to more effective treatment strategies and personalized medicine approaches. Research in drug resistance involves determining why certain cancer-carrying mutated genes are not responsive to specific drugs and finding ways to overcome this resistance [47]. Our system is capable of exploring drug resistance in cancer. The example is shown in Supplementary Table S3, and a relational diagram is shown in Fig. 4B. The KG data indicate that the ALK-p.L1196M mutation, which is associated with gastric cancer, has a known resistance to nalatinib [48, 49]. The LLM processes this information and infers that due to this resistance, nalatinib might not be an effective medication for treating cancers caused by the ALK-p.L1196M mutation. The case highlights the critical importance of understanding specific gene–drug interactions in drug resistance research. It demonstrates how certain gene mutations could render a drug ineffective, which in turn could guide oncologists in choosing alternative treatments or developing new drugs that can bypass or target the resistance mechanisms. By accelerating the process of understanding drug resistance, these artificial intelligence–driven systems can contribute to improved patient outcomes and the optimization of cancer treatment protocols.

#### Individualized treatment

Details on individualized treatment are provided in Supplementary Case Studies A. It is important to note that this example is included solely to illustrate the technical capabilities of the proposed method. The output generated in this example has not been validated for clinical use, and further validation in clinical settings would be required before any such application.

#### Selection and understanding of biomarkers

Details on selection and understanding of biomarkers are provided in Supplementary Case Studies B.

## Discussion

In this article, we introduce a novel framework KGT, which employs LLMs for reasoning on the schema of KGs, to enhance the reasoning abilities of LLMs in areas with missing domain data by utilizing domain-specific knowledge graphs, such as oncology knowledge graphs, thereby addressing the issue of factual hallucinations in LLMs. Our method excels in extracting, validating, and refining factual knowledge throughout the LLMs’ reasoning process. It seamlessly integrates with various LLMs, including open-source models like Code-Llama, and enhances the capabilities of LLMs solely through prompt engineering and in-context learning without any fine-tuning. This grants it significant generalizability.

We possess an extensive oncology knowledge graph and have established a benchmark based on it to evaluate the capabilities of various methods. When tested on PcQA using various open-source LLMs, the KGT framework performs exceptionally well, surpassing the current best methods by 33%. This significant improvement positions our approach as a pioneering benchmark in biomedical KGQA, setting a new standard that advances beyond previously established best practices. Additionally, through case studies, our approach has been shown to effectively provide therapeutic plans, generate valuable hypotheses for drug repositioning, identify potential drug targets, and study drug resistance. This underscores the practical value of the KGT framework in delivering insightful contributions that aid in the development and optimization of treatment strategies. Each case study’s conclusions are further validated by evidence from previously published research papers, enhancing the credibility and impact of our findings.

However, it is important to note that the constructed QA dataset and the corresponding published subset of the SOKG were specifically designed to validate the effectiveness of the KGT framework within this study. While the dataset is highly relevant to biomedical applications, its scope is primarily focused on validating the proposed method. Therefore, it may not cover all potential use cases. Additionally, our system currently has the drawback of not performing fuzzy matching; if a drug name is misspelled by even 1 letter, it fails to retrieve information from the knowledge graph. Therefore, we plan to improve this aspect in the future to enhance the system’s usability and reliability. Our ultimate goal is to create a robust framework applicable to the rapidly evolving domain of medical knowledge, supporting health care professionals in delivering personalized, precise medication tailored to the individual needs of each patient.

Finally, we affirm that this study serves as a proof of concept, aiming to showcase the technical feasibility and initial efficacy of the method, which has not been validated in actual clinical practice. In any clinical or medical decision-making, reliance should always be placed on the judgment and guidance of professional health care practitioners.

## Additional Files

**Supplementary Table S1**. Comparison of PcQA with MetaQA and FACTKG in multihop tasks. The types of intermediate entities are indicated in bold.

**Supplementary Table S2**. Example of drug repositioning.

**Supplementary Table S3**. Example of drug resistance research.

**Supplementary Table S4**. Example of individualized treatment.

**Supplementary Table S5**. Example of selection and understanding of biomarkers.

**Supplementary Table S6**. Prompts for Base and CoT&ICL.

**Supplementary Fig. S1**. Prompt for key information extraction.

**Supplementary Fig. S2**. Prompt for query statement generation.

**Supplementary Fig. S3**. Prompt for LLM inference and output.

**Supplementary Fig. S4**. Prompt for GPT-4–based evaluation.

**Supplementary Fig. S5**. (A), (B), (C), and (D) respectively represent the relational diagrams of drug repositioning, drug resistance research, individualized treatment, and selection and understanding of biomarkers.

giae082_Supplemental_Files

giae082_GIGA-D-24-00191_Original_Submission

giae082_GIGA-D-24-00191_Revision_1

giae082_GIGA-D-24-00191_Revision_2

giae082_GIGA-D-24-00191_Revision_3 Stephen Richards -- 2/27/2023 Reviewed

giae082_GIGA-D-24-00191_Revision_4 Xuefeng Wang -- 4/3/2023 Reviewed

giae082_GIGA-D-24-00191_Revision_5

giae082_Response_to_Reviewer_Comments_evision_4

giae082_Response_to_Reviewer_Comments_Original_Submission

giae082_Response_to_Reviewer_Comments_Revision_1

giae082_Response_to_Reviewer_Comments_Revision_2

giae082_Response_to_Reviewer_Comments_Revision_3

giae082_Reviewer_1_Report_Original_Submission Linhao Luo -- 7/2/2024

giae082_Reviewer_1_Report_Revision_1 Linhao Luo -- 8/19/2024

giae082_Reviewer_1_Report_Revision_2 Linhao Luo -- 8/29/2024

giae082_Reviewer_2_Report_Original_Submission Cody Bumgardner -- 8/2/2024

## Abbreviations

APE: automatic prompt engineer; BFS: breadth-first search; CF: catastrophic forgetting; CoT: chain of thought; GPT: generative pretrained transformer; ICL: in-context learning; KG: knowledge graph; KGQA: knowledge graph question answering; LLM: large language model; NLP: natural language processing; PcQA: pan-cancer question answering; RLHF: reinforcement learning with human feedback; SFT: supervised fine-tuning.

## Availability of Source Code and Requirements

Project name: bioKGQA-KGT

- Project homepage: https://github.com/yichun10/bioKGQA-KGT.git
- Operating system(s): Linux (Ubuntu)
- Resource usage in inference step: A Linux (Ubuntu) system with at least 2 CPU cores and 32 GB of VRAM. The GPU card needs at least 60 GB VRAM (either two 32 GB V100s or one 80 GB A100)
- Programming language: Shell Script (Bash) with Python 3.10.13
- Other requirements: Python 3.10.13 with GPU/CPU support, neo4j 5.13.0 (please see more requirements on GitHub repository)
- Licenses: MIT license
- Research Resource Identifier (#RRID): SCR_025176

## Ethical Statement

This study involves the generation of a biomedical question-answer dataset derived from a biomedical knowledge graph developed by our team. The knowledge graph has been meticulously constructed using nonpersonalized data obtained from various credible biomedical sources. The data collection and utilization processes strictly comply with all relevant legal regulations and ethical guidelines, ensuring the highest standards of data security and privacy. The dataset adheres rigorously to data protection principles and contains no sensitive personal information or identifiable individual health data. Furthermore, as the data collection and processing activities in this study do not involve human subjects, this research did not require ethical review or approval.

## Data Availability

We have publicly provided a subset of the SmartQuerier Oncology Knowledge Graph necessary for reproducing the research. An archival copy of the code and the subgraph of the knowledge graph used in this research is available via Software Heritage [33], and the code and datasets can be accessed via GitHub [50]. Additionally, the prompts used in interactions with LLMs [1, 2, 38, 42–44] during this research are available in the supplemental material. For access to the complete SmartQuerier Oncology Knowledge Graph data, please contact at service@smartquerier.com.
