# Supplementary material for: Knowledge graph–based thought: a knowledge graph–enhanced LLM framework for pan-cancer question answering
Source: Gigascience. 2025 Jan 6;14:giae082. doi: 10.1093/gigascience/giae082 (PMC11702363; doi:10.1093/gigascience/giae082)
Supplement: giae082_Supplemental_Files [file giae082_supplemental_files.zip › Knowledge Graph-based Thought_Supplementary material.pdf]

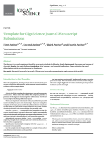
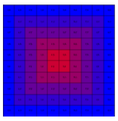

Journal of X, 2023, 1–5

Manuscript in Preparation
Supplementary material

SUPPLEMENTARY MATERIAL

Knowledge Graph-based Thought: a knowledge graph enhanced LLMs framework for pan-cancer question answering

Yichun Feng[1,2,†], Lu Zhou[2,†], Chao Ma[3,†], Yikai Zheng[2], Ruikun He[4,5,*] and Yixue Li[1,2,*]

[1]Hangzhou Institute for Advanced Study, University of Chinese Academy of Sciences, 310024 Hangzhou, China and [2]Guangzhou National Laboratory, No. 9 XingDaoHuanBei Road, Guangzhou International Bio Island, 510005 Guangzhou, China and [3]Smartquerier Gene Technology (Shanghai) Co., Ltd., 200100 Shanghai, China and [4]BYHEALTH Institute of Nutrition & Health, 510663 Guangzhou, China and [5]Shanghai Institute of Nutrition and Health, Chinese Academy of Sciences Shanghai, 200030 Shanghai, China

[*]Correspondence address. Yixue Li, Guangzhou National Laboratory, No. 9 XingDaoHuanBei Road, Guangzhou International Bio Island, 510005 Guangzhou, China. E-mail: yxli@sibs.ac.cn; Ruikun He, BYHEALTH Institute of Nutrition & Health, 510663 Guangzhou, China. E-mail: herk@by-health.com
[†]Contributed equally.

Abstract

This is the document for supplementary materials, which contains related attachments for 'Knowledge Graph-based Thought: a knowledge graph enhanced LLMs framework for pan-cancer question answering'

Supplementary material

Case studies A: Individualized treatment

The advancement of personalized medicine is predicated on the ability to tailor treatments to individual patient characteristics [1], particularly their genetic makeup. Individualized treatment is crucial for optimizing therapeutic efficacy and minimizing adverse effects. In oncology, this approach is particularly important as genetic variations can significantly influence how a patient responds to a drug. Our system is capable of providing individualized treatment plans. The example is shown in Table. S4 and relational diagram is shown in Figure. S5(C). The KG provides a relational chain that establishes the sensitivity of lung adenocarcinoma cells harboring the ERBB2-p.Y772_A775dup mutation to ado-trastuzumab emtansine [2]. Through reasoning paths, the system identifies that ado-trastuzumab emtansine has efficacy against lung adenocarcinoma characterized by this particular mutation [3]. This case exemplifies the importance of individualized treatment. Our question-answering system can rapidly identify actionable genetic mutations and suitable drug therapies, enabling clinicians to prescribe treatment plans based on the genetic characteristics of a patient's tumor, thereby achieving more precise and potentially more successful cancer treatments.

Case studies B: Selection and understanding of biomarkers

Biomarkers play a crucial role in disease diagnosis, prognosis, and therapeutic interventions [4]. The selection and understanding of biomarkers are essential for tailoring patient-specific treatment strategies. However, the vast amount of biological data presents a significant challenge for clinicians and researchers in identifying relevant biomarkers efficiently. Our system is capable of discerning the impact of the PTEN-p.R173C mutation as a biomarker for intestinal cancer. The example is shown in Table. S5 and relational diagram is shown in Figure. S5(D). Using the relational chain from the

Compiled on: May 27, 2024.
Draft manuscript prepared by the author.

Table S1. Comparison of PcQA with MetaQA and FACTKG in Multi-hop Tasks. The types of intermediate entities are indicated in bold.

Datasets	Question	Retrieved Graph	Prediction
MetaQA	when did the **films** starred by [Deborah Van Valkenburgh] release?	[‘Mean Guns’, starred_actors, ‘Deborah Van Valkenburgh’], [‘Mean Guns’, release_year, ‘1997’]	‘1997’
FACTKG	Alfredo Zitarrosa died in a **city**, Uruguay (which has Raul Fernando Sendic Rodriguez as leader).	[‘Alfredo_Zitarrosa’, deathPlace, ‘Uruguay’], [‘Uruguay’, leader, ‘Raul_Fernando_Sendic_Rodriguez’]	Supported
PcQA(ours)	What oncogenic genetic mutations are present in adenoid cystic carcinoma?	[‘adenoid cystic carcinoma’, originated_from, ‘FLT3-p.D835Y-adenoid cystic carcinoma’], [‘FLT3-p.D835Y-adenoid cystic carcinoma’, has_var, ‘FLT3-p.D835Y’], [‘FLT3-p.D835Y’: Oncogenic]	‘FLT3-p.D835Y’

Table S2. Example of drug repositioning

**Question**	Is carteolol effective in treating hemangioma?
**Reasoning Paths**	Drug - [inhibition_to] ⟶ Genesymbol ⟵ [inhibition_to] - Drug - [treatment]⟶ Cancer
**Relational Chain**	(carteolol) – [inhibition_to] ⟶ (ADRB1) ⟵ [inhibition_to] – (propranolol) – [treatment] ⟶ (hemangiomas)
**Inference**	Carteolol inhibits ADRB1.Propranolol also inhibits ADRB1 and is used in the treatment of hemangiomas.This relational chain suggests that by inhibiting ADRB1, propranolol can serve as a treatment method for hemangiomas. carteolol, operating through the same mechanism of inhibition (inhibiting ADRB1), allows us to infer that carteolol may have some efficacy in the treatment of hemangiomas.
**Answer**	Carteolol may have some efficacy in treating hemangiomas due to its mechanism of inhibiting ADRB1, the same target inhibited by propranolol, which is used for hemangiomas treatment.

KG, the system reveals that the PTEN-p.R173C mutation is present in certain cancer cells of intestinal cancer and that these cells originate from intestinal cancer, indicating the mutation’s potential role as a driver for this type of cancer [5, 6]. The inference made by the system emphasizes the significance of the PTEN-p.R173C mutation as a biomarker for intestinal cancer. This providing valuable insights for clinical decision-making.

Key Information Extraction Prompt

You need to follow these three steps based on your biomedical knowledge:
step1:
Extract the name of the head entity and the type of the tail entity in my question,with the head defined as the active voice of the problem and the tail defined as the passive voice of the problem. The entity types include ...
step2:
Based on the entity type from step 1, select an attribute from the attribute list that best fits my question.
step3:
If there is only one head entity name, the output format should be (head entity name, tail entity type, attribute); if there are two head entity names, the output format should be (head entity name 1,tail entity type, attribute), (head entity name 2,tail entity type, attribute).
Examples

Figure S1. Prompt for key information extraction.

Text2Cypher Prompt

Given a knowledge graph, create a Cypher statement to answer the following question.
Examples

Figure S2. Prompt for query statement generation.

LLMs Inference and Output Prompt

You are a reasoning robot, and you need to perform the following two steps step by step:
step1: Output a corresponding natural language sentence for each relational chain.
step2: Answer the question using natural language from step1.
Examples

Figure S3. Prompt for LLMs inference and output.

GPT4-based Evaluation Prompt

I have two sentences: my-answer and standard-answer. You need to rate their similarity, and if they express exactly the same meaning, give them 100 points; If the expressed meaning is opposite, give 0 points; If there are any unreasonable aspects, corresponding points will be deducted and specific scores will be given.

Figure S4. Prompt for GPT-4-based evaluation.

Table S3. Example of drug resistance research

**Question**	Which drugs does cancer carrying ALK-p.L1196M exhibit resistance?
**Reasoning Paths**	SnvFull ⟵ [has_var] - CancerCell - [resistance_to]⟶ Drug
**Relational Chain**	(ALK-p.L1196M) ⟵ [has_var] – (ALK-p.L1196M-lung cancer) – [resistance_to] ⟶ (crizotinib)
**Inference**	ALK-p.L1196M is a gene mutation. The term "has var" indicates that the ALK-p.L1196M mutation is present in the context related to lung cancer. "Resistance to" denotes that this ALK-p.L1196M variant, associated with lung cancer, exhibits resistance to the crizotinib. Therefore, from this chain of relations, it can be concluded that ALK-p.L1196M demonstrates resistance to crizotinib in lung cancer, implying that crizotinib might not be an effective medication for treating cancers caused by this specific gene mutation.
**Answer**	The cancer carrying ALK-p.L1196M shows resistance to crizotinib.

Table S4. Example of individualized treatment

**Question**	Which oncogenic gene mutation can be treated by ado-trastuzumab emtansine?
**Attribute**	snvfull.oncogenic
**Reasoning Paths**	Drug ⟵ [sensitivity_to] - CancerCell - [has_var]⟶ SnvFull
**Relational Chain**	(ado-trastuzumab emtansine) ⟵ [sensitivity_to] – (ERBB2-p.Y772_A775dup-lung adenocarcinoma) – [has_var] ⟶ (ERBB2-p.Y772_A775dup) oncogenic
**Inference**	Ado-trastuzumab emtansine is sensitive to lung adenocarcinoma cells with the ERBB2-p.Y772_A775dup mutation. This sensitivity suggests that ado-trastuzumab emtansine may be effective in inhibiting or treating the proliferation of lung adenocarcinoma cells with this mutation. Therefore, it can be deduced that ERBB2-p.Y772_A775dup is an oncogenic gene mutation that can be treated with ado-trastuzumab emtansine.
**Answer**	The oncogenic mutation gene that can be treated with ado-trastuzumab emtansine is ERBB2-p.Y772_A775dup.

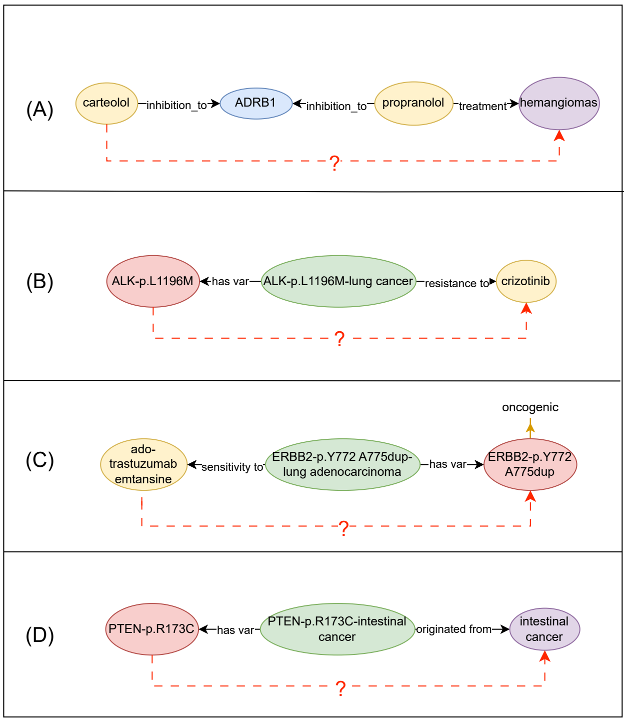

Figure S5. (A), (B), (C) and (D) respectively represent the relational diagrams of drug repositioning, drug resistance research, individualized treatmen and selection and understanding of biomarkers.

Table S5. Example of selection and understanding of biomarkers

**Question**	What type of cancers can be driven by PTEN-p.R173C?
**Reasoning Paths**	SnvFull ⟵ [has_var] - CancerCell - [originated_from]⟶ Cancer
**Relational Chain**	(PTEN-p.R173C) ⟵ [has_var] – (PTEN-p.R173C-intestinal cancer) – [originated_from] ⟶ (intestinal cancer)
**Inference**	PTEN-p.R173C is a specific genetic mutation that exists in certain cancer cells of intestinal cancer, and these cancer cells originate from intestinal cancer, indicating that this mutated gene can drive this cancer.
**Answer**	PTEN-p.R173C can drive intestinal cancer.

Table S6. Prompts for Base and CoT&ICL.

	Base	CoT&ICL
Text2Cypher Prompt	Given a knowledge graph, create a Cypher statement to answer the question	Given a knowledge graph, you need to perform the following two steps step by step: step1: Select the optimal path and attributes from the knowledge graph based on the question. step2: Create a Cypher statement to answer the question.<br>Examples
Inference Prompt	Answer my question based on the relational chain.	You need to perform the following two steps step by step: step1: Output a corresponding natural language sentence for each relational chain. step2: Answer my question using natural language from step 1.<br>Examples

References

1. Stock G, Sydow S. Personalised medicine: Paradigm shift within drug research and therapy. Bundesgesundheitsblatt-Gesundheitsforschung-Gesundheitsschutz 2013;56:1495–1501.
2. Wang H, He Y, Zhao W, Tong Z. Ado-trastuzumab emtansine in the treatment of lung adenocarcinoma with ERBB2 mutation: a case report and literature review. Anti-cancer drugs 2022;33(8):773–777.
3. Huang X, Jin R, Lou L, Zhao J, Xia L, Zhao J, et al. The efficacy of ado-trastuzumab emtansine in patients with ERBB2-aberrant non-small cell lung cancer: a systematic review. Translational Cancer Research 2020;9(8):4507.
4. Bodaghi A, Fattahi N, Ramazani A. Biomarkers: Promising and valuable tools towards diagnosis, prognosis and treatment of Covid-19 and other diseases. Heliyon 2023;.
5. Davies EJ, Marsh Durban V, Meniel V, Williams GT, Clarke AR. PTEN loss and KRAS activation leads to the formation of serrated adenomas and metastatic carcinoma in the mouse intestine. The Journal of pathology 2014;233(1):27–38.
6. Yang Z, Yuan XG, Chen J, Luo SW, Luo ZJ, Lu NH. Reduced expression of PTEN and increased PTEN phosphorylation at residue Ser380 in gastric cancer tissues: a novel mechanism of PTEN inactivation. Clinics and research in hepatology and gastroenterology 2013;37(1):72–79.
