# Supplementary material for: Knowledge graph–based thought: a knowledge graph–enhanced LLM framework for pan-cancer question answering
Source: Gigascience. 2025 Jan 6;14:giae082. doi: 10.1093/gigascience/giae082 (PMC11702363; doi:10.1093/gigascience/giae082)
Supplement: giae082_GIGA-D-24-00191_Revision_5 [file giae082_giga-d-24-00191_revision_5.pdf]

GigaScience

Knowledge Graph-based Thought: a knowledge graph enhanced LLMs framework for pan-cancer question answering

--Manuscript Draft--

**Manuscript Number:**	GIGA-D-24-00191R5
**Full Title:**	Knowledge Graph-based Thought: a knowledge graph enhanced LLMs framework for pan-cancer question answering
**Article Type:**	Research
**Funding Information:**	National Key Research and Development Program of China (No. 2022YFF1202101, 2023YFC3041600) — Ph.D. Yixue Li<br>Chinese Academy of Sciences Research Fund (No. XDB38050200) — Ph.D. Yixue Li<br>the Self-supporting Program of Guangzhou National Laboratory (No. SRPG22007，No. SRPG22001) — Ph.D. Yixue Li
**Abstract:**	Background. In recent years, Large Language Models (LLMs) have shown promise in various domains, notably in biomedical sciences. However, their real-world application is often limited by issues like erroneous outputs and hallucinatory responses.<br>Results. We developed the Knowledge Graph-based Thought (KGT) framework, an innovative solution that integrates LLMs with Knowledge Graphs (KGs) to improve their initial responses by utilizing verifiable information from KGs, thus significantly reducing factual errors in reasoning. The KGT framework demonstrates strong adaptability and performs well across various open-source LLMs. Notably, KGT can facilitate the discovery of new uses for existing drugs through potential drug-cancer associations, and can assist in predicting resistance by analyzing relevant biomarkers and genetic mechanisms. To evaluate the Knowledge Graph Question Answering (KGQA) task within biomedicine, we utilize a pan-cancer knowledge graph to develop a pan-cancer question answering benchmark, named the Pan-cancer Question Answering (PcQA).<br>Conclusions. The KGT framework substantially improves the accuracy and utility of LLMs in the biomedical field. This study serves as a proof-of-concept, demonstrating its exceptional performance in biomedical question answering.
**Corresponding Author:**	Yichun Feng<br>University of the Chinese Academy of Sciences<br>Hangzhou, CHINA
**Corresponding Author Secondary Information:**	
**Corresponding Author's Institution:**	University of the Chinese Academy of Sciences
**Corresponding Author's Secondary Institution:**	
**First Author:**	Yichun Feng
**First Author Secondary Information:**	
**Order of Authors:**	Yichun Feng<br>Lu Zhou<br>Chao Ma<br>Yikai Zheng<br>Ruikun He<br>Yixue Li
**Order of Authors Secondary Information:**	

Powered by Editorial Manager® and ProduXion Manager® from Aries Systems Corporation

**Response to Reviewers:**	Thanks for your suggestion. We have added the ORCIDs for the authors to the title page. The revised LaTeX source files, including the manuscript, bibliography, style files, and figures, have been uploaded to the Editorial Manager as requested.
**Additional Information:**	
**Question**	**Response**
Are you submitting this manuscript to a special series or article collection?	No
**Experimental design and statistics**<br><br>Full details of the experimental design and statistical methods used should be given in the Methods section, as detailed in our Minimum Standards Reporting Checklist. Information essential to interpreting the data presented should be made available in the figure legends.<br><br>Have you included all the information requested in your manuscript?	Yes
**Resources**<br><br>A description of all resources used, including antibodies, cell lines, animals and software tools, with enough information to allow them to be uniquely identified, should be included in the Methods section. Authors are strongly encouraged to cite Research Resource Identifiers (RRIDs) for antibodies, model organisms and tools, where possible.<br><br>Have you included the information requested as detailed in our Minimum Standards Reporting Checklist?	Yes
**Availability of data and materials**<br><br>All datasets and code on which the conclusions of the paper rely must be either included in your submission or deposited in publicly available repositories (where available and ethically appropriate), referencing such data using a unique identifier in the references and in	Yes

the “Availability of Data and Materials” section of your manuscript.<br><br>Have you have met the above requirement as detailed in our Minimum Standards Reporting Checklist?	

Powered by Editorial Manager® and ProduXion Manager® from Aries Systems Corporation

LaTeX - supplementary Document (TeX file) Click here to access/download;LaTeX - Main Document (TeX file);supplementary.tex

```
This is pdfTeX, Version 3.141592653-2.6-1.40.25 (TeX Live 2023) 
(preloaded format=pdflatex 2024.3.8)  1 OCT 2024 04:11
entering extended mode
 restricted \write18 enabled.
 %&-line parsing enabled.
**supplementary.tex
(./supplementary.tex
LaTeX2e <2023-11-01> patch level 1
L3 programming layer <2024-02-20>
(./oup-contemporary.cls
Document Class: oup-contemporary 2023/06/12, v1.2
(c:/texlive/2023/texmf-dist/tex/latex/base/article.cls
Document Class: article 2023/05/17 v1.4n Standard LaTeX document class
(c:/texlive/2023/texmf-dist/tex/latex/base/size10.clo
File: size10.clo 2023/05/17 v1.4n Standard LaTeX file (size option)
)
\c@part=\count188
\c@section=\count189
\c@subsection=\count190
\c@subsubsection=\count191
\c@paragraph=\count192
\c@subparagraph=\count193
\c@figure=\count194
\c@table=\count195
\abovecaptionskip=\skip48
\belowcaptionskip=\skip49
\bibindent=\dimen140
) (c:/texlive/2023/texmf-dist/tex/latex/base/inputenc.sty
Package: inputenc 2021/02/14 v1.3d Input encoding file
\inpenc@prehook=\toks17
\inpenc@posthook=\toks18
) (c:/texlive/2023/texmf-dist/tex/latex/base/fontenc.sty
Package: fontenc 2021/04/29 v2.0v Standard LaTeX package
) (c:/texlive/2023/texmf-dist/tex/generic/iftex/ifpdf.sty
Package: ifpdf 2019/10/25 v3.4 ifpdf legacy package. Use iftex instead.
(c:/texlive/2023/texmf-dist/tex/generic/iftex/iftex.sty
Package: iftex 2022/02/03 v1.0f TeX engine tests
)) (c:/texlive/2023/texmf-dist/tex/latex/microtype/microtype.sty
Package: microtype 2023/03/13 v3.1a Micro-typographical refinements (RS)
(c:/texlive/2023/texmf-dist/tex/latex/graphics/keyval.sty
Package: keyval 2022/05/29 v1.15 key=value parser (DPC)
\KV@toks@=\toks19
) (c:/texlive/2023/texmf-dist/tex/latex/etoolbox/etoolbox.sty
Package: etoolbox 2020/10/05 v2.5k e-TeX tools for LaTeX (JAW)
\etb@tempcnta=\count196
)
\MT@toks=\toks20
\MT@tempbox=\box51
\MT@count=\count197
LaTeX Info: Redefining \noprotrusionifhmode on input line 1059.
LaTeX Info: Redefining \leftprotrusion on input line 1060.
\MT@prot@toks=\toks21
LaTeX Info: Redefining \rightprotrusion on input line 1078.
LaTeX Info: Redefining \textls on input line 1368.
```

```
\MT@outer@kern=\dimen141
LaTeX Info: Redefining \textmicrotypecontext on input line 1988.
\MT@listname@count=\count198
(c:/texlive/2023/texmf-dist/tex/latex/microtype/microtype-pdftex.def
File: microtype-pdftex.def 2023/03/13 v3.1a Definitions specific to
pdftex (RS)

LaTeX Info: Redefining \lsstyle on input line 902.
LaTeX Info: Redefining \lslig on input line 902.
\MT@outer@space=\skip50
)
Package microtype Info: Loading configuration file microtype.cfg.
(c:/texlive/2023/texmf-dist/tex/latex/microtype/microtype.cfg
File: microtype.cfg 2023/03/13 v3.1a microtype main configuration file
(RS)
)) (c:/texlive/2023/texmf-dist/tex/latex/euler/euler.sty
Package: euler 1995/03/05 v2.5
Package: `euler' v2.5 <1995/03/05> (FJ and FMi)
LaTeX Font Info:    Redeclaring symbol font `letters' on input line 35.
LaTeX Font Info:    Encoding `OML' has changed to `U' for symbol font
(Font)              `letters' in the math version `normal' on input line
35.
LaTeX Font Info:    Overwriting symbol font `letters' in version `normal'
(Font)                  OML/cmm/m/it --> U/eur/m/n on input line 35.
LaTeX Font Info:    Encoding `OML' has changed to `U' for symbol font
(Font)              `letters' in the math version `bold' on input line
35.
LaTeX Font Info:    Overwriting symbol font `letters' in version `bold'
(Font)                  OML/cmm/b/it --> U/eur/m/n on input line 35.
LaTeX Font Info:    Overwriting symbol font `letters' in version `bold'
(Font)                  U/eur/m/n --> U/eur/b/n on input line 36.
LaTeX Font Info:    Redeclaring math symbol \Gamma on input line 47.
LaTeX Font Info:    Redeclaring math symbol \Delta on input line 48.
LaTeX Font Info:    Redeclaring math symbol \Theta on input line 49.
LaTeX Font Info:    Redeclaring math symbol \Lambda on input line 50.
LaTeX Font Info:    Redeclaring math symbol \Xi on input line 51.
LaTeX Font Info:    Redeclaring math symbol \Pi on input line 52.
LaTeX Font Info:    Redeclaring math symbol \Sigma on input line 53.
LaTeX Font Info:    Redeclaring math symbol \Upsilon on input line 54.
LaTeX Font Info:    Redeclaring math symbol \Phi on input line 55.
LaTeX Font Info:    Redeclaring math symbol \Psi on input line 56.
LaTeX Font Info:    Redeclaring math symbol \Omega on input line 57.
\symEulerFraktur=\mathgroup4
LaTeX Font Info:    Overwriting symbol font `EulerFraktur' in version
`bold'
(Font)                  U/euf/m/n --> U/euf/b/n on input line 63.
LaTeX Info: Redefining \oldstylenums on input line 85.
\symEulerScript=\mathgroup5
LaTeX Font Info:    Overwriting symbol font `EulerScript' in version
`bold'
(Font)                  U/eus/m/n --> U/eus/b/n on input line 93.
LaTeX Font Info:    Redeclaring math symbol \aleph on input line 97.
LaTeX Font Info:    Redeclaring math symbol \Re on input line 98.
LaTeX Font Info:    Redeclaring math symbol \Im on input line 99.
```

```
LaTeX Font Info:    Redeclaring math delimiter \vert on input line 101.
LaTeX Font Info:    Redeclaring math delimiter \backslash on input line
103.
LaTeX Font Info:    Redeclaring math symbol \neg on input line 106.
LaTeX Font Info:    Redeclaring math symbol \wedge on input line 108.
LaTeX Font Info:    Redeclaring math symbol \vee on input line 110.
LaTeX Font Info:    Redeclaring math symbol \setminus on input line 112.
LaTeX Font Info:    Redeclaring math symbol \sim on input line 113.
LaTeX Font Info:    Redeclaring math symbol \mid on input line 114.
LaTeX Font Info:    Redeclaring math delimiter \arrowvert on input line
116.
LaTeX Font Info:    Redeclaring math symbol \mathsection on input line
117.
\symEulerExtension=\mathgroup6
LaTeX Font Info:    Redeclaring math symbol \coprod on input line 125.
LaTeX Font Info:    Redeclaring math symbol \prod on input line 125.
LaTeX Font Info:    Redeclaring math symbol \sum on input line 125.
LaTeX Font Info:    Redeclaring math symbol \intop on input line 130.
LaTeX Font Info:    Redeclaring math symbol \ointop on input line 131.
LaTeX Font Info:    Redeclaring math symbol \braceld on input line 132.
LaTeX Font Info:    Redeclaring math symbol \bracerd on input line 133.
LaTeX Font Info:    Redeclaring math symbol \bracelu on input line 134.
LaTeX Font Info:    Redeclaring math symbol \braceru on input line 135.
LaTeX Font Info:    Redeclaring math symbol \infty on input line 136.
LaTeX Font Info:    Redeclaring math symbol \nearrow on input line 153.
LaTeX Font Info:    Redeclaring math symbol \searrow on input line 154.
LaTeX Font Info:    Redeclaring math symbol \nwarrow on input line 155.
LaTeX Font Info:    Redeclaring math symbol \swarrow on input line 156.
LaTeX Font Info:    Redeclaring math symbol \Leftrightarrow on input line
157.
LaTeX Font Info:    Redeclaring math symbol \Leftarrow on input line 158.
LaTeX Font Info:    Redeclaring math symbol \Rightarrow on input line
159.
LaTeX Font Info:    Redeclaring math symbol \leftrightarrow on input line
160.
LaTeX Font Info:    Redeclaring math symbol \leftarrow on input line 161.
LaTeX Font Info:    Redeclaring math symbol \rightarrow on input line
163.
LaTeX Font Info:    Redeclaring math delimiter \uparrow on input line
166.
LaTeX Font Info:    Redeclaring math delimiter \downarrow on input line
168.
LaTeX Font Info:    Redeclaring math delimiter \updownarrow on input line
170.
LaTeX Font Info:    Redeclaring math delimiter \Uparrow on input line
172.
LaTeX Font Info:    Redeclaring math delimiter \Downarrow on input line
174.
LaTeX Font Info:    Redeclaring math delimiter \Updownarrow on input line
176.
LaTeX Font Info:    Redeclaring math symbol \leftharpoonup on input line
177.
LaTeX Font Info:    Redeclaring math symbol \leftharpoondown on input
line 178.
```

```
LaTeX Font Info:    Redeclaring math symbol \rightharpoonup on input line
179.
LaTeX Font Info:    Redeclaring math symbol \rightharpoondown on input
line 180
.
LaTeX Font Info:    Redeclaring math delimiter \lbrace on input line 182.
LaTeX Font Info:    Redeclaring math delimiter \rbrace on input line 184.
\symcmmigroup=\mathgroup7
LaTeX Font Info:    Overwriting symbol font `cmmigroup' in version `bold'
(Font)                  OML/cmm/m/it --> OML/cmm/b/it on input line 200.
LaTeX Font Info:    Redeclaring math accent \vec on input line 201.
LaTeX Font Info:    Redeclaring math symbol \triangleleft on input line
202.
LaTeX Font Info:    Redeclaring math symbol \triangleright on input line
203.
LaTeX Font Info:    Redeclaring math symbol \star on input line 204.
LaTeX Font Info:    Redeclaring math symbol \lhook on input line 205.
LaTeX Font Info:    Redeclaring math symbol \rhook on input line 206.
LaTeX Font Info:    Redeclaring math symbol \flat on input line 207.
LaTeX Font Info:    Redeclaring math symbol \natural on input line 208.
LaTeX Font Info:    Redeclaring math symbol \sharp on input line 209.
LaTeX Font Info:    Redeclaring math symbol \smile on input line 210.
LaTeX Font Info:    Redeclaring math symbol \frown on input line 211.
LaTeX Font Info:    Redeclaring math accent \grave on input line 245.
LaTeX Font Info:    Redeclaring math accent \acute on input line 246.
LaTeX Font Info:    Redeclaring math accent \tilde on input line 247.
LaTeX Font Info:    Redeclaring math accent \ddot on input line 248.
LaTeX Font Info:    Redeclaring math accent \check on input line 249.
LaTeX Font Info:    Redeclaring math accent \breve on input line 250.
LaTeX Font Info:    Redeclaring math accent \bar on input line 251.
LaTeX Font Info:    Redeclaring math accent \dot on input line 252.
LaTeX Font Info:    Redeclaring math accent \hat on input line 254.
) (c:/texlive/2023/texmf-dist/tex/latex/multirow/multirow.sty
Package: multirow 2021/03/15 v2.8 Span multiple rows of a table
\multirow@colwidth=\skip51
\multirow@cntb=\count199
\multirow@dima=\skip52
\bigstrutjot=\dimen142
) (c:/texlive/2023/texmf-dist/tex/latex/algorithms/algorithm.sty
Package: algorithm 2009/08/24 v0.1 Document Style `algorithm' - floating
enviro
nment
(c:/texlive/2023/texmf-dist/tex/latex/float/float.sty
Package: float 2001/11/08 v1.3d Float enhancements (AL)
\c@float@type=\count266
\float@exts=\toks22
\float@box=\box52
\@float@everytoks=\toks23
\@floatcapt=\box53
) (c:/texlive/2023/texmf-dist/tex/latex/base/ifthen.sty
Package: ifthen 2022/04/13 v1.1d Standard LaTeX ifthen package (DPC)
)
\@float@every@algorithm=\toks24
```

```
\c@algorithm=\count267
) (c:/texlive/2023/texmf-dist/tex/latex/algorithmicx/algorithmicx.sty
Package: algorithmicx 2005/04/27 v1.2 Algorithmicx
Document Style algorithmicx 1.2 - a greatly improved `algorithmic' style
\c@ALG@line=\count268
\c@ALG@rem=\count269
\c@ALG@nested=\count270
\ALG@tlm=\skip53
\ALG@thistlm=\skip54
\c@ALG@Lnr=\count271
\c@ALG@blocknr=\count272
\c@ALG@storecount=\count273
\c@ALG@tmpcounter=\count274
\ALG@tmplength=\skip55
) (c:/texlive/2023/texmf-dist/tex/latex/merriweather/merriweather.sty
Package: merriweather 2022/09/20 (Bob Tennent) Supports
Merriweather(Sans) font
s for all LaTeX engines.
(c:/texlive/2023/texmf-dist/tex/generic/iftex/ifxetex.sty
Package: ifxetex 2019/10/25 v0.7 ifxetex legacy package. Use iftex
instead.
) (c:/texlive/2023/texmf-dist/tex/generic/iftex/ifluatex.sty
Package: ifluatex 2019/10/25 v1.5 ifluatex legacy package. Use iftex
instead.
) (c:/texlive/2023/texmf-dist/tex/latex/base/textcomp.sty
Package: textcomp 2020/02/02 v2.0n Standard LaTeX package
) (c:/texlive/2023/texmf-dist/tex/latex/xkeyval/xkeyval.sty
Package: xkeyval 2022/06/16 v2.9 package option processing (HA)
(c:/texlive/2023/texmf-dist/tex/generic/xkeyval/xkeyval.tex
(c:/texlive/2023/te
xmf-dist/tex/generic/xkeyval/xkvutils.tex
\XKV@toks=\toks25
\XKV@tempa@toks=\toks26
)
\XKV@depth=\count275
File: xkeyval.tex 2014/12/03 v2.7a key=value parser (HA)
)) (c:/texlive/2023/texmf-dist/tex/latex/base/fontenc.sty
Package: fontenc 2021/04/29 v2.0v Standard LaTeX package
) (c:/texlive/2023/texmf-dist/tex/latex/fontaxes/fontaxes.sty
Package: fontaxes 2020/07/21 v1.0e Font selection axes
LaTeX Info: Redefining \upshape on input line 29.
LaTeX Info: Redefining \itshape on input line 31.
LaTeX Info: Redefining \slshape on input line 33.
LaTeX Info: Redefining \swshape on input line 35.
LaTeX Info: Redefining \scshape on input line 37.
LaTeX Info: Redefining \sscshape on input line 39.
LaTeX Info: Redefining \ulcshape on input line 41.
LaTeX Info: Redefining \textsw on input line 47.
LaTeX Info: Redefining \textssc on input line 48.
LaTeX Info: Redefining \textulc on input line 49.
)) (c:/texlive/2023/texmf-dist/tex/latex/mathastext/mathastext.sty
Package: mathastext 2023/12/29 v1.3zb Use the text font in math mode
(JFB)
```

```
Package mathastext Info: Starting the math mode configuration.
\mst@exists@muskip=\muskip16
\mst@forall@muskip=\muskip17
\mst@prime@muskip=\muskip18
\mst@do@nonletters=\toks27
\mst@do@easynonletters=\toks28
\mst@do@az=\toks29
\mst@do@AZ=\toks30
\symmtoperatorfont=\mathgroup8
\symmtletterfont=\mathgroup9
(   mathastext:   ) ! and ?
(   mathastext:   ) punctuation: , . : ; and \colon
LaTeX Info: Redefining \relbar on input line 894.
LaTeX Info: Redefining \rightarrowfill on input line 897.
LaTeX Info: Redefining \leftarrowfill on input line 902.
(   mathastext:   ) + and =
LaTeX Info: Redefining \Relbar on input line 993.
(   mathastext:   ) adding = ; and + to \nfss@catcodes
(   mathastext:   ) parentheses ( ) [ ] and slash /
(   mathastext:   ) alldelims: < > \backslash \setminus | \vert \mid \{
\}
LaTeX Font Info:    Redeclaring math delimiter \backslash on input line
1039.
LaTeX Font Info:    Redeclaring math symbol \setminus on input line 1051.
LaTeX Info: Redefining \models on input line 1060.
(   mathastext:   ) \# \mathdollar \% \&
(   mathastext:   ) \imath and \jmath
LaTeX Font Info:    Overwriting math alphabet `\Mathnormalbold' in
version `nor
mal'
(Font)                  T1/Merriwthr-OsF/b/it --> T1/Merriwthr-OsF/b/it
on inpu
t line 2516.
LaTeX Font Info:    Overwriting math alphabet `\Mathnormalbold' in
version `bol
d'
(Font)                  T1/Merriwthr-OsF/b/it --> T1/Merriwthr-OsF/b/it
on inpu
t line 2516.
LaTeX Font Info:    Overwriting symbol font `mtletterfont' in version
`normal'
(Font)                  T1/Merriwthr-OsF/m/it --> T1/Merriwthr-OsF/m/it
on inpu
t line 2516.
LaTeX Font Info:    Overwriting symbol font `mtletterfont' in version
`bold'
(Font)                  T1/Merriwthr-OsF/m/it --> T1/Merriwthr-OsF/b/it
on inpu
t line 2516.
LaTeX Font Info:    Overwriting symbol font `mtoperatorfont' in version
`normal
'
(Font)                  T1/Merriwthr-OsF/m/n --> T1/Merriwthr-OsF/m/n on
input
```

```
line 2516.
LaTeX Font Info:    Overwriting symbol font `mtoperatorfont' in version
`bold'
(Font)                  T1/Merriwthr-OsF/m/n --> T1/Merriwthr-OsF/b/n on
input
line 2516.
LaTeX Font Info:    Overwriting math alphabet `\Mathbf' in version
`normal'
(Font)                  T1/Merriwthr-OsF/b/n --> T1/Merriwthr-OsF/b/n on
input
line 2516.
LaTeX Font Info:    Overwriting math alphabet `\Mathbf' in version `bold'
(Font)                  T1/Merriwthr-OsF/b/n --> T1/Merriwthr-OsF/b/n on
input
line 2516.
LaTeX Font Info:    Overwriting math alphabet `\Mathit' in version
`normal'
(Font)                  T1/Merriwthr-OsF/m/it --> T1/Merriwthr-OsF/m/it
on inpu
t line 2516.
LaTeX Font Info:    Overwriting math alphabet `\Mathit' in version `bold'
(Font)                  T1/Merriwthr-OsF/m/it --> T1/Merriwthr-OsF/b/it
on inpu
t line 2516.
LaTeX Font Info:    Overwriting math alphabet `\Mathsf' in version
`normal'
(Font)                  T1/MerriwthrSans-OsF/m/n --> T1/MerriwthrSans-
OsF/m/n o
n input line 2516.
LaTeX Font Info:    Overwriting math alphabet `\Mathsf' in version `bold'
(Font)                  T1/MerriwthrSans-OsF/m/n --> T1/MerriwthrSans-
OsF/b/n o
n input line 2516.
LaTeX Font Info:    Overwriting math alphabet `\Mathtt' in version
`normal'
(Font)                  T1/lmtt/m/n --> T1/lmtt/m/n on input line 2516.
LaTeX Font Info:    Overwriting math alphabet `\Mathtt' in version `bold'
(Font)                  T1/lmtt/m/n --> T1/lmtt/b/n on input line 2516.
(   mathastext:   ) Latin letters in the `normal', resp. `bold',
(   mathastext:   ) math versions are now set up to use the fonts
(   mathastext:   ) T1/Merriwthr-OsF/m/it, resp. T1/Merriwthr-OsF/b/it.
(   mathastext:   ) Other characters (digits, ...) and \log-like names
will be
(   mathastext:   ) typeset with the n shape.
(   mathastext:   ) \hbar
(   mathastext:   ) minus as endash
(   mathastext:   ) The italic option is in effect.
(   mathastext:   ) \HUGE has been (re)-defined.
(   mathastext:   ) mathastext has declared larger sizes for subscripts.
(   mathastext:   ) To keep LaTeX defaults, use option
`defaultmathsizes'.

Package mathastext Info: Loading is complete.  You can now use
\Mathastext to
```

```
(mathastext)             modify the normal and bold math versions.  Use
it
(mathastext)             with optional argument or use \MTDeclareVersion
to
(mathastext)             declare additional math versions.
) (c:/texlive/2023/texmf-dist/tex/latex/relsize/relsize.sty
Package: relsize 2013/03/29 ver 4.1
) (c:/texlive/2023/texmf-dist/tex/latex/ragged2e/ragged2e.sty
Package: ragged2e 2023/06/22 v3.6 ragged2e Package
\CenteringLeftskip=\skip56
\RaggedLeftLeftskip=\skip57
\RaggedRightLeftskip=\skip58
\CenteringRightskip=\skip59
\RaggedLeftRightskip=\skip60
\RaggedRightRightskip=\skip61
\CenteringParfillskip=\skip62
\RaggedLeftParfillskip=\skip63
\RaggedRightParfillskip=\skip64
\JustifyingParfillskip=\skip65
\CenteringParindent=\skip66
\RaggedLeftParindent=\skip67
\RaggedRightParindent=\skip68
\JustifyingParindent=\skip69
) (c:/texlive/2023/texmf-dist/tex/latex/xcolor/xcolor.sty
Package: xcolor 2023/11/15 v3.01 LaTeX color extensions (UK)
(c:/texlive/2023/texmf-dist/tex/latex/graphics-cfg/color.cfg
File: color.cfg 2016/01/02 v1.6 sample color configuration
)
Package xcolor Info: Driver file: pdftex.def on input line 274.
(c:/texlive/2023/texmf-dist/tex/latex/graphics-def/pdftex.def
File: pdftex.def 2022/09/22 v1.2b Graphics/color driver for pdftex
) (c:/texlive/2023/texmf-dist/tex/latex/graphics/mathcolor.ltx)
Package xcolor Info: Model `cmy' substituted by `cmy0' on input line
1350.
Package xcolor Info: Model `hsb' substituted by `rgb' on input line 1354.
Package xcolor Info: Model `RGB' extended on input line 1366.
Package xcolor Info: Model `HTML' substituted by `rgb' on input line
1368.
Package xcolor Info: Model `Hsb' substituted by `hsb' on input line 1369.
Package xcolor Info: Model `tHsb' substituted by `hsb' on input line
1370.
Package xcolor Info: Model `HSB' substituted by `hsb' on input line 1371.
Package xcolor Info: Model `Gray' substituted by `gray' on input line
1372.
Package xcolor Info: Model `wave' substituted by `hsb' on input line
1373.
) (c:/texlive/2023/texmf-dist/tex/latex/colortbl/colortbl.sty
Package: colortbl 2024/02/20 v1.0g Color table columns (DPC)
(c:/texlive/2023/texmf-dist/tex/latex/tools/array.sty
Package: array 2023/10/16 v2.5g Tabular extension package (FMi)
\col@sep=\dimen143
\ar@mcellbox=\box54
\extrarowheight=\dimen144
\NC@list=\toks31
```

```
\extratabsurround=\skip70
\backup@length=\skip71
\ar@cellbox=\box55
)
\everycr=\toks32
\minrowclearance=\skip72
\rownum=\count276
) (c:/texlive/2023/texmf-dist/tex/latex/graphics/graphicx.sty
Package: graphicx 2021/09/16 v1.2d Enhanced LaTeX Graphics (DPC,SPQR)
(c:/texlive/2023/texmf-dist/tex/latex/graphics/graphics.sty
Package: graphics 2022/03/10 v1.4e Standard LaTeX Graphics (DPC,SPQR)
(c:/texlive/2023/texmf-dist/tex/latex/graphics/trig.sty
Package: trig 2021/08/11 v1.11 sin cos tan (DPC)
) (c:/texlive/2023/texmf-dist/tex/latex/graphics-cfg/graphics.cfg
File: graphics.cfg 2016/06/04 v1.11 sample graphics configuration
)
Package graphics Info: Driver file: pdftex.def on input line 107.
)
\Gin@req@height=\dimen145
\Gin@req@width=\dimen146
) (c:/texlive/2023/texmf-dist/tex/latex/xpatch/xpatch.sty
(c:/texlive/2023/texm
f-dist/tex/latex/l3kernel/expl3.sty
Package: expl3 2024-02-20 L3 programming layer (loader)
(c:/texlive/2023/texmf-dist/tex/latex/l3backend/l3backend-pdftex.def
File: l3backend-pdftex.def 2024-02-20 L3 backend support: PDF output
(pdfTeX)
\l__color_backend_stack_int=\count277
\l__pdf_internal_box=\box56
))
Package: xpatch 2020/03/25 v0.3a Extending etoolbox patching commands
(c:/texlive/2023/texmf-dist/tex/latex/l3packages/xparse/xparse.sty
Package: xparse 2024-02-18 L3 Experimental document command parser
)) (c:/texlive/2023/texmf-dist/tex/latex/environ/environ.sty
Package: environ 2014/05/04 v0.3 A new way to define environments
(c:/texlive/2023/texmf-dist/tex/latex/trimspaces/trimspaces.sty
Package: trimspaces 2009/09/17 v1.1 Trim spaces around a token list
)
\@envbody=\toks33
) (c:/texlive/2023/texmf-dist/tex/latex/lastpage/lastpage.sty
Package: lastpage 2023/10/14 v2.0e lastpage: 2.09 or 2e? (HMM)
(c:/texlive/2023/texmf-dist/tex/latex/lastpage/lastpage2e.sty
Package: lastpage2e 2023/10/14 v2.0e Decide which 2e lastpage version to
use (H
MM)
(c:/texlive/2023/texmf-dist/tex/latex/lastpage/lastpagemodern.sty
Package: lastpagemodern 2023-10-14 v2.0e Refers to last page's name (HMM;
JPG)
\c@lastpagecount=\count278
)
)) (c:/texlive/2023/texmf-dist/tex/latex/graphics/rotating.sty
Package: rotating 2016/08/11 v2.16d rotated objects in LaTeX
\c@r@tfl@t=\count279
\rotFPtop=\skip73
```

```
\rotFPbot=\skip74
\rot@float@box=\box57
\rot@mess@toks=\toks34
) (c:/texlive/2023/texmf-dist/tex/latex/graphics/lscape.sty
Package: lscape 2020/05/28 v3.02 Landscape Pages (DPC)
) (c:/texlive/2023/texmf-dist/tex/latex/tools/afterpage.sty
Package: afterpage 2023/07/04 v1.08 After-Page Package (DPC)
\AP@output=\toks35
\AP@partial=\box58
\AP@footins=\box59
) (c:/texlive/2023/texmf-dist/tex/latex/textpos/textpos.sty
Package: textpos 2022/07/23 v1.10.1
Package textpos Info: choosing support for LaTeX3 on input line 60.
\TP@textbox=\box60
\TP@holdbox=\box61
\TPHorizModule=\dimen147
\TPVertModule=\dimen148
\TP@margin=\dimen149
\TP@absmargin=\dimen150
Grid set 16 x 16 = 37.34424pt x 52.81541pt
\TPboxrulesize=\dimen151
\TP@ox=\dimen152
\TP@oy=\dimen153
\TP@tbargs=\toks36
TextBlockOrigin set to 0pt x 0pt
) (c:/texlive/2023/texmf-dist/tex/latex/url/url.sty
\Urlmuskip=\muskip19
Package: url 2013/09/16  ver 3.4  Verb mode for urls, etc.
) (c:/texlive/2023/texmf-dist/tex/latex/newfloat/newfloat.sty
Package: newfloat 2023/10/01 v1.2 Defining new floating environments (AR)
Package newfloat Info: `rotating' package detected.
) (c:/texlive/2023/texmf-dist/tex/latex/mdframed/mdframed.sty
Package: mdframed 2013/07/01 1.9b: mdframed
(c:/texlive/2023/texmf-dist/tex/latex/kvoptions/kvoptions.sty
Package: kvoptions 2022-06-15 v3.15 Key value format for package options
(HO)
(c:/texlive/2023/texmf-dist/tex/generic/ltxcmds/ltxcmds.sty
Package: ltxcmds 2023-12-04 v1.26 LaTeX kernel commands for general use
(HO)
) (c:/texlive/2023/texmf-dist/tex/latex/kvsetkeys/kvsetkeys.sty
Package: kvsetkeys 2022-10-05 v1.19 Key value parser (HO)
)) (c:/texlive/2023/texmf-dist/tex/latex/zref/zref-abspage.sty
Package: zref-abspage 2023-09-14 v2.35 Module abspage for zref (HO)
(c:/texlive/2023/texmf-dist/tex/latex/zref/zref-base.sty
Package: zref-base 2023-09-14 v2.35 Module base for zref (HO)
(c:/texlive/2023/texmf-dist/tex/generic/infwarerr/infwarerr.sty
Package: infwarerr 2019/12/03 v1.5 Providing info/warning/error messages
(HO)
) (c:/texlive/2023/texmf-dist/tex/generic/kvdefinekeys/kvdefinekeys.sty
Package: kvdefinekeys 2019-12-19 v1.6 Define keys (HO)
) (c:/texlive/2023/texmf-dist/tex/generic/pdftexcmds/pdftexcmds.sty
Package: pdftexcmds 2020-06-27 v0.33 Utility functions of pdfTeX for
LuaTeX (HO
)
```

```
Package pdftexcmds Info: \pdf@primitive is available.
Package pdftexcmds Info: \pdf@ifprimitive is available.
Package pdftexcmds Info: \pdfdraftmode found.
) (c:/texlive/2023/texmf-dist/tex/generic/etexcmds/etexcmds.sty
Package: etexcmds 2019/12/15 v1.7 Avoid name clashes with e-TeX commands
(HO)
) (c:/texlive/2023/texmf-dist/tex/latex/auxhook/auxhook.sty
Package: auxhook 2019-12-17 v1.6 Hooks for auxiliary files (HO)
)
Package zref Info: New property list: main on input line 767.
Package zref Info: New property: default on input line 768.
Package zref Info: New property: page on input line 769.
)
\c@abspage=\count280
Package zref Info: New property: abspage on input line 67.
) (c:/texlive/2023/texmf-dist/tex/latex/needspace/needspace.sty
Package: needspace 2010/09/12 v1.3d reserve vertical space
)
\mdf@templength=\skip75
\c@mdf@globalstyle@cnt=\count281
\mdf@skipabove@length=\skip76
\mdf@skipbelow@length=\skip77
\mdf@leftmargin@length=\skip78
\mdf@rightmargin@length=\skip79
\mdf@innerleftmargin@length=\skip80
\mdf@innerrightmargin@length=\skip81
\mdf@innertopmargin@length=\skip82
\mdf@innerbottommargin@length=\skip83
\mdf@splittopskip@length=\skip84
\mdf@splitbottomskip@length=\skip85
\mdf@outermargin@length=\skip86
\mdf@innermargin@length=\skip87
\mdf@linewidth@length=\skip88
\mdf@innerlinewidth@length=\skip89
\mdf@middlelinewidth@length=\skip90
\mdf@outerlinewidth@length=\skip91
\mdf@roundcorner@length=\skip92
\mdf@footenotedistance@length=\skip93
\mdf@userdefinedwidth@length=\skip94
\mdf@needspace@length=\skip95
\mdf@frametitleaboveskip@length=\skip96
\mdf@frametitlebelowskip@length=\skip97
\mdf@frametitlerulewidth@length=\skip98
\mdf@frametitleleftmargin@length=\skip99
\mdf@frametitlerightmargin@length=\skip100
\mdf@shadowsize@length=\skip101
\mdf@extratopheight@length=\skip102
\mdf@subtitleabovelinewidth@length=\skip103
\mdf@subtitlebelowlinewidth@length=\skip104
\mdf@subtitleaboveskip@length=\skip105
\mdf@subtitlebelowskip@length=\skip106
\mdf@subtitleinneraboveskip@length=\skip107
\mdf@subtitleinnerbelowskip@length=\skip108
\mdf@subsubtitleabovelinewidth@length=\skip109
```

```
\mdf@subsubtitlebelowlinewidth@length=\skip110
\mdf@subsubtitleaboveskip@length=\skip111
\mdf@subsubtitlebelowskip@length=\skip112
\mdf@subsubtitleinneraboveskip@length=\skip113
\mdf@subsubtitleinnerbelowskip@length=\skip114
(c:/texlive/2023/texmf-dist/tex/latex/mdframed/md-frame-0.mdf
File: md-frame-0.mdf 2013/07/01\ 1.9b: md-frame-0
)
\mdf@frametitlebox=\box62
\mdf@footnotebox=\box63
\mdf@splitbox@one=\box64
\mdf@splitbox@two=\box65
\mdf@splitbox@save=\box66
\mdfsplitboxwidth=\skip115
\mdfsplitboxtotalwidth=\skip116
\mdfsplitboxheight=\skip117
\mdfsplitboxdepth=\skip118
\mdfsplitboxtotalheight=\skip119
\mdfframetitleboxwidth=\skip120
\mdfframetitleboxtotalwidth=\skip121
\mdfframetitleboxheight=\skip122
\mdfframetitleboxdepth=\skip123
\mdfframetitleboxtotalheight=\skip124
\mdffootnoteboxwidth=\skip125
\mdffootnoteboxtotalwidth=\skip126
\mdffootnoteboxheight=\skip127
\mdffootnoteboxdepth=\skip128
\mdffootnoteboxtotalheight=\skip129
\mdftotallinewidth=\skip130
\mdfboundingboxwidth=\skip131
\mdfboundingboxtotalwidth=\skip132
\mdfboundingboxheight=\skip133
\mdfboundingboxdepth=\skip134
\mdfboundingboxtotalheight=\skip135
\mdf@freevspace@length=\skip136
\mdf@horizontalwidthofbox@length=\skip137
\mdf@verticalmarginwhole@length=\skip138
\mdf@horizontalspaceofbox=\skip139
\mdfsubtitleheight=\skip140
\mdfsubsubtitleheight=\skip141
\c@mdfcountframes=\count282

****** mdframed patching \endmdf@trivlist

****** -- success******

\mdf@envdepth=\count283
\c@mdf@env@i=\count284
\c@mdf@env@ii=\count285
\c@mdf@zref@counter=\count286
Package zref Info: New property: mdf@pagevalue on input line 895.
) (c:/texlive/2023/texmf-dist/tex/latex/titlesec/titlesec.sty
Package: titlesec 2023/10/27 v2.16 Sectioning titles
\ttl@box=\box67
```

```
\beforetitleunit=\skip142
\aftertitleunit=\skip143
\ttl@plus=\dimen154
\ttl@minus=\dimen155
\ttl@toksa=\toks37
\titlewidth=\dimen156
\titlewidthlast=\dimen157
\titlewidthfirst=\dimen158
) (c:/texlive/2023/texmf-dist/tex/latex/koma-script/scrextend.sty
Package: scrextend 2023/07/07 v3.41 KOMA-Script package (extend other
classes w
ith features of KOMA-Script classes)
(c:/texlive/2023/texmf-dist/tex/latex/koma-script/scrkbase.sty
Package: scrkbase 2023/07/07 v3.41 KOMA-Script package (KOMA-Script-
dependent b
asics and keyval usage)
(c:/texlive/2023/texmf-dist/tex/latex/koma-script/scrbase.sty
Package: scrbase 2023/07/07 v3.41 KOMA-Script package (KOMA-Script-
independent
basics and keyval usage)
(c:/texlive/2023/texmf-dist/tex/latex/koma-script/scrlfile.sty
Package: scrlfile 2023/07/07 v3.41 KOMA-Script package (file load hooks)
(c:/texlive/2023/texmf-dist/tex/latex/koma-script/scrlfile-hook.sty
Package: scrlfile-hook 2023/07/07 v3.41 KOMA-Script package (using LaTeX
hooks)

(c:/texlive/2023/texmf-dist/tex/latex/koma-script/scrlogo.sty
Package: scrlogo 2023/07/07 v3.41 KOMA-Script package (logo)
)))
Applying: [2021/05/01] Usage of raw or classic option list on input line
252.
Already applied: [0000/00/00] Usage of raw or classic option list on
input line
 368.
))
Package scrextend Info: unexpected definition of `\@makefnmark'.
(scrextend)             Trying to patch it on input line 1762.
Package scrextend Info: patch seems to be successfull on input line 1762.
)

LaTeX Font Warning: Font shape `T1/cmr/m/n' in size <7.5> not available
(Font)              size <7> substituted on input line 72.

(c:/texlive/2023/texmf-dist/tex/latex/tools/calc.sty
Package: calc 2023/07/08 v4.3 Infix arithmetic (KKT,FJ)
\calc@Acount=\count287
\calc@Bcount=\count288
\calc@Adimen=\dimen159
\calc@Bdimen=\dimen160
\calc@Askip=\skip144
\calc@Bskip=\skip145
LaTeX Info: Redefining \setlength on input line 80.
LaTeX Info: Redefining \addtolength on input line 81.
\calc@Ccount=\count289
```

```
\calc@Cskip=\skip146
) (c:/texlive/2023/texmf-dist/tex/latex/geometry/geometry.sty
Package: geometry 2020/01/02 v5.9 Page Geometry
(c:/texlive/2023/texmf-dist/tex/generic/iftex/ifvtex.sty
Package: ifvtex 2019/10/25 v1.7 ifvtex legacy package. Use iftex instead.
)
\Gm@cnth=\count290
\Gm@cntv=\count291
\c@Gm@tempcnt=\count292
\Gm@bindingoffset=\dimen161
\Gm@wd@mp=\dimen162
\Gm@odd@mp=\dimen163
\Gm@even@mp=\dimen164
\Gm@layoutwidth=\dimen165
\Gm@layoutheight=\dimen166
\Gm@layouthoffset=\dimen167
\Gm@layoutvoffset=\dimen168
\Gm@dimlist=\toks38
) (c:/texlive/2023/texmf-dist/tex/latex/preprint/authblk.sty
Package: authblk 2001/02/27 1.3 (PWD)
\affilsep=\skip147
\@affilsep=\skip148
\c@Maxaffil=\count293
\c@authors=\count294
\c@affil=\count295
) (c:/texlive/2023/texmf-dist/tex/latex/footmisc/footmisc.sty
Package: footmisc 2023/07/05 v6.0f a miscellany of footnote facilities
\FN@temptoken=\toks39
\footnotemargin=\dimen169
\@outputbox@depth=\dimen170
Package footmisc Info: Declaring symbol style bringhurst on input line
696.
Package footmisc Info: Declaring symbol style chicago on input line 704.
Package footmisc Info: Declaring symbol style wiley on input line 713.
Package footmisc Info: Declaring symbol style lamport-robust on input
line 724.

Package footmisc Info: Declaring symbol style lamport* on input line 744.
Package footmisc Info: Declaring symbol style lamport*-robust on input
line 765
.
) (c:/texlive/2023/texmf-dist/tex/latex/fancyhdr/fancyhdr.sty
Package: fancyhdr 2022/11/09 v4.1 Extensive control of page headers and
footers

\f@nch@headwidth=\skip149
\f@nch@O@elh=\skip150
\f@nch@O@erh=\skip151
\f@nch@O@olh=\skip152
\f@nch@O@orh=\skip153
\f@nch@O@elf=\skip154
\f@nch@O@erf=\skip155
\f@nch@O@olf=\skip156
\f@nch@O@orf=\skip157
```

```
) (c:/texlive/2023/texmf-dist/tex/generic/alphalph/alphalph.sty
Package: alphalph 2019/12/09 v2.6 Convert numbers to letters (HO)
(c:/texlive/2023/texmf-dist/tex/generic/intcalc/intcalc.sty
Package: intcalc 2019/12/15 v1.3 Expandable calculations with integers
(HO)
))
\c@authorfn=\count296
(c:/texlive/2023/texmf-dist/tex/latex/abstract/abstract.sty
Package: abstract 2009/06/08 v1.2a configurable abstracts
\abstitleskip=\skip158
\absleftindent=\skip159
\absrightindent=\skip160
\absparindent=\skip161
\absparsep=\skip162
)
Package newfloat Info: New float `keypoints' with options
`placement=t!,name=kp
t' on input line 294.
\c@keypoints=\count297
Package newfloat Info: float type `keypoints'=8 on input line 294.
(c:/texlive/2023/texmf-dist/tex/latex/enumitem/enumitem.sty
Package: enumitem 2019/06/20 v3.9 Customized lists
\labelindent=\skip163
\enit@outerparindent=\dimen171
\enit@toks=\toks40
\enit@inbox=\box68
\enit@count@id=\count298
\enitdp@description=\count299
) (c:/texlive/2023/texmf-dist/tex/latex/quoting/quoting.sty
Package: quoting 2014/01/28 v0.1c Consolidated environment for displayed
text
\quo@toppartop=\skip164
) (c:/texlive/2023/texmf-dist/tex/latex/sttools/stfloats.sty
Package: stfloats 2017/03/27 v3.3 Improve float mechanism and
baselineskip sett
ings
\@dblbotnum=\count300
\c@dblbotnumber=\count301
) (c:/texlive/2023/texmf-dist/tex/latex/booktabs/booktabs.sty
Package: booktabs 2020/01/12 v1.61803398 Publication quality tables
\heavyrulewidth=\dimen172
\lightrulewidth=\dimen173
\cmidrulewidth=\dimen174
\belowrulesep=\dimen175
\belowbottomsep=\dimen176
\aboverulesep=\dimen177
\abovetopsep=\dimen178
\cmidrulesep=\dimen179
\cmidrulekern=\dimen180
\defaultaddspace=\dimen181
\@cmidla=\count302
\@cmidlb=\count303
\@aboverulesep=\dimen182
\@belowrulesep=\dimen183
```

```
\@thisruleclass=\count304
\@lastruleclass=\count305
\@thisrulewidth=\dimen184
) (c:/texlive/2023/texmf-dist/tex/latex/tools/tabularx.sty
Package: tabularx 2023/07/08 v2.11c `tabularx' package (DPC)
\TX@col@width=\dimen185
\TX@old@table=\dimen186
\TX@old@col=\dimen187
\TX@target=\dimen188
\TX@delta=\dimen189
\TX@cols=\count306
\TX@ftn=\toks41
)
\enitdp@tablenotes=\count307
(c:/texlive/2023/texmf-dist/tex/latex/caption/caption.sty
Package: caption 2023/08/05 v3.6o Customizing captions (AR)
(c:/texlive/2023/texmf-dist/tex/latex/caption/caption3.sty
Package: caption3 2023/07/31 v2.4d caption3 kernel (AR)
\caption@tempdima=\dimen190
\captionmargin=\dimen191
\caption@leftmargin=\dimen192
\caption@rightmargin=\dimen193
\caption@width=\dimen194
\caption@indent=\dimen195
\caption@parindent=\dimen196
\caption@hangindent=\dimen197
Package caption Info: Standard document class detected.
)
\c@caption@flags=\count308
\c@continuedfloat=\count309
Package caption Info: float package is loaded.
Package caption Info: rotating package is loaded.
Package caption Info: scrextend package is loaded.
\caption@addmargin@hsize=\dimen198
\caption@addmargin@linewidth=\dimen199
) (c:/texlive/2023/texmf-dist/tex/latex/natbib/natbib.sty
Package: natbib 2010/09/13 8.31b (PWD, AO)
\bibhang=\skip165
\bibsep=\skip166
LaTeX Info: Redefining \cite on input line 694.
\c@NAT@ctr=\count310
)) (c:/texlive/2023/texmf-dist/tex/latex/pgf/frontendlayer/tikz.sty
(c:/texlive
/2023/texmf-dist/tex/latex/pgf/basiclayer/pgf.sty (c:/texlive/2023/texmf-
dist/t
ex/latex/pgf/utilities/pgfrcs.sty (c:/texlive/2023/texmf-
dist/tex/generic/pgf/u
tilities/pgfutil-common.tex
\pgfutil@everybye=\toks42
\pgfutil@tempdima=\dimen256
\pgfutil@tempdimb=\dimen257
) (c:/texlive/2023/texmf-dist/tex/generic/pgf/utilities/pgfutil-latex.def
\pgfutil@abb=\box69
```

```
) (c:/texlive/2023/texmf-dist/tex/generic/pgf/utilities/pgfrcs.code.tex
(c:/tex
live/2023/texmf-dist/tex/generic/pgf/pgf.revision.tex)
Package: pgfrcs 2023-01-15 v3.1.10 (3.1.10)
))
Package: pgf 2023-01-15 v3.1.10 (3.1.10)
(c:/texlive/2023/texmf-dist/tex/latex/pgf/basiclayer/pgfcore.sty
(c:/texlive/20
23/texmf-dist/tex/latex/pgf/systemlayer/pgfsys.sty
(c:/texlive/2023/texmf-dist/
tex/generic/pgf/systemlayer/pgfsys.code.tex
Package: pgfsys 2023-01-15 v3.1.10 (3.1.10)
(c:/texlive/2023/texmf-dist/tex/generic/pgf/utilities/pgfkeys.code.tex
\pgfkeys@pathtoks=\toks43
\pgfkeys@temptoks=\toks44

(c:/texlive/2023/texmf-
dist/tex/generic/pgf/utilities/pgfkeyslibraryfiltered.co
de.tex
\pgfkeys@tmptoks=\toks45
))
\pgf@x=\dimen258
\pgf@y=\dimen259
\pgf@xa=\dimen260
\pgf@ya=\dimen261
\pgf@xb=\dimen262
\pgf@yb=\dimen263
\pgf@xc=\dimen264
\pgf@yc=\dimen265
\pgf@xd=\dimen266
\pgf@yd=\dimen267
\w@pgf@writea=\write3
\r@pgf@reada=\read2
\c@pgf@counta=\count311
\c@pgf@countb=\count312
\c@pgf@countc=\count313
\c@pgf@countd=\count314
\t@pgf@toka=\toks46
\t@pgf@tokb=\toks47
\t@pgf@tokc=\toks48
\pgf@sys@id@count=\count315
(c:/texlive/2023/texmf-dist/tex/generic/pgf/systemlayer/pgf.cfg
File: pgf.cfg 2023-01-15 v3.1.10 (3.1.10)
)
Driver file for pgf: pgfsys-pdftex.def
(c:/texlive/2023/texmf-dist/tex/generic/pgf/systemlayer/pgfsys-pdftex.def
File: pgfsys-pdftex.def 2023-01-15 v3.1.10 (3.1.10)
(c:/texlive/2023/texmf-dist/tex/generic/pgf/systemlayer/pgfsys-common-
pdf.def
File: pgfsys-common-pdf.def 2023-01-15 v3.1.10 (3.1.10)
)))
(c:/texlive/2023/texmf-
dist/tex/generic/pgf/systemlayer/pgfsyssoftpath.code.tex
File: pgfsyssoftpath.code.tex 2023-01-15 v3.1.10 (3.1.10)
```

```
\pgfsyssoftpath@smallbuffer@items=\count316
\pgfsyssoftpath@bigbuffer@items=\count317
)
(c:/texlive/2023/texmf-
dist/tex/generic/pgf/systemlayer/pgfsysprotocol.code.tex
File: pgfsysprotocol.code.tex 2023-01-15 v3.1.10 (3.1.10)
)) (c:/texlive/2023/texmf-
dist/tex/generic/pgf/basiclayer/pgfcore.code.tex
Package: pgfcore 2023-01-15 v3.1.10 (3.1.10)
(c:/texlive/2023/texmf-dist/tex/generic/pgf/math/pgfmath.code.tex
(c:/texlive/2
023/texmf-dist/tex/generic/pgf/math/pgfmathutil.code.tex)
(c:/texlive/2023/texm
f-dist/tex/generic/pgf/math/pgfmathparser.code.tex
\pgfmath@dimen=\dimen268
\pgfmath@count=\count318
\pgfmath@box=\box70
\pgfmath@toks=\toks49
\pgfmath@stack@operand=\toks50
\pgfmath@stack@operation=\toks51
) (c:/texlive/2023/texmf-
dist/tex/generic/pgf/math/pgfmathfunctions.code.tex)
(c:/texlive/2023/texmf-
dist/tex/generic/pgf/math/pgfmathfunctions.basic.code.te
x)
(c:/texlive/2023/texmf-
dist/tex/generic/pgf/math/pgfmathfunctions.trigonometric
.code.tex)
(c:/texlive/2023/texmf-
dist/tex/generic/pgf/math/pgfmathfunctions.random.code.t
ex)
(c:/texlive/2023/texmf-
dist/tex/generic/pgf/math/pgfmathfunctions.comparison.co
de.tex)
(c:/texlive/2023/texmf-
dist/tex/generic/pgf/math/pgfmathfunctions.base.code.tex
)
(c:/texlive/2023/texmf-
dist/tex/generic/pgf/math/pgfmathfunctions.round.code.te
x)
(c:/texlive/2023/texmf-
dist/tex/generic/pgf/math/pgfmathfunctions.misc.code.tex
)
(c:/texlive/2023/texmf-
dist/tex/generic/pgf/math/pgfmathfunctions.integerarithm
etics.code.tex) (c:/texlive/2023/texmf-
dist/tex/generic/pgf/math/pgfmathcalc.co
de.tex) (c:/texlive/2023/texmf-
dist/tex/generic/pgf/math/pgfmathfloat.code.tex
\c@pgfmathroundto@lastzeros=\count319
)) (c:/texlive/2023/texmf-dist/tex/generic/pgf/math/pgfint.code.tex)
(c:/texliv
e/2023/texmf-dist/tex/generic/pgf/basiclayer/pgfcorepoints.code.tex
File: pgfcorepoints.code.tex 2023-01-15 v3.1.10 (3.1.10)
```

```
\pgf@picminx=\dimen269
\pgf@picmaxx=\dimen270
\pgf@picminy=\dimen271
\pgf@picmaxy=\dimen272
\pgf@pathminx=\dimen273
\pgf@pathmaxx=\dimen274
\pgf@pathminy=\dimen275
\pgf@pathmaxy=\dimen276
\pgf@xx=\dimen277
\pgf@xy=\dimen278
\pgf@yx=\dimen279
\pgf@yy=\dimen280
\pgf@zx=\dimen281
\pgf@zy=\dimen282
)
(c:/texlive/2023/texmf-
dist/tex/generic/pgf/basiclayer/pgfcorepathconstruct.cod
e.tex
File: pgfcorepathconstruct.code.tex 2023-01-15 v3.1.10 (3.1.10)
\pgf@path@lastx=\dimen283
\pgf@path@lasty=\dimen284
)
(c:/texlive/2023/texmf-
dist/tex/generic/pgf/basiclayer/pgfcorepathusage.code.te
x
File: pgfcorepathusage.code.tex 2023-01-15 v3.1.10 (3.1.10)
\pgf@shorten@end@additional=\dimen285
\pgf@shorten@start@additional=\dimen286
) (c:/texlive/2023/texmf-
dist/tex/generic/pgf/basiclayer/pgfcorescopes.code.tex
File: pgfcorescopes.code.tex 2023-01-15 v3.1.10 (3.1.10)
\pgfpic=\box71
\pgf@hbox=\box72
\pgf@layerbox@main=\box73
\pgf@picture@serial@count=\count320
)
(c:/texlive/2023/texmf-
dist/tex/generic/pgf/basiclayer/pgfcoregraphicstate.code
.tex
File: pgfcoregraphicstate.code.tex 2023-01-15 v3.1.10 (3.1.10)
\pgflinewidth=\dimen287
)
(c:/texlive/2023/texmf-
dist/tex/generic/pgf/basiclayer/pgfcoretransformations.c
ode.tex
File: pgfcoretransformations.code.tex 2023-01-15 v3.1.10 (3.1.10)
\pgf@pt@x=\dimen288
\pgf@pt@y=\dimen289
\pgf@pt@temp=\dimen290
) (c:/texlive/2023/texmf-
dist/tex/generic/pgf/basiclayer/pgfcorequick.code.tex
File: pgfcorequick.code.tex 2023-01-15 v3.1.10 (3.1.10)
) (c:/texlive/2023/texmf-
dist/tex/generic/pgf/basiclayer/pgfcoreobjects.code.te
```

```
x
File: pgfcoreobjects.code.tex 2023-01-15 v3.1.10 (3.1.10)
)
(c:/texlive/2023/texmf-
dist/tex/generic/pgf/basiclayer/pgfcorepathprocessing.co
de.tex
File: pgfcorepathprocessing.code.tex 2023-01-15 v3.1.10 (3.1.10)
) (c:/texlive/2023/texmf-
dist/tex/generic/pgf/basiclayer/pgfcorearrows.code.tex
File: pgfcorearrows.code.tex 2023-01-15 v3.1.10 (3.1.10)
\pgfarrowsep=\dimen291
) (c:/texlive/2023/texmf-
dist/tex/generic/pgf/basiclayer/pgfcoreshade.code.tex
File: pgfcoreshade.code.tex 2023-01-15 v3.1.10 (3.1.10)
\pgf@max=\dimen292
\pgf@sys@shading@range@num=\count321
\pgf@shadingcount=\count322
) (c:/texlive/2023/texmf-
dist/tex/generic/pgf/basiclayer/pgfcoreimage.code.tex
File: pgfcoreimage.code.tex 2023-01-15 v3.1.10 (3.1.10)
)
(c:/texlive/2023/texmf-
dist/tex/generic/pgf/basiclayer/pgfcoreexternal.code.tex
File: pgfcoreexternal.code.tex 2023-01-15 v3.1.10 (3.1.10)
\pgfexternal@startupbox=\box74
) (c:/texlive/2023/texmf-
dist/tex/generic/pgf/basiclayer/pgfcorelayers.code.tex
File: pgfcorelayers.code.tex 2023-01-15 v3.1.10 (3.1.10)
)
(c:/texlive/2023/texmf-
dist/tex/generic/pgf/basiclayer/pgfcoretransparency.code
.tex
File: pgfcoretransparency.code.tex 2023-01-15 v3.1.10 (3.1.10)
)
(c:/texlive/2023/texmf-
dist/tex/generic/pgf/basiclayer/pgfcorepatterns.code.tex
File: pgfcorepatterns.code.tex 2023-01-15 v3.1.10 (3.1.10)
) (c:/texlive/2023/texmf-
dist/tex/generic/pgf/basiclayer/pgfcorerdf.code.tex
File: pgfcorerdf.code.tex 2023-01-15 v3.1.10 (3.1.10)
))) (c:/texlive/2023/texmf-
dist/tex/generic/pgf/modules/pgfmoduleshapes.code.te
x
File: pgfmoduleshapes.code.tex 2023-01-15 v3.1.10 (3.1.10)
\pgfnodeparttextbox=\box75
) (c:/texlive/2023/texmf-
dist/tex/generic/pgf/modules/pgfmoduleplot.code.tex
File: pgfmoduleplot.code.tex 2023-01-15 v3.1.10 (3.1.10)
)
(c:/texlive/2023/texmf-dist/tex/latex/pgf/compatibility/pgfcomp-version-
0-65.st
y
Package: pgfcomp-version-0-65 2023-01-15 v3.1.10 (3.1.10)
\pgf@nodesepstart=\dimen293
```

```
\pgf@nodesepend=\dimen294
)
(c:/texlive/2023/texmf-dist/tex/latex/pgf/compatibility/pgfcomp-version-
1-18.st
y
Package: pgfcomp-version-1-18 2023-01-15 v3.1.10 (3.1.10)
)) (c:/texlive/2023/texmf-dist/tex/latex/pgf/utilities/pgffor.sty
(c:/texlive/2
023/texmf-dist/tex/latex/pgf/utilities/pgfkeys.sty
(c:/texlive/2023/texmf-dist/
tex/generic/pgf/utilities/pgfkeys.code.tex)) (c:/texlive/2023/texmf-
dist/tex/la
tex/pgf/math/pgfmath.sty (c:/texlive/2023/texmf-
dist/tex/generic/pgf/math/pgfma
th.code.tex)) (c:/texlive/2023/texmf-
dist/tex/generic/pgf/utilities/pgffor.code
.tex
Package: pgffor 2023-01-15 v3.1.10 (3.1.10)
\pgffor@iter=\dimen295
\pgffor@skip=\dimen296
\pgffor@stack=\toks52
\pgffor@toks=\toks53
)) (c:/texlive/2023/texmf-
dist/tex/generic/pgf/frontendlayer/tikz/tikz.code.tex
Package: tikz 2023-01-15 v3.1.10 (3.1.10)

(c:/texlive/2023/texmf-
dist/tex/generic/pgf/libraries/pgflibraryplothandlers.co
de.tex
File: pgflibraryplothandlers.code.tex 2023-01-15 v3.1.10 (3.1.10)
\pgf@plot@mark@count=\count323
\pgfplotmarksize=\dimen297
)
\tikz@lastx=\dimen298
\tikz@lasty=\dimen299
\tikz@lastxsaved=\dimen300
\tikz@lastysaved=\dimen301
\tikz@lastmovetox=\dimen302
\tikz@lastmovetoy=\dimen303
\tikzleveldistance=\dimen304
\tikzsiblingdistance=\dimen305
\tikz@figbox=\box76
\tikz@figbox@bg=\box77
\tikz@tempbox=\box78
\tikz@tempbox@bg=\box79
\tikztreelevel=\count324
\tikznumberofchildren=\count325
\tikznumberofcurrentchild=\count326
\tikz@fig@count=\count327
(c:/texlive/2023/texmf-
dist/tex/generic/pgf/modules/pgfmodulematrix.code.tex
File: pgfmodulematrix.code.tex 2023-01-15 v3.1.10 (3.1.10)
\pgfmatrixcurrentrow=\count328
\pgfmatrixcurrentcolumn=\count329
```

```
\pgf@matrix@numberofcolumns=\count330
)
\tikz@expandcount=\count331

(c:/texlive/2023/texmf-
dist/tex/generic/pgf/frontendlayer/tikz/libraries/tikzli
brarytopaths.code.tex
File: tikzlibrarytopaths.code.tex 2023-01-15 v3.1.10 (3.1.10)
))) (c:/texlive/2023/texmf-dist/tex/latex/amsmath/amsmath.sty
Package: amsmath 2023/05/13 v2.17o AMS math features
\@mathmargin=\skip167
For additional information on amsmath, use the `?' option.
(c:/texlive/2023/texmf-dist/tex/latex/amsmath/amstext.sty
Package: amstext 2021/08/26 v2.01 AMS text
(c:/texlive/2023/texmf-dist/tex/latex/amsmath/amsgen.sty
File: amsgen.sty 1999/11/30 v2.0 generic functions
\@emptytoks=\toks54
\ex@=\dimen306
)) (c:/texlive/2023/texmf-dist/tex/latex/amsmath/amsbsy.sty
Package: amsbsy 1999/11/29 v1.2d Bold Symbols
\pmbraise@=\dimen307
) (c:/texlive/2023/texmf-dist/tex/latex/amsmath/amsopn.sty
Package: amsopn 2022/04/08 v2.04 operator names
)
\inf@bad=\count332
LaTeX Info: Redefining \frac on input line 234.
\uproot@=\count333
\leftroot@=\count334
LaTeX Info: Redefining \overline on input line 399.
LaTeX Info: Redefining \colon on input line 410.
\classnum@=\count335
\DOTSCASE@=\count336
LaTeX Info: Redefining \ldots on input line 496.
LaTeX Info: Redefining \dots on input line 499.
LaTeX Info: Redefining \cdots on input line 620.
\Mathstrutbox@=\box80
\strutbox@=\box81
LaTeX Info: Redefining \big on input line 722.
LaTeX Info: Redefining \Big on input line 723.
LaTeX Info: Redefining \bigg on input line 724.
LaTeX Info: Redefining \Bigg on input line 725.
\big@size=\dimen308
LaTeX Font Info:    Redeclaring font encoding OML on input line 743.
LaTeX Font Info:    Redeclaring font encoding OMS on input line 744.
\macc@depth=\count337
LaTeX Info: Redefining \bmod on input line 905.
LaTeX Info: Redefining \pmod on input line 910.
LaTeX Info: Redefining \smash on input line 940.
LaTeX Info: Redefining \relbar on input line 970.
LaTeX Info: Redefining \Relbar on input line 971.
\c@MaxMatrixCols=\count338
\dotsspace@=\muskip20
\c@parentequation=\count339
\dspbrk@lvl=\count340
```

```
\tag@help=\toks55
\row@=\count341
\column@=\count342
\maxfields@=\count343
\andhelp@=\toks56
\eqnshift@=\dimen309
\alignsep@=\dimen310
\tagshift@=\dimen311
\tagwidth@=\dimen312
\totwidth@=\dimen313
\lineht@=\dimen314
\@envbody=\toks57
\multlinegap=\skip168
\multlinetaggap=\skip169
\mathdisplay@stack=\toks58
LaTeX Info: Redefining \[ on input line 2953.
LaTeX Info: Redefining \] on input line 2954.
) (c:/texlive/2023/texmf-dist/tex/latex/orcidlink/orcidlink.sty
Package: orcidlink 2023/12/30 v1.0.5 Linked ORCiD logo macro package
(c:/texlive/2023/texmf-dist/tex/latex/hyperref/hyperref.sty
Package: hyperref 2024-01-20 v7.01h Hypertext links for LaTeX
(c:/texlive/2023/texmf-dist/tex/generic/pdfescape/pdfescape.sty
Package: pdfescape 2019/12/09 v1.15 Implements pdfTeX's escape features
(HO)
) (c:/texlive/2023/texmf-dist/tex/latex/hycolor/hycolor.sty
Package: hycolor 2020-01-27 v1.10 Color options for hyperref/bookmark
(HO)
) (c:/texlive/2023/texmf-dist/tex/latex/hyperref/nameref.sty
Package: nameref 2023-11-26 v2.56 Cross-referencing by name of section
(c:/texlive/2023/texmf-dist/tex/latex/refcount/refcount.sty
Package: refcount 2019/12/15 v3.6 Data extraction from label references
(HO)
) (c:/texlive/2023/texmf-
dist/tex/generic/gettitlestring/gettitlestring.sty
Package: gettitlestring 2019/12/15 v1.6 Cleanup title references (HO)
)
\c@section@level=\count344
)
\@linkdim=\dimen315
\Hy@linkcounter=\count345
\Hy@pagecounter=\count346
(c:/texlive/2023/texmf-dist/tex/latex/hyperref/pd1enc.def
File: pd1enc.def 2024-01-20 v7.01h Hyperref: PDFDocEncoding definition
(HO)
Now handling font encoding PD1 ...
... no UTF-8 mapping file for font encoding PD1
)
\Hy@SavedSpaceFactor=\count347
(c:/texlive/2023/texmf-dist/tex/latex/hyperref/puenc.def
File: puenc.def 2024-01-20 v7.01h Hyperref: PDF Unicode definition (HO)
Now handling font encoding PU ...
... no UTF-8 mapping file for font encoding PU
)
Package hyperref Info: Hyper figures OFF on input line 4179.
```

```
Package hyperref Info: Link nesting OFF on input line 4184.
Package hyperref Info: Hyper index ON on input line 4187.
Package hyperref Info: Plain pages OFF on input line 4194.
Package hyperref Info: Backreferencing OFF on input line 4199.
Package hyperref Info: Implicit mode ON; LaTeX internals redefined.
Package hyperref Info: Bookmarks ON on input line 4446.
\c@Hy@tempcnt=\count348
LaTeX Info: Redefining \url on input line 4784.
\XeTeXLinkMargin=\dimen316
(c:/texlive/2023/texmf-dist/tex/generic/bitset/bitset.sty
Package: bitset 2019/12/09 v1.3 Handle bit-vector datatype (HO)
(c:/texlive/2023/texmf-dist/tex/generic/bigintcalc/bigintcalc.sty
Package: bigintcalc 2019/12/15 v1.5 Expandable calculations on big
integers (HO
)
))
\Fld@menulength=\count349
\Field@Width=\dimen317
\Fld@charsize=\dimen318
Package hyperref Info: Hyper figures OFF on input line 6063.
Package hyperref Info: Link nesting OFF on input line 6068.
Package hyperref Info: Hyper index ON on input line 6071.
Package hyperref Info: backreferencing OFF on input line 6078.
Package hyperref Info: Link coloring OFF on input line 6083.
Package hyperref Info: Link coloring with OCG OFF on input line 6088.
Package hyperref Info: PDF/A mode OFF on input line 6093.
(c:/texlive/2023/texmf-dist/tex/latex/base/atbegshi-ltx.sty
Package: atbegshi-ltx 2021/01/10 v1.0c Emulation of the original atbegshi
package with kernel methods
)
\Hy@abspage=\count350
\c@Item=\count351
\c@Hfootnote=\count352
)
Package hyperref Info: Driver (autodetected): hpdftex.
(c:/texlive/2023/texmf-dist/tex/latex/hyperref/hpdftex.def
File: hpdftex.def 2024-01-20 v7.01h Hyperref driver for pdfTeX
(c:/texlive/2023/texmf-dist/tex/latex/base/atveryend-ltx.sty
Package: atveryend-ltx 2020/08/19 v1.0a Emulation of the original
atveryend pac
kage
with kernel methods
)
\HyAnn@Count=\count353
\Fld@listcount=\count354
\c@bookmark@seq@number=\count355
(c:/texlive/2023/texmf-dist/tex/latex/rerunfilecheck/rerunfilecheck.sty
Package: rerunfilecheck 2022-07-10 v1.10 Rerun checks for auxiliary files
(HO)
(c:/texlive/2023/texmf-dist/tex/generic/uniquecounter/uniquecounter.sty
Package: uniquecounter 2019/12/15 v1.4 Provide unlimited unique counter
(HO)
)
```

```
Package uniquecounter Info: New unique counter `rerunfilecheck' on input
line 2
85.
)
\Hy@SectionHShift=\skip170
)
(c:/texlive/2023/texmf-
dist/tex/generic/pgf/frontendlayer/tikz/libraries/tikzli
brarysvg.path.code.tex
File: tikzlibrarysvg.path.code.tex 2023-01-15 v3.1.10 (3.1.10)

(c:/texlive/2023/texmf-
dist/tex/generic/pgf/libraries/pgflibrarysvg.path.code.t
ex
File: pgflibrarysvg.path.code.tex 2023-01-15 v3.1.10 (3.1.10)
(c:/texlive/2023/texmf-
dist/tex/generic/pgf/modules/pgfmoduleparser.code.tex
File: pgfmoduleparser.code.tex 2023-01-15 v3.1.10 (3.1.10)
\pgfparserdef@arg@count=\count356
)
\pgf@lib@svg@last@x=\dimen319
\pgf@lib@svg@last@y=\dimen320
\pgf@lib@svg@last@c@x=\dimen321
\pgf@lib@svg@last@c@y=\dimen322
\pgf@lib@svg@count=\count357
\pgf@lib@svg@max@num=\count358
))
\@curXheight=\skip171
) (c:/texlive/2023/texmf-dist/tex/latex/siunitx/siunitx.sty
Package: siunitx 2024-02-15 v3.3.12 A comprehensive (SI) units package
\l__siunitx_number_uncert_offset_int=\count359
\l__siunitx_number_exponent_fixed_int=\count360
\l__siunitx_number_min_decimal_int=\count361
\l__siunitx_number_min_integer_int=\count362
\l__siunitx_number_round_precision_int=\count363
\l__siunitx_number_lower_threshold_int=\count364
\l__siunitx_number_upper_threshold_int=\count365
\l__siunitx_number_group_first_int=\count366
\l__siunitx_number_group_size_int=\count367
\l__siunitx_number_group_minimum_int=\count368
\l__siunitx_angle_tmp_dim=\dimen323
\l__siunitx_angle_marker_box=\box82
\l__siunitx_angle_unit_box=\box83
\l__siunitx_compound_count_int=\count369
(c:/texlive/2023/texmf-dist/tex/latex/translations/translations.sty
Package: translations 2022/02/05 v1.12 internationalization of LaTeX2e
packages
 (CN)
)
\l__siunitx_table_tmp_box=\box84
\l__siunitx_table_tmp_dim=\dimen324
\l__siunitx_table_column_width_dim=\dimen325
\l__siunitx_table_integer_box=\box85
\l__siunitx_table_decimal_box=\box86
```

```
\l__siunitx_table_uncert_box=\box87
\l__siunitx_table_before_box=\box88
\l__siunitx_table_after_box=\box89
\l__siunitx_table_before_dim=\dimen326
\l__siunitx_table_carry_dim=\dimen327
\l__siunitx_unit_tmp_int=\count370
\l__siunitx_unit_position_int=\count371
\l__siunitx_unit_total_int=\count372
)

! LaTeX Error: Option clash for package hyperref.

See the LaTeX manual or LaTeX Companion for explanation.
Type  H <return>  for immediate help.
 ...

l.61 \begin{document}

The package hyperref has already been loaded with options:
  []
There has now been an attempt to load it with options
  [colorlinks,allcolors=black,urlcolor=blue]
Adding the global options:
  ,colorlinks,allcolors=black,urlcolor=blue
to your \documentclass declaration may fix this.
Try typing  <return>  to proceed.

Package translations Info: No language package found. I am going to use
`englis
h' as default language. on input line 61.
LaTeX Font Info:    Trying to load font information for T1+Merriwthr-OsF
on inp
ut line 61.
(c:/texlive/2023/texmf-dist/tex/latex/merriweather/T1Merriwthr-OsF.fd
File: T1Merriwthr-OsF.fd 2020/08/30 (autoinst) Font definitions for
T1/Merriwth
r-OsF.
)
LaTeX Font Info:    Font shape `T1/Merriwthr-OsF/m/n' will be
(Font)              scaled to size 7.5pt on input line 61.
(./supplementary.aux

LaTeX Warning: Label `tab:cot' multiply defined.

)
\openout1 = `supplementary.aux'.

LaTeX Font Info:    Checking defaults for OML/cmm/m/it on input line 61.
LaTeX Font Info:    ... okay on input line 61.
LaTeX Font Info:    Checking defaults for OMS/cmsy/m/n on input line 61.
LaTeX Font Info:    ... okay on input line 61.
LaTeX Font Info:    Checking defaults for OT1/cmr/m/n on input line 61.
LaTeX Font Info:    ... okay on input line 61.
LaTeX Font Info:    Checking defaults for T1/cmr/m/n on input line 61.
```

```
LaTeX Font Info:    ... okay on input line 61.
LaTeX Font Info:    Checking defaults for TS1/cmr/m/n on input line 61.
LaTeX Font Info:    ... okay on input line 61.
LaTeX Font Info:    Checking defaults for OMX/cmex/m/n on input line 61.
LaTeX Font Info:    ... okay on input line 61.
LaTeX Font Info:    Checking defaults for U/cmr/m/n on input line 61.
LaTeX Font Info:    ... okay on input line 61.
LaTeX Font Info:    Checking defaults for PD1/pdf/m/n on input line 61.
LaTeX Font Info:    ... okay on input line 61.
LaTeX Font Info:    Checking defaults for PU/pdf/m/n on input line 61.
LaTeX Font Info:    ... okay on input line 61.
LaTeX Info: Redefining \microtypecontext on input line 61.
Package microtype Info: Applying patch `item' on input line 61.
Package microtype Info: Applying patch `toc' on input line 61.
Package microtype Info: Applying patch `eqnum' on input line 61.
Package microtype Info: Applying patch `footnote' on input line 61.
Package microtype Info: Applying patch `verbatim' on input line 61.
Package microtype Info: Generating PDF output.
Package microtype Info: Character protrusion enabled (level 2).
Package microtype Info: Using default protrusion set `alltext'.
Package microtype Info: Automatic font expansion enabled (level 2),
(microtype)             stretch: 20, shrink: 20, step: 1, non-selected.
Package microtype Info: Using default expansion set `alltext-nott'.
LaTeX Info: Redefining \showhyphens on input line 61.
Package microtype Info: No adjustment of tracking.
Package microtype Info: No adjustment of interword spacing.
Package microtype Info: No adjustment of character kerning.
Package microtype Info: Loading generic protrusion settings for font
family
(microtype)             `Merriwthr-OsF' (encoding: T1).
(microtype)             For optimal results, create family-specific
settings.
(microtype)             See the microtype manual for details.
LaTeX Font Info:    Redeclaring symbol font `operators' on input line 61.
LaTeX Font Info:    Encoding `OT1' has changed to `T1' for symbol font
(Font)              `operators' in the math version `normal' on input
line 61.
LaTeX Font Info:    Overwriting symbol font `operators' in version
`normal'
(Font)                  OT1/cmr/m/n --> T1/Merriwthr-OsF/m/up on input
line 61.

LaTeX Font Info:    Encoding `OT1' has changed to `T1' for symbol font
(Font)              `operators' in the math version `bold' on input line
61.
LaTeX Font Info:    Overwriting symbol font `operators' in version `bold'
(Font)                  OT1/cmr/bx/n --> T1/Merriwthr-OsF/m/up on input
line 61
.
LaTeX Font Info:    Overwriting symbol font `operators' in version `bold'
(Font)                  T1/Merriwthr-OsF/m/up --> T1/Merriwthr-OsF/b/up
on inpu
t line 61.
LaTeX Font Info:    Redeclaring math alphabet \mathbf on input line 61.
```

```
LaTeX Font Info:    Overwriting math alphabet `\mathbf' in version
`normal'
(Font)              OT1/cmr/bx/n --> T1/Merriwthr-OsF/b/up on input
line 61
.
LaTeX Font Info:    Overwriting math alphabet `\mathbf' in version `bold'
(Font)              OT1/cmr/bx/n --> T1/Merriwthr-OsF/b/up on input
line 61
.
LaTeX Font Info:    Redeclaring math alphabet \mathsf on input line 61.
LaTeX Font Info:    Overwriting math alphabet `\mathsf' in version
`normal'
(Font)              OT1/cmss/m/n --> T1/MerriwthrSans-OsF/m/up on
input lin
e 61.
LaTeX Font Info:    Overwriting math alphabet `\mathsf' in version `bold'
(Font)              OT1/cmss/bx/n --> T1/MerriwthrSans-OsF/m/up on
input li
ne 61.
LaTeX Font Info:    Redeclaring math alphabet \mathit on input line 61.
LaTeX Font Info:    Overwriting math alphabet `\mathit' in version
`normal'
(Font)              OT1/cmr/m/it --> T1/Merriwthr-OsF/m/it on input
line 61
.
LaTeX Font Info:    Overwriting math alphabet `\mathit' in version `bold'
(Font)              OT1/cmr/bx/it --> T1/Merriwthr-OsF/m/it on input
line 6
1.
LaTeX Font Info:    Redeclaring math alphabet \mathtt on input line 61.
LaTeX Font Info:    Overwriting math alphabet `\mathtt' in version
`normal'
(Font)              OT1/cmtt/m/n --> T1/lmtt/m/up on input line 61.
LaTeX Font Info:    Overwriting math alphabet `\mathtt' in version `bold'
(Font)              OT1/cmtt/m/n --> T1/lmtt/m/up on input line 61.
LaTeX Font Info:    Overwriting math alphabet `\mathsf' in version `bold'
(Font)              T1/MerriwthrSans-OsF/m/up --> T1/MerriwthrSans-
OsF/b/up
 on input line 61.
LaTeX Font Info:    Overwriting math alphabet `\mathit' in version `bold'
(Font)              T1/Merriwthr-OsF/m/it --> T1/Merriwthr-OsF/b/it
on inpu
t line 61.
\c@mv@tabular=\count373
\c@mv@boldtabular=\count374
(c:/texlive/2023/texmf-dist/tex/context/base/mkii/supp-pdf.mkii
[Loading MPS to PDF converter (version 2006.09.02).]
\scratchcounter=\count375
\scratchdimen=\dimen328
\scratchbox=\box90
\nofMPsegments=\count376
\nofMParguments=\count377
\everyMPshowfont=\toks59
\MPscratchCnt=\count378
```

```
\MPscratchDim=\dimen329
\MPnumerator=\count379
\makeMPintoPDFobject=\count380
\everyMPtoPDFconversion=\toks60
) (c:/texlive/2023/texmf-dist/tex/latex/epstopdf-pkg/epstopdf-base.sty
Package: epstopdf-base 2020-01-24 v2.11 Base part for package epstopdf
Package epstopdf-base Info: Redefining graphics rule for `.eps' on input line 4
85.
(c:/texlive/2023/texmf-dist/tex/latex/latexconfig/epstopdf-sys.cfg
File: epstopdf-sys.cfg 2010/07/13 v1.3 Configuration of (r)epstopdf for TeX Liv
e
))
Package newfloat Info: `float' package detected.
*geometry* driver: auto-detecting
*geometry* detected driver: pdftex
*geometry* verbose mode - [ preamble ] result:
* driver: pdftex
* paper: a4paper
* layout: <same size as paper>
* layoutoffset:(h,v)=(0.0pt,0.0pt)
* modes: includefoot twoside
* h-part:(L,W,R)=(54.64pt, 488.22787pt, 54.64pt)
* v-part:(T,H,B)=(66.0pt, 745.04684pt, 34.0pt)
* \paperwidth=597.50787pt
* \paperheight=845.04684pt
* \textwidth=488.22787pt
* \textheight=715.04684pt
* \oddsidemargin=-17.62999pt
* \evensidemargin=-17.62999pt
* \topmargin=-47.76999pt
* \headheight=17.5pt
* \headsep=24.0pt
* \topskip=10.0pt
* \footskip=30.0pt
* \marginparwidth=48.0pt
* \marginparsep=10.0pt
* \columnsep=18.0pt
* \skip\footins=22.0pt plus 2.0pt
* \hoffset=0.0pt
* \voffset=0.0pt
* \mag=1000
* \@twocolumntrue
* \@twosidetrue
* \@mparswitchtrue
* \@reversemarginfalse
* (1in=72.27pt=25.4mm, 1cm=28.453pt)

Package caption Info: Begin \AtBeginDocument code.
Package caption Info: hyperref package is loaded.
Package caption Info: End \AtBeginDocument code.
Package hyperref Info: Link coloring OFF on input line 61.
(./supplementary.out) (./supplementary.out)
```

```
\@outlinefile=\write4
\openout4 = `supplementary.out'.


(c:/texlive/2023/texmf-dist/tex/latex/translations/translations-basic-
dictionar
y-english.trsl
File: translations-basic-dictionary-english.trsl (english translation
file `tra
nslations-basic-dictionary')
)
Package translations Info: loading dictionary `translations-basic-
dictionary' f
or `english'. on input line 61.
\@gscitedetails=\box91
\@gscitedetailsheight=\skip172
\@gsheadbox=\box92
\@gsheadboxheight=\skip173
LaTeX Font Info:    Font shape `T1/Merriwthr-OsF/b/n' will be
(Font)              scaled to size 6.5pt on input line 61.
LaTeX Font Info:    Calculating math sizes for size <7.5> on input line
61.

LaTeX Font Warning: Font shape `T1/Merriwthr-OsF/m/up' undefined
(Font)              using `T1/Merriwthr-OsF/m/n' instead on input line
61.

LaTeX Font Info:    Font shape `T1/Merriwthr-OsF/m/up' will be
(Font)              scaled to size 6.24973pt on input line 61.
LaTeX Font Info:    Font shape `T1/Merriwthr-OsF/m/up' will be
(Font)              scaled to size 5.24997pt on input line 61.
LaTeX Font Info:    Trying to load font information for U+eur on input
line 61.

(c:/texlive/2023/texmf-dist/tex/latex/amsfonts/ueur.fd
File: ueur.fd 2013/01/14 v3.01 Euler Roman
) (c:/texlive/2023/texmf-dist/tex/latex/microtype/mt-eur.cfg
File: mt-eur.cfg 2006/07/31 v1.1 microtype config. file: AMS Euler Roman
(RS)
)

LaTeX Font Warning: Font shape `OMS/cmsy/m/n' in size <7.5> not available
(Font)              size <7> substituted on input line 61.

LaTeX Font Info:    Trying to load font information for U+euf on input
line 61.

(c:/texlive/2023/texmf-dist/tex/latex/amsfonts/ueuf.fd
File: ueuf.fd 2013/01/14 v3.01 Euler Fraktur
) (c:/texlive/2023/texmf-dist/tex/latex/microtype/mt-euf.cfg
File: mt-euf.cfg 2006/07/03 v1.1 microtype config. file: AMS Euler
Fraktur (RS)

)
```

```
LaTeX Font Info:    Trying to load font information for U+eus on input
line 61.

(c:/texlive/2023/texmf-dist/tex/latex/amsfonts/ueus.fd
File: ueus.fd 2013/01/14 v3.01 Euler Script
) (c:/texlive/2023/texmf-dist/tex/latex/microtype/mt-eus.cfg
File: mt-eus.cfg 2006/07/28 v1.2 microtype config. file: AMS Euler Script
(RS)
)
LaTeX Font Info:    Trying to load font information for U+euex on input
line 61
.
(c:/texlive/2023/texmf-dist/tex/latex/amsfonts/ueuex.fd
File: ueuex.fd 2013/01/14 v3.01 Euler extra symbols
)

LaTeX Font Warning: Font shape `OML/cmm/m/it' in size <7.5> not available
(Font)              size <7> substituted on input line 61.

LaTeX Font Info:    Font shape `T1/Merriwthr-OsF/m/n' will be
(Font)              scaled to size 6.24973pt on input line 61.
LaTeX Font Info:    Font shape `T1/Merriwthr-OsF/m/n' will be
(Font)              scaled to size 5.24997pt on input line 61.
LaTeX Font Info:    Font shape `T1/Merriwthr-OsF/m/it' will be
(Font)              scaled to size 7.5pt on input line 61.
LaTeX Font Info:    Font shape `T1/Merriwthr-OsF/m/it' will be
(Font)              scaled to size 6.24973pt on input line 61.
LaTeX Font Info:    Font shape `T1/Merriwthr-OsF/m/it' will be
(Font)              scaled to size 5.24997pt on input line 61.
LaTeX Font Info:    Font shape `T1/Merriwthr-OsF/m/n' will be
(Font)              scaled to size 8.0pt on input line 61.
LaTeX Font Info:    Font shape `T1/Merriwthr-OsF/m/it' will be
(Font)              scaled to size 8.0pt on input line 61.
LaTeX Font Info:    Font shape `T1/Merriwthr-OsF/b/it' will be
(Font)              scaled to size 8.0pt on input line 61.
TextBlockOrigin set to 4pc+6.64pt x 4pc+6pt
<example-grid-100x100pt.png, id=20, 125.46875pt x 125.46875pt>
File: example-grid-100x100pt.png Graphic file (type png)
<use example-grid-100x100pt.png>
Package pdftex.def Info: example-grid-100x100pt.png  used on input line
68.
(pdftex.def)             Requested size: 42.00021pt x 42.0pt.

Overfull \hbox (54.64pt too wide) in paragraph at lines 68--68
[][]
 []

LaTeX Font Info:    Font shape `T1/Merriwthr-OsF/m/n' will be
(Font)              scaled to size 14.0pt on input line 68.
LaTeX Font Info:    Font shape `T1/Merriwthr-OsF/m/n' will be
(Font)              scaled to size 8.99997pt on input line 68.
LaTeX Font Info:    Calculating math sizes for size <14> on input line
68.
LaTeX Font Info:    Font shape `T1/Merriwthr-OsF/m/up' will be
```

```
(Font)              scaled to size 14.0pt on input line 68.
LaTeX Font Info:    Font shape `T1/Merriwthr-OsF/m/up' will be
(Font)              scaled to size 11.66617pt on input line 68.
LaTeX Font Info:    Font shape `T1/Merriwthr-OsF/m/up' will be
(Font)              scaled to size 9.79996pt on input line 68.
LaTeX Font Info:    Font shape `T1/Merriwthr-OsF/m/n' will be
(Font)              scaled to size 11.66617pt on input line 68.
LaTeX Font Info:    Font shape `T1/Merriwthr-OsF/m/n' will be
(Font)              scaled to size 9.79996pt on input line 68.
LaTeX Font Info:    Font shape `T1/Merriwthr-OsF/m/it' will be
(Font)              scaled to size 14.0pt on input line 68.
LaTeX Font Info:    Font shape `T1/Merriwthr-OsF/m/it' will be
(Font)              scaled to size 11.66617pt on input line 68.
LaTeX Font Info:    Font shape `T1/Merriwthr-OsF/m/it' will be
(Font)              scaled to size 9.79996pt on input line 68.
LaTeX Font Info:    Font shape `T1/Merriwthr-OsF/b/n' will be
(Font)              scaled to size 18.0pt on input line 68.
LaTeX Font Info:    Font shape `T1/Merriwthr-OsF/m/n' will be
(Font)              scaled to size 13.0pt on input line 68.
LaTeX Font Info:    Calculating math sizes for size <13> on input line
68.
LaTeX Font Info:    Font shape `T1/Merriwthr-OsF/m/up' will be
(Font)              scaled to size 13.0pt on input line 68.
LaTeX Font Info:    Font shape `T1/Merriwthr-OsF/m/up' will be
(Font)              scaled to size 10.83287pt on input line 68.
LaTeX Font Info:    Font shape `T1/Merriwthr-OsF/m/up' will be
(Font)              scaled to size 9.09996pt on input line 68.

LaTeX Font Warning: Font shape `OMS/cmsy/m/n' in size <13> not available
(Font)              size <12> substituted on input line 68.


LaTeX Font Warning: Font shape `OMX/cmex/m/n' in size <13> not available
(Font)              size <12> substituted on input line 68.


LaTeX Font Warning: Font shape `OML/cmm/m/it' in size <13> not available
(Font)              size <12> substituted on input line 68.

LaTeX Font Info:    Font shape `T1/Merriwthr-OsF/m/n' will be
(Font)              scaled to size 10.83287pt on input line 68.
LaTeX Font Info:    Font shape `T1/Merriwthr-OsF/m/n' will be
(Font)              scaled to size 9.09996pt on input line 68.
LaTeX Font Info:    Font shape `T1/Merriwthr-OsF/m/it' will be
(Font)              scaled to size 13.0pt on input line 68.
LaTeX Font Info:    Font shape `T1/Merriwthr-OsF/m/it' will be
(Font)              scaled to size 10.83287pt on input line 68.
LaTeX Font Info:    Font shape `T1/Merriwthr-OsF/m/it' will be
(Font)              scaled to size 9.09996pt on input line 68.
LaTeX Font Info:    Trying to load font information for TS1+Merriwthr-OsF
on in
put line 68.
(c:/texlive/2023/texmf-dist/tex/latex/merriweather/TS1Merriwthr-OsF.fd
```

```
File: TS1Merriwthr-OsF.fd 2020/08/30 (autoinst) Font definitions for
TS1/Merriw
thr-OsF.
)
LaTeX Font Info:    Font shape `TS1/Merriwthr-OsF/m/n' will be
(Font)              scaled to size 10.83287pt on input line 68.
Package microtype Info: Loading generic protrusion settings for font
family
(microtype)             `Merriwthr-OsF' (encoding: TS1).
(microtype)             For optimal results, create family-specific
settings.
(microtype)             See the microtype manual for details.
LaTeX Font Info:    Font shape `T1/Merriwthr-OsF/m/n' will be
(Font)              scaled to size 9.0pt on input line 68.
LaTeX Font Info:    Font shape `T1/Merriwthr-OsF/m/up' will be
(Font)              scaled to size 9.0pt on input line 68.
LaTeX Font Info:    Font shape `T1/Merriwthr-OsF/m/up' will be
(Font)              scaled to size 7.0pt on input line 68.
LaTeX Font Info:    Font shape `T1/Merriwthr-OsF/m/up' will be
(Font)              scaled to size 5.0pt on input line 68.
LaTeX Font Info:    Font shape `T1/Merriwthr-OsF/m/n' will be
(Font)              scaled to size 7.0pt on input line 68.
LaTeX Font Info:    Font shape `T1/Merriwthr-OsF/m/n' will be
(Font)              scaled to size 5.0pt on input line 68.
LaTeX Font Info:    Font shape `T1/Merriwthr-OsF/m/it' will be
(Font)              scaled to size 9.0pt on input line 68.
LaTeX Font Info:    Font shape `T1/Merriwthr-OsF/m/it' will be
(Font)              scaled to size 7.0pt on input line 68.
LaTeX Font Info:    Font shape `T1/Merriwthr-OsF/m/it' will be
(Font)              scaled to size 5.0pt on input line 68.
LaTeX Font Info:    Font shape `T1/Merriwthr-OsF/m/n' will be
(Font)              scaled to size 6.5pt on input line 68.
LaTeX Font Info:    Calculating math sizes for size <6.5> on input line
68.
LaTeX Font Info:    Font shape `T1/Merriwthr-OsF/m/up' will be
(Font)              scaled to size 6.5pt on input line 68.
LaTeX Font Info:    Font shape `T1/Merriwthr-OsF/m/up' will be
(Font)              scaled to size 5.41643pt on input line 68.
LaTeX Font Info:    Font shape `T1/Merriwthr-OsF/m/up' will be
(Font)              scaled to size 4.54997pt on input line 68.

LaTeX Font Warning: Font shape `OMS/cmsy/m/n' in size <6.5> not available
(Font)              size <6> substituted on input line 68.


LaTeX Font Warning: Font shape `OMS/cmsy/m/n' in size <5.41643> not
available
(Font)              size <5> substituted on input line 68.


LaTeX Font Warning: Font shape `OMS/cmsy/m/n' in size <4.54997> not
available
(Font)              size <5> substituted on input line 68.
```

```
LaTeX Font Warning: Font shape `OML/cmm/m/it' in size <6.5> not available
(Font)              size <6> substituted on input line 68.


LaTeX Font Warning: Font shape `OML/cmm/m/it' in size <5.41643> not
available
(Font)              size <5> substituted on input line 68.


LaTeX Font Warning: Font shape `OML/cmm/m/it' in size <4.54997> not
available
(Font)              size <5> substituted on input line 68.

LaTeX Font Info:    Font shape `T1/Merriwthr-OsF/m/n' will be
(Font)              scaled to size 5.41643pt on input line 68.
LaTeX Font Info:    Font shape `T1/Merriwthr-OsF/m/n' will be
(Font)              scaled to size 4.54997pt on input line 68.
LaTeX Font Info:    Font shape `T1/Merriwthr-OsF/m/it' will be
(Font)              scaled to size 6.5pt on input line 68.
LaTeX Font Info:    Font shape `T1/Merriwthr-OsF/m/it' will be
(Font)              scaled to size 5.41643pt on input line 68.
LaTeX Font Info:    Font shape `T1/Merriwthr-OsF/m/it' will be
(Font)              scaled to size 4.54997pt on input line 68.
LaTeX Font Info:    Font shape `TS1/Merriwthr-OsF/m/n' will be
(Font)              scaled to size 5.41643pt on input line 68.

Overfull \hbox (54.64pt too wide) in paragraph at lines 68--68
[][][]
 []

LaTeX Font Info:    Font shape `T1/Merriwthr-OsF/b/n' will be
(Font)              scaled to size 10.0pt on input line 68.

Overfull \hbox (54.64pt too wide) in paragraph at lines 68--68
[][][]
 []

LaTeX Font Info:    Font shape `T1/Merriwthr-OsF/b/n' will be
(Font)              scaled to size 7.0pt on input line 73.
LaTeX Font Info:    Font shape `T1/Merriwthr-OsF/m/up' will be
(Font)              scaled to size 7.5pt on input line 76.
LaTeX Font Info:    Font shape `T1/Merriwthr-OsF/b/n' will be
(Font)              scaled to size 7.5pt on input line 79.

Underfull \hbox (badness 10000) in paragraph at lines 81--81
[]|\T1/Merriwthr-OsF/m/up/7.5 (+20) What onco-genic ge-netic mu-ta-tions
are
 []


Underfull \hbox (badness 6859) in paragraph at lines 81--81
[]|\T1/Merriwthr-OsF/m/up/7.5 (+20) [`adenoid cys-tic car-ci-noma', orig-
i-nate
```

```
d_from, `FLT3-p.D835Y-
 []


Underfull \hbox (badness 10000) in paragraph at lines 81--81
\T1/Merriwthr-OsF/m/up/7.5 (+20) [`FLT3-p.D835Y-adenoid cys-tic car-ci-
noma', h
as_var, `FLT3-
 []


Overfull \hbox (13.74031pt too wide) in alignment at lines 76--82
[] [] [] []
 []

LaTeX Font Info:    Font shape `T1/Merriwthr-OsF/m/n' will be
(Font)              scaled to size 6.8438pt on input line 93.
LaTeX Font Info:    Calculating math sizes for size <6.8438> on input
line 93.
LaTeX Font Info:    Font shape `T1/Merriwthr-OsF/m/up' will be
(Font)              scaled to size 6.8438pt on input line 93.
LaTeX Font Info:    Font shape `T1/Merriwthr-OsF/m/up' will be
(Font)              scaled to size 5.70291pt on input line 93.
LaTeX Font Info:    Font shape `T1/Merriwthr-OsF/m/up' will be
(Font)              scaled to size 4.79063pt on input line 93.
LaTeX Font Info:    Font shape `T1/Merriwthr-OsF/m/n' will be
(Font)              scaled to size 5.70291pt on input line 93.
LaTeX Font Info:    Font shape `T1/Merriwthr-OsF/m/n' will be
(Font)              scaled to size 4.79063pt on input line 93.
LaTeX Font Info:    Font shape `T1/Merriwthr-OsF/m/it' will be
(Font)              scaled to size 6.8438pt on input line 93.
LaTeX Font Info:    Font shape `T1/Merriwthr-OsF/m/it' will be
(Font)              scaled to size 5.70291pt on input line 93.
LaTeX Font Info:    Font shape `T1/Merriwthr-OsF/m/it' will be
(Font)              scaled to size 4.79063pt on input line 93.

LaTeX Warning: `h' float specifier changed to `ht'.


LaTeX Warning: `h' float specifier changed to `ht'.

LaTeX Font Info:    Font shape `T1/Merriwthr-OsF/b/n' will be
(Font)              scaled to size 8.5pt on input line 124.

Package natbib Warning: Citation `stock2013personalised' on page 1
undefined on
 input line 126.


Package natbib Warning: Citation `wang2022ado' on page 1 undefined on
input lin
e 127.
```

```
Package natbib Warning: Citation `huang2020efficacy' on page 1 undefined
on inp
ut line 127.


Underfull \hbox (badness 1394) in paragraph at lines 148--149
[]|\T1/Merriwthr-OsF/m/n/7 (+20) The onco-genic mu-ta-tion gene that can
be tre
ated with ado-trastuzumab em-tan-sine is ERBB2-
 []


LaTeX Warning: `h' float specifier changed to `ht'.


Package natbib Warning: Citation `bodaghi2023biomarkers' on page 1
undefined on
 input line 154.


Package natbib Warning: Citation `davies2014pten' on page 1 undefined on
input
line 155.


Package natbib Warning: Citation `yang2013reduced' on page 1 undefined on
input
 line 155.


Underfull \vbox (badness 5231) has occurred while \output is active []

LaTeX Font Info:    Font shape `T1/Merriwthr-OsF/m/n' will be
(Font)              scaled to size 7.8pt on input line 157.
LaTeX Font Info:    Font shape `T1/Merriwthr-OsF/b/n' will be
(Font)              scaled to size 7.8pt on input line 157.
[1{c:/texlive/2023/texmf-
var/fonts/map/pdftex/updmap/pdftex.map}{c:/texlive/202
3/texmf-
dist/fonts/enc/dvips/merriweather/merriwthr_posqbl.enc}{c:/texlive/2023
/texmf-dist/fonts/enc/dvips/merriweather/merriwthr_owzwzj.enc}


 <c:/texlive/2023/texmf-dist/tex/latex/mwe/example-grid-100x100pt.png>]
Underfull \hbox (badness 6825) in paragraph at lines 169--170
[]|$\T1/Merriwthr-OsF/m/up/6.8438 (+20) ([]) \U/euex/m/n/6.8438
\T1/Merriwthr
-OsF/m/up/6.8438 (+20) [[]] ^^U ([]) ^^U [[]] \U/euex/m/n/6.8438 !
 []


LaTeX Warning: `h' float specifier changed to `ht'.

LaTeX Font Info:    Font shape `T1/Merriwthr-OsF/m/n' will be
```

```
(Font)              scaled to size 6.0pt on input line 207.
LaTeX Font Info:    Font shape `T1/Merriwthr-OsF/b/n' will be
(Font)              scaled to size 6.0pt on input line 207.

LaTeX Warning: `h' float specifier changed to `ht'.


LaTeX Warning: `h' float specifier changed to `ht'.


LaTeX Warning: `h' float specifier changed to `ht'.


LaTeX Warning: `h' float specifier changed to `ht'.



pdfTeX warning: pdflatex.exe (file ./examples.pdf): PDF inclusion: found
PDF ve
rsion <1.7>, but at most version <1.5> allowed
<examples.pdf, id=41, 438.67888pt x 501.79468pt>
File: examples.pdf Graphic file (type pdf)
<use examples.pdf>
Package pdftex.def Info: examples.pdf  used on input line 297.
(pdftex.def)             Requested size: 235.11394pt x 268.94371pt.

LaTeX Warning: `h' float specifier changed to `ht'.

LaTeX Font Info:    Font shape `T1/Merriwthr-OsF/m/it' will be
(Font)              scaled to size 7.8pt on input line 301.
[2] [3

 <./examples.pdf>] [4]
No file supplementary.bbl.

Package natbib Warning: There were undefined citations.


enddocument/afterlastpage: lastpage setting LastPage.
(./supplementary.aux)
 ***********
LaTeX2e <2023-11-01> patch level 1
L3 programming layer <2020/03/25>
 ***********

LaTeX Font Warning: Size substitutions with differences
(Font)              up to 1.0pt have occurred.


LaTeX Font Warning: Some font shapes were not available, defaults
substituted.


LaTeX Warning: There were multiply-defined labels.
```

```
Package rerunfilecheck Info: File `supplementary.out' has not changed.
(rerunfilecheck)             Checksum: 
5DBAE73A310F25B7C5535B3347B5085D;789.
 )
Here is how much of TeX's memory you used:
 35156 strings out of 474121
 706428 string characters out of 5747949
 1980190 words of memory out of 5000000
 56439 multiletter control sequences out of 15000+600000
 1907820 words of font info for 467 fonts, out of 8000000 for 9000
 1141 hyphenation exceptions out of 8191
 123i,15n,131p,1010b,950s stack positions out of 
10000i,1000n,20000p,200000b,200000s
<c:/texlive/2023/texmf-dist/fonts/type1/sorkin/merriweather/Merriwthr-
Bold.pf
b><c:/texlive/2023/texmf-dist/fonts/type1/sorkin/merriweather/Merriwthr-
BoldIta
lic.pfb><c:/texlive/2023/texmf-
dist/fonts/type1/sorkin/merriweather/Merriwthr-I
talic.pfb><c:/texlive/2023/texmf-
dist/fonts/type1/sorkin/merriweather/Merriwthr
-Regular.pfb><c:/texlive/2023/texmf-
dist/fonts/type1/public/amsfonts/euler/euex
7.pfb>
Output written on supplementary.pdf (4 pages, 287713 bytes).
PDF statistics:
 105 PDF objects out of 1000 (max. 8388607)
 82 compressed objects within 1 object stream
 20 named destinations out of 1000 (max. 500000)
 196655 words of extra memory for PDF output out of 221844 (max. 
10000000)
```

Click here to access/download;LaTeX - Main Document (TeX file);manuscript_file.tex

```
This is pdfTeX, Version 3.141592653-2.6-1.40.25 (TeX Live 2023) (preloaded format=pdflatex 2024.3.8)  1 OCT 2024 04:11
entering extended mode
 restricted \write18 enabled.
 %&-line parsing enabled.
**manuscript_file.tex
(./manuscript_file.tex
LaTeX2e <2023-11-01> patch level 1
L3 programming layer <2024-02-20>
(./oup-contemporary.cls
Document Class: oup-contemporary 2023/06/12, v1.2
(c:/texlive/2023/texmf-dist/tex/latex/base/article.cls
Document Class: article 2023/05/17 v1.4n Standard LaTeX document class
(c:/texlive/2023/texmf-dist/tex/latex/base/size10.clo
File: size10.clo 2023/05/17 v1.4n Standard LaTeX file (size option)
)
\c@part=\count188
\c@section=\count189
\c@subsection=\count190
\c@subsubsection=\count191
\c@paragraph=\count192
\c@subparagraph=\count193
\c@figure=\count194
\c@table=\count195
\abovecaptionskip=\skip48
\belowcaptionskip=\skip49
\bibindent=\dimen140
) (c:/texlive/2023/texmf-dist/tex/latex/base/inputenc.sty
Package: inputenc 2021/02/14 v1.3d Input encoding file
\inpenc@prehook=\toks17
\inpenc@posthook=\toks18
) (c:/texlive/2023/texmf-dist/tex/latex/base/fontenc.sty
Package: fontenc 2021/04/29 v2.0v Standard LaTeX package
) (c:/texlive/2023/texmf-dist/tex/generic/iftex/ifpdf.sty
Package: ifpdf 2019/10/25 v3.4 ifpdf legacy package. Use iftex instead.
(c:/texlive/2023/texmf-dist/tex/generic/iftex/iftex.sty
Package: iftex 2022/02/03 v1.0f TeX engine tests
)) (c:/texlive/2023/texmf-dist/tex/latex/microtype/microtype.sty
Package: microtype 2023/03/13 v3.1a Micro-typographical refinements (RS)
(c:/texlive/2023/texmf-dist/tex/latex/graphics/keyval.sty
Package: keyval 2022/05/29 v1.15 key=value parser (DPC)
\KV@toks@=\toks19
) (c:/texlive/2023/texmf-dist/tex/latex/etoolbox/etoolbox.sty
Package: etoolbox 2020/10/05 v2.5k e-TeX tools for LaTeX (JAW)
\etb@tempcnta=\count196
)
\MT@toks=\toks20
\MT@tempbox=\box51
\MT@count=\count197
LaTeX Info: Redefining \noprotrusionifhmode on input line 1059.
LaTeX Info: Redefining \leftprotrusion on input line 1060.
\MT@prot@toks=\toks21
LaTeX Info: Redefining \rightprotrusion on input line 1078.
LaTeX Info: Redefining \textls on input line 1368.
```

```
\MT@outer@kern=\dimen141
LaTeX Info: Redefining \textmicrotypecontext on input line 1988.
\MT@listname@count=\count198
(c:/texlive/2023/texmf-dist/tex/latex/microtype/microtype-pdftex.def
File: microtype-pdftex.def 2023/03/13 v3.1a Definitions specific to
pdftex (RS)

LaTeX Info: Redefining \lsstyle on input line 902.
LaTeX Info: Redefining \lslig on input line 902.
\MT@outer@space=\skip50
)
Package microtype Info: Loading configuration file microtype.cfg.
(c:/texlive/2023/texmf-dist/tex/latex/microtype/microtype.cfg
File: microtype.cfg 2023/03/13 v3.1a microtype main configuration file
(RS)
)) (c:/texlive/2023/texmf-dist/tex/latex/euler/euler.sty
Package: euler 1995/03/05 v2.5
Package: `euler' v2.5 <1995/03/05> (FJ and FMi)
LaTeX Font Info:    Redeclaring symbol font `letters' on input line 35.
LaTeX Font Info:    Encoding `OML' has changed to `U' for symbol font
(Font)              `letters' in the math version `normal' on input line
35.
LaTeX Font Info:    Overwriting symbol font `letters' in version `normal'
(Font)                  OML/cmm/m/it --> U/eur/m/n on input line 35.
LaTeX Font Info:    Encoding `OML' has changed to `U' for symbol font
(Font)              `letters' in the math version `bold' on input line
35.
LaTeX Font Info:    Overwriting symbol font `letters' in version `bold'
(Font)                  OML/cmm/b/it --> U/eur/m/n on input line 35.
LaTeX Font Info:    Overwriting symbol font `letters' in version `bold'
(Font)                  U/eur/m/n --> U/eur/b/n on input line 36.
LaTeX Font Info:    Redeclaring math symbol \Gamma on input line 47.
LaTeX Font Info:    Redeclaring math symbol \Delta on input line 48.
LaTeX Font Info:    Redeclaring math symbol \Theta on input line 49.
LaTeX Font Info:    Redeclaring math symbol \Lambda on input line 50.
LaTeX Font Info:    Redeclaring math symbol \Xi on input line 51.
LaTeX Font Info:    Redeclaring math symbol \Pi on input line 52.
LaTeX Font Info:    Redeclaring math symbol \Sigma on input line 53.
LaTeX Font Info:    Redeclaring math symbol \Upsilon on input line 54.
LaTeX Font Info:    Redeclaring math symbol \Phi on input line 55.
LaTeX Font Info:    Redeclaring math symbol \Psi on input line 56.
LaTeX Font Info:    Redeclaring math symbol \Omega on input line 57.
\symEulerFraktur=\mathgroup4
LaTeX Font Info:    Overwriting symbol font `EulerFraktur' in version
`bold'
(Font)                  U/euf/m/n --> U/euf/b/n on input line 63.
LaTeX Info: Redefining \oldstylenums on input line 85.
\symEulerScript=\mathgroup5
LaTeX Font Info:    Overwriting symbol font `EulerScript' in version
`bold'
(Font)                  U/eus/m/n --> U/eus/b/n on input line 93.
LaTeX Font Info:    Redeclaring math symbol \aleph on input line 97.
LaTeX Font Info:    Redeclaring math symbol \Re on input line 98.
LaTeX Font Info:    Redeclaring math symbol \Im on input line 99.
```

```
LaTeX Font Info:    Redeclaring math delimiter \vert on input line 101.
LaTeX Font Info:    Redeclaring math delimiter \backslash on input line
103.
LaTeX Font Info:    Redeclaring math symbol \neg on input line 106.
LaTeX Font Info:    Redeclaring math symbol \wedge on input line 108.
LaTeX Font Info:    Redeclaring math symbol \vee on input line 110.
LaTeX Font Info:    Redeclaring math symbol \setminus on input line 112.
LaTeX Font Info:    Redeclaring math symbol \sim on input line 113.
LaTeX Font Info:    Redeclaring math symbol \mid on input line 114.
LaTeX Font Info:    Redeclaring math delimiter \arrowvert on input line
116.
LaTeX Font Info:    Redeclaring math symbol \mathsection on input line
117.
\symEulerExtension=\mathgroup6
LaTeX Font Info:    Redeclaring math symbol \coprod on input line 125.
LaTeX Font Info:    Redeclaring math symbol \prod on input line 125.
LaTeX Font Info:    Redeclaring math symbol \sum on input line 125.
LaTeX Font Info:    Redeclaring math symbol \intop on input line 130.
LaTeX Font Info:    Redeclaring math symbol \ointop on input line 131.
LaTeX Font Info:    Redeclaring math symbol \braceld on input line 132.
LaTeX Font Info:    Redeclaring math symbol \bracerd on input line 133.
LaTeX Font Info:    Redeclaring math symbol \bracelu on input line 134.
LaTeX Font Info:    Redeclaring math symbol \braceru on input line 135.
LaTeX Font Info:    Redeclaring math symbol \infty on input line 136.
LaTeX Font Info:    Redeclaring math symbol \nearrow on input line 153.
LaTeX Font Info:    Redeclaring math symbol \searrow on input line 154.
LaTeX Font Info:    Redeclaring math symbol \nwarrow on input line 155.
LaTeX Font Info:    Redeclaring math symbol \swarrow on input line 156.
LaTeX Font Info:    Redeclaring math symbol \Leftrightarrow on input line
157.
LaTeX Font Info:    Redeclaring math symbol \Leftarrow on input line 158.
LaTeX Font Info:    Redeclaring math symbol \Rightarrow on input line
159.
LaTeX Font Info:    Redeclaring math symbol \leftrightarrow on input line
160.
LaTeX Font Info:    Redeclaring math symbol \leftarrow on input line 161.
LaTeX Font Info:    Redeclaring math symbol \rightarrow on input line
163.
LaTeX Font Info:    Redeclaring math delimiter \uparrow on input line
166.
LaTeX Font Info:    Redeclaring math delimiter \downarrow on input line
168.
LaTeX Font Info:    Redeclaring math delimiter \updownarrow on input line
170.
LaTeX Font Info:    Redeclaring math delimiter \Uparrow on input line
172.
LaTeX Font Info:    Redeclaring math delimiter \Downarrow on input line
174.
LaTeX Font Info:    Redeclaring math delimiter \Updownarrow on input line
176.
LaTeX Font Info:    Redeclaring math symbol \leftharpoonup on input line
177.
LaTeX Font Info:    Redeclaring math symbol \leftharpoondown on input
line 178.
```

```
LaTeX Font Info:    Redeclaring math symbol \rightharpoonup on input line
179.
LaTeX Font Info:    Redeclaring math symbol \rightharpoondown on input
line 180
.
LaTeX Font Info:    Redeclaring math delimiter \lbrace on input line 182.
LaTeX Font Info:    Redeclaring math delimiter \rbrace on input line 184.
\symcmmigroup=\mathgroup7
LaTeX Font Info:    Overwriting symbol font `cmmigroup' in version `bold'
(Font)                  OML/cmm/m/it --> OML/cmm/b/it on input line 200.
LaTeX Font Info:    Redeclaring math accent \vec on input line 201.
LaTeX Font Info:    Redeclaring math symbol \triangleleft on input line
202.
LaTeX Font Info:    Redeclaring math symbol \triangleright on input line
203.
LaTeX Font Info:    Redeclaring math symbol \star on input line 204.
LaTeX Font Info:    Redeclaring math symbol \lhook on input line 205.
LaTeX Font Info:    Redeclaring math symbol \rhook on input line 206.
LaTeX Font Info:    Redeclaring math symbol \flat on input line 207.
LaTeX Font Info:    Redeclaring math symbol \natural on input line 208.
LaTeX Font Info:    Redeclaring math symbol \sharp on input line 209.
LaTeX Font Info:    Redeclaring math symbol \smile on input line 210.
LaTeX Font Info:    Redeclaring math symbol \frown on input line 211.
LaTeX Font Info:    Redeclaring math accent \grave on input line 245.
LaTeX Font Info:    Redeclaring math accent \acute on input line 246.
LaTeX Font Info:    Redeclaring math accent \tilde on input line 247.
LaTeX Font Info:    Redeclaring math accent \ddot on input line 248.
LaTeX Font Info:    Redeclaring math accent \check on input line 249.
LaTeX Font Info:    Redeclaring math accent \breve on input line 250.
LaTeX Font Info:    Redeclaring math accent \bar on input line 251.
LaTeX Font Info:    Redeclaring math accent \dot on input line 252.
LaTeX Font Info:    Redeclaring math accent \hat on input line 254.
) (c:/texlive/2023/texmf-dist/tex/latex/multirow/multirow.sty
Package: multirow 2021/03/15 v2.8 Span multiple rows of a table
\multirow@colwidth=\skip51
\multirow@cntb=\count199
\multirow@dima=\skip52
\bigstrutjot=\dimen142
) (c:/texlive/2023/texmf-dist/tex/latex/algorithms/algorithm.sty
Package: algorithm 2009/08/24 v0.1 Document Style `algorithm' - floating
enviro
nment
(c:/texlive/2023/texmf-dist/tex/latex/float/float.sty
Package: float 2001/11/08 v1.3d Float enhancements (AL)
\c@float@type=\count266
\float@exts=\toks22
\float@box=\box52
\@float@everytoks=\toks23
\@floatcapt=\box53
) (c:/texlive/2023/texmf-dist/tex/latex/base/ifthen.sty
Package: ifthen 2022/04/13 v1.1d Standard LaTeX ifthen package (DPC)
)
\@float@every@algorithm=\toks24
```

```
\c@algorithm=\count267
) (c:/texlive/2023/texmf-dist/tex/latex/algorithmicx/algorithmicx.sty
Package: algorithmicx 2005/04/27 v1.2 Algorithmicx
Document Style algorithmicx 1.2 - a greatly improved `algorithmic' style
\c@ALG@line=\count268
\c@ALG@rem=\count269
\c@ALG@nested=\count270
\ALG@tlm=\skip53
\ALG@thistlm=\skip54
\c@ALG@Lnr=\count271
\c@ALG@blocknr=\count272
\c@ALG@storecount=\count273
\c@ALG@tmpcounter=\count274
\ALG@tmplength=\skip55
) (c:/texlive/2023/texmf-dist/tex/latex/merriweather/merriweather.sty
Package: merriweather 2022/09/20 (Bob Tennent) Supports
Merriweather(Sans) font
s for all LaTeX engines.
(c:/texlive/2023/texmf-dist/tex/generic/iftex/ifxetex.sty
Package: ifxetex 2019/10/25 v0.7 ifxetex legacy package. Use iftex
instead.
) (c:/texlive/2023/texmf-dist/tex/generic/iftex/ifluatex.sty
Package: ifluatex 2019/10/25 v1.5 ifluatex legacy package. Use iftex
instead.
) (c:/texlive/2023/texmf-dist/tex/latex/base/textcomp.sty
Package: textcomp 2020/02/02 v2.0n Standard LaTeX package
) (c:/texlive/2023/texmf-dist/tex/latex/xkeyval/xkeyval.sty
Package: xkeyval 2022/06/16 v2.9 package option processing (HA)
(c:/texlive/2023/texmf-dist/tex/generic/xkeyval/xkeyval.tex
(c:/texlive/2023/te
xmf-dist/tex/generic/xkeyval/xkvutils.tex
\XKV@toks=\toks25
\XKV@tempa@toks=\toks26
)
\XKV@depth=\count275
File: xkeyval.tex 2014/12/03 v2.7a key=value parser (HA)
)) (c:/texlive/2023/texmf-dist/tex/latex/base/fontenc.sty
Package: fontenc 2021/04/29 v2.0v Standard LaTeX package
) (c:/texlive/2023/texmf-dist/tex/latex/fontaxes/fontaxes.sty
Package: fontaxes 2020/07/21 v1.0e Font selection axes
LaTeX Info: Redefining \upshape on input line 29.
LaTeX Info: Redefining \itshape on input line 31.
LaTeX Info: Redefining \slshape on input line 33.
LaTeX Info: Redefining \swshape on input line 35.
LaTeX Info: Redefining \scshape on input line 37.
LaTeX Info: Redefining \sscshape on input line 39.
LaTeX Info: Redefining \ulcshape on input line 41.
LaTeX Info: Redefining \textsw on input line 47.
LaTeX Info: Redefining \textssc on input line 48.
LaTeX Info: Redefining \textulc on input line 49.
)) (c:/texlive/2023/texmf-dist/tex/latex/mathastext/mathastext.sty
Package: mathastext 2023/12/29 v1.3zb Use the text font in math mode
(JFB)
```

```
Package mathastext Info: Starting the math mode configuration.
\mst@exists@muskip=\muskip16
\mst@forall@muskip=\muskip17
\mst@prime@muskip=\muskip18
\mst@do@nonletters=\toks27
\mst@do@easynonletters=\toks28
\mst@do@az=\toks29
\mst@do@AZ=\toks30
\symmtoperatorfont=\mathgroup8
\symmtletterfont=\mathgroup9
(   mathastext:   ) ! and ?
(   mathastext:   ) punctuation: , . : ; and \colon
LaTeX Info: Redefining \relbar on input line 894.
LaTeX Info: Redefining \rightarrowfill on input line 897.
LaTeX Info: Redefining \leftarrowfill on input line 902.
(   mathastext:   ) + and =
LaTeX Info: Redefining \Relbar on input line 993.
(   mathastext:   ) adding = ; and + to \nfss@catcodes
(   mathastext:   ) parentheses ( ) [ ] and slash /
(   mathastext:   ) alldelims: < > \backslash \setminus | \vert \mid \{
\}
LaTeX Font Info:    Redeclaring math delimiter \backslash on input line
1039.
LaTeX Font Info:    Redeclaring math symbol \setminus on input line 1051.
LaTeX Info: Redefining \models on input line 1060.
(   mathastext:   ) \# \mathdollar \% \&
(   mathastext:   ) \imath and \jmath
LaTeX Font Info:    Overwriting math alphabet `\Mathnormalbold' in
version `nor
mal'
(Font)                  T1/Merriwthr-OsF/b/it --> T1/Merriwthr-OsF/b/it
on inpu
t line 2516.
LaTeX Font Info:    Overwriting math alphabet `\Mathnormalbold' in
version `bol
d'
(Font)                  T1/Merriwthr-OsF/b/it --> T1/Merriwthr-OsF/b/it
on inpu
t line 2516.
LaTeX Font Info:    Overwriting symbol font `mtletterfont' in version
`normal'
(Font)                  T1/Merriwthr-OsF/m/it --> T1/Merriwthr-OsF/m/it
on inpu
t line 2516.
LaTeX Font Info:    Overwriting symbol font `mtletterfont' in version
`bold'
(Font)                  T1/Merriwthr-OsF/m/it --> T1/Merriwthr-OsF/b/it
on inpu
t line 2516.
LaTeX Font Info:    Overwriting symbol font `mtoperatorfont' in version
`normal
'
(Font)                  T1/Merriwthr-OsF/m/n --> T1/Merriwthr-OsF/m/n on
input
```

```
line 2516.
LaTeX Font Info:    Overwriting symbol font `mtoperatorfont' in version
`bold'
(Font)                  T1/Merriwthr-OsF/m/n --> T1/Merriwthr-OsF/b/n on
input
line 2516.
LaTeX Font Info:    Overwriting math alphabet `\Mathbf' in version
`normal'
(Font)                  T1/Merriwthr-OsF/b/n --> T1/Merriwthr-OsF/b/n on
input
line 2516.
LaTeX Font Info:    Overwriting math alphabet `\Mathbf' in version `bold'
(Font)                  T1/Merriwthr-OsF/b/n --> T1/Merriwthr-OsF/b/n on
input
line 2516.
LaTeX Font Info:    Overwriting math alphabet `\Mathit' in version
`normal'
(Font)                  T1/Merriwthr-OsF/m/it --> T1/Merriwthr-OsF/m/it
on inpu
t line 2516.
LaTeX Font Info:    Overwriting math alphabet `\Mathit' in version `bold'
(Font)                  T1/Merriwthr-OsF/m/it --> T1/Merriwthr-OsF/b/it
on inpu
t line 2516.
LaTeX Font Info:    Overwriting math alphabet `\Mathsf' in version
`normal'
(Font)                  T1/MerriwthrSans-OsF/m/n --> T1/MerriwthrSans-
OsF/m/n o
n input line 2516.
LaTeX Font Info:    Overwriting math alphabet `\Mathsf' in version `bold'
(Font)                  T1/MerriwthrSans-OsF/m/n --> T1/MerriwthrSans-
OsF/b/n o
n input line 2516.
LaTeX Font Info:    Overwriting math alphabet `\Mathtt' in version
`normal'
(Font)                  T1/lmtt/m/n --> T1/lmtt/m/n on input line 2516.
LaTeX Font Info:    Overwriting math alphabet `\Mathtt' in version `bold'
(Font)                  T1/lmtt/m/n --> T1/lmtt/b/n on input line 2516.
(   mathastext:   ) Latin letters in the `normal', resp. `bold',
(   mathastext:   ) math versions are now set up to use the fonts
(   mathastext:   ) T1/Merriwthr-OsF/m/it, resp. T1/Merriwthr-OsF/b/it.
(   mathastext:   ) Other characters (digits, ...) and \log-like names
will be
(   mathastext:   ) typeset with the n shape.
(   mathastext:   ) \hbar
(   mathastext:   ) minus as endash
(   mathastext:   ) The italic option is in effect.
(   mathastext:   ) \HUGE has been (re)-defined.
(   mathastext:   ) mathastext has declared larger sizes for subscripts.
(   mathastext:   ) To keep LaTeX defaults, use option
`defaultmathsizes'.

Package mathastext Info: Loading is complete.  You can now use
\Mathastext to
```

```
(mathastext)              modify the normal and bold math versions.  Use
it
(mathastext)              with optional argument or use \MTDeclareVersion
to
(mathastext)              declare additional math versions.
) (c:/texlive/2023/texmf-dist/tex/latex/relsize/relsize.sty
Package: relsize 2013/03/29 ver 4.1
) (c:/texlive/2023/texmf-dist/tex/latex/ragged2e/ragged2e.sty
Package: ragged2e 2023/06/22 v3.6 ragged2e Package
\CenteringLeftskip=\skip56
\RaggedLeftLeftskip=\skip57
\RaggedRightLeftskip=\skip58
\CenteringRightskip=\skip59
\RaggedLeftRightskip=\skip60
\RaggedRightRightskip=\skip61
\CenteringParfillskip=\skip62
\RaggedLeftParfillskip=\skip63
\RaggedRightParfillskip=\skip64
\JustifyingParfillskip=\skip65
\CenteringParindent=\skip66
\RaggedLeftParindent=\skip67
\RaggedRightParindent=\skip68
\JustifyingParindent=\skip69
) (c:/texlive/2023/texmf-dist/tex/latex/xcolor/xcolor.sty
Package: xcolor 2023/11/15 v3.01 LaTeX color extensions (UK)
(c:/texlive/2023/texmf-dist/tex/latex/graphics-cfg/color.cfg
File: color.cfg 2016/01/02 v1.6 sample color configuration
)
Package xcolor Info: Driver file: pdftex.def on input line 274.
(c:/texlive/2023/texmf-dist/tex/latex/graphics-def/pdftex.def
File: pdftex.def 2022/09/22 v1.2b Graphics/color driver for pdftex
) (c:/texlive/2023/texmf-dist/tex/latex/graphics/mathcolor.ltx)
Package xcolor Info: Model `cmy' substituted by `cmy0' on input line
1350.
Package xcolor Info: Model `hsb' substituted by `rgb' on input line 1354.
Package xcolor Info: Model `RGB' extended on input line 1366.
Package xcolor Info: Model `HTML' substituted by `rgb' on input line
1368.
Package xcolor Info: Model `Hsb' substituted by `hsb' on input line 1369.
Package xcolor Info: Model `tHsb' substituted by `hsb' on input line
1370.
Package xcolor Info: Model `HSB' substituted by `hsb' on input line 1371.
Package xcolor Info: Model `Gray' substituted by `gray' on input line
1372.
Package xcolor Info: Model `wave' substituted by `hsb' on input line
1373.
) (c:/texlive/2023/texmf-dist/tex/latex/colortbl/colortbl.sty
Package: colortbl 2024/02/20 v1.0g Color table columns (DPC)
(c:/texlive/2023/texmf-dist/tex/latex/tools/array.sty
Package: array 2023/10/16 v2.5g Tabular extension package (FMi)
\col@sep=\dimen143
\ar@mcellbox=\box54
\extrarowheight=\dimen144
\NC@list=\toks31
```

```
\extratabsurround=\skip70
\backup@length=\skip71
\ar@cellbox=\box55
)
\everycr=\toks32
\minrowclearance=\skip72
\rownum=\count276
) (c:/texlive/2023/texmf-dist/tex/latex/graphics/graphicx.sty
Package: graphicx 2021/09/16 v1.2d Enhanced LaTeX Graphics (DPC,SPQR)
(c:/texlive/2023/texmf-dist/tex/latex/graphics/graphics.sty
Package: graphics 2022/03/10 v1.4e Standard LaTeX Graphics (DPC,SPQR)
(c:/texlive/2023/texmf-dist/tex/latex/graphics/trig.sty
Package: trig 2021/08/11 v1.11 sin cos tan (DPC)
) (c:/texlive/2023/texmf-dist/tex/latex/graphics-cfg/graphics.cfg
File: graphics.cfg 2016/06/04 v1.11 sample graphics configuration
)
Package graphics Info: Driver file: pdftex.def on input line 107.
)
\Gin@req@height=\dimen145
\Gin@req@width=\dimen146
) (c:/texlive/2023/texmf-dist/tex/latex/xpatch/xpatch.sty
(c:/texlive/2023/texm
f-dist/tex/latex/l3kernel/expl3.sty
Package: expl3 2024-02-20 L3 programming layer (loader)
(c:/texlive/2023/texmf-dist/tex/latex/l3backend/l3backend-pdftex.def
File: l3backend-pdftex.def 2024-02-20 L3 backend support: PDF output
(pdfTeX)
\l__color_backend_stack_int=\count277
\l__pdf_internal_box=\box56
))
Package: xpatch 2020/03/25 v0.3a Extending etoolbox patching commands
(c:/texlive/2023/texmf-dist/tex/latex/l3packages/xparse/xparse.sty
Package: xparse 2024-02-18 L3 Experimental document command parser
)) (c:/texlive/2023/texmf-dist/tex/latex/environ/environ.sty
Package: environ 2014/05/04 v0.3 A new way to define environments
(c:/texlive/2023/texmf-dist/tex/latex/trimspaces/trimspaces.sty
Package: trimspaces 2009/09/17 v1.1 Trim spaces around a token list
)
\@envbody=\toks33
) (c:/texlive/2023/texmf-dist/tex/latex/lastpage/lastpage.sty
Package: lastpage 2023/10/14 v2.0e lastpage: 2.09 or 2e? (HMM)
(c:/texlive/2023/texmf-dist/tex/latex/lastpage/lastpage2e.sty
Package: lastpage2e 2023/10/14 v2.0e Decide which 2e lastpage version to
use (H
MM)
(c:/texlive/2023/texmf-dist/tex/latex/lastpage/lastpagemodern.sty
Package: lastpagemodern 2023-10-14 v2.0e Refers to last page's name (HMM;
JPG)
\c@lastpagecount=\count278
)
)) (c:/texlive/2023/texmf-dist/tex/latex/graphics/rotating.sty
Package: rotating 2016/08/11 v2.16d rotated objects in LaTeX
\c@r@tfl@t=\count279
\rotFPtop=\skip73
```

```
\rotFPbot=\skip74
\rot@float@box=\box57
\rot@mess@toks=\toks34
) (c:/texlive/2023/texmf-dist/tex/latex/graphics/lscape.sty
Package: lscape 2020/05/28 v3.02 Landscape Pages (DPC)
) (c:/texlive/2023/texmf-dist/tex/latex/tools/afterpage.sty
Package: afterpage 2023/07/04 v1.08 After-Page Package (DPC)
\AP@output=\toks35
\AP@partial=\box58
\AP@footins=\box59
) (c:/texlive/2023/texmf-dist/tex/latex/textpos/textpos.sty
Package: textpos 2022/07/23 v1.10.1
Package textpos Info: choosing support for LaTeX3 on input line 60.
\TP@textbox=\box60
\TP@holdbox=\box61
\TPHorizModule=\dimen147
\TPVertModule=\dimen148
\TP@margin=\dimen149
\TP@absmargin=\dimen150
Grid set 16 x 16 = 37.34424pt x 52.81541pt
\TPboxrulesize=\dimen151
\TP@ox=\dimen152
\TP@oy=\dimen153
\TP@tbargs=\toks36
TextBlockOrigin set to 0pt x 0pt
) (c:/texlive/2023/texmf-dist/tex/latex/url/url.sty
\Urlmuskip=\muskip19
Package: url 2013/09/16  ver 3.4  Verb mode for urls, etc.
) (c:/texlive/2023/texmf-dist/tex/latex/newfloat/newfloat.sty
Package: newfloat 2023/10/01 v1.2 Defining new floating environments (AR)
Package newfloat Info: `rotating' package detected.
) (c:/texlive/2023/texmf-dist/tex/latex/mdframed/mdframed.sty
Package: mdframed 2013/07/01 1.9b: mdframed
(c:/texlive/2023/texmf-dist/tex/latex/kvoptions/kvoptions.sty
Package: kvoptions 2022-06-15 v3.15 Key value format for package options
(HO)
(c:/texlive/2023/texmf-dist/tex/generic/ltxcmds/ltxcmds.sty
Package: ltxcmds 2023-12-04 v1.26 LaTeX kernel commands for general use
(HO)
) (c:/texlive/2023/texmf-dist/tex/latex/kvsetkeys/kvsetkeys.sty
Package: kvsetkeys 2022-10-05 v1.19 Key value parser (HO)
)) (c:/texlive/2023/texmf-dist/tex/latex/zref/zref-abspage.sty
Package: zref-abspage 2023-09-14 v2.35 Module abspage for zref (HO)
(c:/texlive/2023/texmf-dist/tex/latex/zref/zref-base.sty
Package: zref-base 2023-09-14 v2.35 Module base for zref (HO)
(c:/texlive/2023/texmf-dist/tex/generic/infwarerr/infwarerr.sty
Package: infwarerr 2019/12/03 v1.5 Providing info/warning/error messages
(HO)
) (c:/texlive/2023/texmf-dist/tex/generic/kvdefinekeys/kvdefinekeys.sty
Package: kvdefinekeys 2019-12-19 v1.6 Define keys (HO)
) (c:/texlive/2023/texmf-dist/tex/generic/pdftexcmds/pdftexcmds.sty
Package: pdftexcmds 2020-06-27 v0.33 Utility functions of pdfTeX for
LuaTeX (HO
)
```

```
Package pdftexcmds Info: \pdf@primitive is available.
Package pdftexcmds Info: \pdf@ifprimitive is available.
Package pdftexcmds Info: \pdfdraftmode found.
) (c:/texlive/2023/texmf-dist/tex/generic/etexcmds/etexcmds.sty
Package: etexcmds 2019/12/15 v1.7 Avoid name clashes with e-TeX commands
(HO)
) (c:/texlive/2023/texmf-dist/tex/latex/auxhook/auxhook.sty
Package: auxhook 2019-12-17 v1.6 Hooks for auxiliary files (HO)
)
Package zref Info: New property list: main on input line 767.
Package zref Info: New property: default on input line 768.
Package zref Info: New property: page on input line 769.
)
\c@abspage=\count280
Package zref Info: New property: abspage on input line 67.
) (c:/texlive/2023/texmf-dist/tex/latex/needspace/needspace.sty
Package: needspace 2010/09/12 v1.3d reserve vertical space
)
\mdf@templength=\skip75
\c@mdf@globalstyle@cnt=\count281
\mdf@skipabove@length=\skip76
\mdf@skipbelow@length=\skip77
\mdf@leftmargin@length=\skip78
\mdf@rightmargin@length=\skip79
\mdf@innerleftmargin@length=\skip80
\mdf@innerrightmargin@length=\skip81
\mdf@innertopmargin@length=\skip82
\mdf@innerbottommargin@length=\skip83
\mdf@splittopskip@length=\skip84
\mdf@splitbottomskip@length=\skip85
\mdf@outermargin@length=\skip86
\mdf@innermargin@length=\skip87
\mdf@linewidth@length=\skip88
\mdf@innerlinewidth@length=\skip89
\mdf@middlelinewidth@length=\skip90
\mdf@outerlinewidth@length=\skip91
\mdf@roundcorner@length=\skip92
\mdf@footenotedistance@length=\skip93
\mdf@userdefinedwidth@length=\skip94
\mdf@needspace@length=\skip95
\mdf@frametitleaboveskip@length=\skip96
\mdf@frametitlebelowskip@length=\skip97
\mdf@frametitlerulewidth@length=\skip98
\mdf@frametitleleftmargin@length=\skip99
\mdf@frametitlerightmargin@length=\skip100
\mdf@shadowsize@length=\skip101
\mdf@extratopheight@length=\skip102
\mdf@subtitleabovelinewidth@length=\skip103
\mdf@subtitlebelowlinewidth@length=\skip104
\mdf@subtitleaboveskip@length=\skip105
\mdf@subtitlebelowskip@length=\skip106
\mdf@subtitleinneraboveskip@length=\skip107
\mdf@subtitleinnerbelowskip@length=\skip108
\mdf@subsubtitleabovelinewidth@length=\skip109
```

```
\mdf@subsubtitlebelowlinewidth@length=\skip110
\mdf@subsubtitleaboveskip@length=\skip111
\mdf@subsubtitlebelowskip@length=\skip112
\mdf@subsubtitleinneraboveskip@length=\skip113
\mdf@subsubtitleinnerbelowskip@length=\skip114
(c:/texlive/2023/texmf-dist/tex/latex/mdframed/md-frame-0.mdf
File: md-frame-0.mdf 2013/07/01\ 1.9b: md-frame-0
)
\mdf@frametitlebox=\box62
\mdf@footnotebox=\box63
\mdf@splitbox@one=\box64
\mdf@splitbox@two=\box65
\mdf@splitbox@save=\box66
\mdfsplitboxwidth=\skip115
\mdfsplitboxtotalwidth=\skip116
\mdfsplitboxheight=\skip117
\mdfsplitboxdepth=\skip118
\mdfsplitboxtotalheight=\skip119
\mdfframetitleboxwidth=\skip120
\mdfframetitleboxtotalwidth=\skip121
\mdfframetitleboxheight=\skip122
\mdfframetitleboxdepth=\skip123
\mdfframetitleboxtotalheight=\skip124
\mdffootnoteboxwidth=\skip125
\mdffootnoteboxtotalwidth=\skip126
\mdffootnoteboxheight=\skip127
\mdffootnoteboxdepth=\skip128
\mdffootnoteboxtotalheight=\skip129
\mdftotallinewidth=\skip130
\mdfboundingboxwidth=\skip131
\mdfboundingboxtotalwidth=\skip132
\mdfboundingboxheight=\skip133
\mdfboundingboxdepth=\skip134
\mdfboundingboxtotalheight=\skip135
\mdf@freevspace@length=\skip136
\mdf@horizontalwidthofbox@length=\skip137
\mdf@verticalmarginwhole@length=\skip138
\mdf@horizontalspaceofbox=\skip139
\mdfsubtitleheight=\skip140
\mdfsubsubtitleheight=\skip141
\c@mdfcountframes=\count282

****** mdframed patching \endmdf@trivlist

****** -- success******

\mdf@envdepth=\count283
\c@mdf@env@i=\count284
\c@mdf@env@ii=\count285
\c@mdf@zref@counter=\count286
Package zref Info: New property: mdf@pagevalue on input line 895.
) (c:/texlive/2023/texmf-dist/tex/latex/titlesec/titlesec.sty
Package: titlesec 2023/10/27 v2.16 Sectioning titles
\ttl@box=\box67
```

```
\beforetitleunit=\skip142
\aftertitleunit=\skip143
\ttl@plus=\dimen154
\ttl@minus=\dimen155
\ttl@toksa=\toks37
\titlewidth=\dimen156
\titlewidthlast=\dimen157
\titlewidthfirst=\dimen158
) (c:/texlive/2023/texmf-dist/tex/latex/koma-script/scrextend.sty
Package: scrextend 2023/07/07 v3.41 KOMA-Script package (extend other
classes w
ith features of KOMA-Script classes)
(c:/texlive/2023/texmf-dist/tex/latex/koma-script/scrkbase.sty
Package: scrkbase 2023/07/07 v3.41 KOMA-Script package (KOMA-Script-
dependent b
asics and keyval usage)
(c:/texlive/2023/texmf-dist/tex/latex/koma-script/scrbase.sty
Package: scrbase 2023/07/07 v3.41 KOMA-Script package (KOMA-Script-
independent
basics and keyval usage)
(c:/texlive/2023/texmf-dist/tex/latex/koma-script/scrlfile.sty
Package: scrlfile 2023/07/07 v3.41 KOMA-Script package (file load hooks)
(c:/texlive/2023/texmf-dist/tex/latex/koma-script/scrlfile-hook.sty
Package: scrlfile-hook 2023/07/07 v3.41 KOMA-Script package (using LaTeX
hooks)

(c:/texlive/2023/texmf-dist/tex/latex/koma-script/scrlogo.sty
Package: scrlogo 2023/07/07 v3.41 KOMA-Script package (logo)
)))
Applying: [2021/05/01] Usage of raw or classic option list on input line
252.
Already applied: [0000/00/00] Usage of raw or classic option list on
input line
 368.
))
Package scrextend Info: unexpected definition of `\@makefnmark'.
(scrextend)             Trying to patch it on input line 1762.
Package scrextend Info: patch seems to be successfull on input line 1762.
)

LaTeX Font Warning: Font shape `T1/cmr/m/n' in size <7.5> not available
(Font)              size <7> substituted on input line 72.

(c:/texlive/2023/texmf-dist/tex/latex/tools/calc.sty
Package: calc 2023/07/08 v4.3 Infix arithmetic (KKT,FJ)
\calc@Acount=\count287
\calc@Bcount=\count288
\calc@Adimen=\dimen159
\calc@Bdimen=\dimen160
\calc@Askip=\skip144
\calc@Bskip=\skip145
LaTeX Info: Redefining \setlength on input line 80.
LaTeX Info: Redefining \addtolength on input line 81.
\calc@Ccount=\count289
```

```
\calc@Cskip=\skip146
) (c:/texlive/2023/texmf-dist/tex/latex/geometry/geometry.sty
Package: geometry 2020/01/02 v5.9 Page Geometry
(c:/texlive/2023/texmf-dist/tex/generic/iftex/ifvtex.sty
Package: ifvtex 2019/10/25 v1.7 ifvtex legacy package. Use iftex instead.
)
\Gm@cnth=\count290
\Gm@cntv=\count291
\c@Gm@tempcnt=\count292
\Gm@bindingoffset=\dimen161
\Gm@wd@mp=\dimen162
\Gm@odd@mp=\dimen163
\Gm@even@mp=\dimen164
\Gm@layoutwidth=\dimen165
\Gm@layoutheight=\dimen166
\Gm@layouthoffset=\dimen167
\Gm@layoutvoffset=\dimen168
\Gm@dimlist=\toks38
) (c:/texlive/2023/texmf-dist/tex/latex/preprint/authblk.sty
Package: authblk 2001/02/27 1.3 (PWD)
\affilsep=\skip147
\@affilsep=\skip148
\c@Maxaffil=\count293
\c@authors=\count294
\c@affil=\count295
) (c:/texlive/2023/texmf-dist/tex/latex/footmisc/footmisc.sty
Package: footmisc 2023/07/05 v6.0f a miscellany of footnote facilities
\FN@temptoken=\toks39
\footnotemargin=\dimen169
\@outputbox@depth=\dimen170
Package footmisc Info: Declaring symbol style bringhurst on input line
696.
Package footmisc Info: Declaring symbol style chicago on input line 704.
Package footmisc Info: Declaring symbol style wiley on input line 713.
Package footmisc Info: Declaring symbol style lamport-robust on input
line 724.

Package footmisc Info: Declaring symbol style lamport* on input line 744.
Package footmisc Info: Declaring symbol style lamport*-robust on input
line 765
.
) (c:/texlive/2023/texmf-dist/tex/latex/fancyhdr/fancyhdr.sty
Package: fancyhdr 2022/11/09 v4.1 Extensive control of page headers and
footers

\f@nch@headwidth=\skip149
\f@nch@O@elh=\skip150
\f@nch@O@erh=\skip151
\f@nch@O@olh=\skip152
\f@nch@O@orh=\skip153
\f@nch@O@elf=\skip154
\f@nch@O@erf=\skip155
\f@nch@O@olf=\skip156
\f@nch@O@orf=\skip157
```

```
) (c:/texlive/2023/texmf-dist/tex/generic/alphalph/alphalph.sty
Package: alphalph 2019/12/09 v2.6 Convert numbers to letters (HO)
(c:/texlive/2023/texmf-dist/tex/generic/intcalc/intcalc.sty
Package: intcalc 2019/12/15 v1.3 Expandable calculations with integers
(HO)
))
\c@authorfn=\count296
(c:/texlive/2023/texmf-dist/tex/latex/abstract/abstract.sty
Package: abstract 2009/06/08 v1.2a configurable abstracts
\abstitleskip=\skip158
\absleftindent=\skip159
\absrightindent=\skip160
\absparindent=\skip161
\absparsep=\skip162
)
Package newfloat Info: New float `keypoints' with options
`placement=t!,name=kp
t' on input line 294.
\c@keypoints=\count297
Package newfloat Info: float type `keypoints'=8 on input line 294.
(c:/texlive/2023/texmf-dist/tex/latex/enumitem/enumitem.sty
Package: enumitem 2019/06/20 v3.9 Customized lists
\labelindent=\skip163
\enit@outerparindent=\dimen171
\enit@toks=\toks40
\enit@inbox=\box68
\enit@count@id=\count298
\enitdp@description=\count299
) (c:/texlive/2023/texmf-dist/tex/latex/quoting/quoting.sty
Package: quoting 2014/01/28 v0.1c Consolidated environment for displayed
text
\quo@toppartop=\skip164
) (c:/texlive/2023/texmf-dist/tex/latex/sttools/stfloats.sty
Package: stfloats 2017/03/27 v3.3 Improve float mechanism and
baselineskip sett
ings
\@dblbotnum=\count300
\c@dblbotnumber=\count301
) (c:/texlive/2023/texmf-dist/tex/latex/booktabs/booktabs.sty
Package: booktabs 2020/01/12 v1.61803398 Publication quality tables
\heavyrulewidth=\dimen172
\lightrulewidth=\dimen173
\cmidrulewidth=\dimen174
\belowrulesep=\dimen175
\belowbottomsep=\dimen176
\aboverulesep=\dimen177
\abovetopsep=\dimen178
\cmidrulesep=\dimen179
\cmidrulekern=\dimen180
\defaultaddspace=\dimen181
\@cmidla=\count302
\@cmidlb=\count303
\@aboverulesep=\dimen182
\@belowrulesep=\dimen183
```

```
\@thisruleclass=\count304
\@lastruleclass=\count305
\@thisrulewidth=\dimen184
) (c:/texlive/2023/texmf-dist/tex/latex/tools/tabularx.sty
Package: tabularx 2023/07/08 v2.11c `tabularx' package (DPC)
\TX@col@width=\dimen185
\TX@old@table=\dimen186
\TX@old@col=\dimen187
\TX@target=\dimen188
\TX@delta=\dimen189
\TX@cols=\count306
\TX@ftn=\toks41
)
\enitdp@tablenotes=\count307
(c:/texlive/2023/texmf-dist/tex/latex/caption/caption.sty
Package: caption 2023/08/05 v3.6o Customizing captions (AR)
(c:/texlive/2023/texmf-dist/tex/latex/caption/caption3.sty
Package: caption3 2023/07/31 v2.4d caption3 kernel (AR)
\caption@tempdima=\dimen190
\captionmargin=\dimen191
\caption@leftmargin=\dimen192
\caption@rightmargin=\dimen193
\caption@width=\dimen194
\caption@indent=\dimen195
\caption@parindent=\dimen196
\caption@hangindent=\dimen197
Package caption Info: Standard document class detected.
)
\c@caption@flags=\count308
\c@continuedfloat=\count309
Package caption Info: float package is loaded.
Package caption Info: rotating package is loaded.
Package caption Info: scrextend package is loaded.
\caption@addmargin@hsize=\dimen198
\caption@addmargin@linewidth=\dimen199
) (c:/texlive/2023/texmf-dist/tex/latex/natbib/natbib.sty
Package: natbib 2010/09/13 8.31b (PWD, AO)
\bibhang=\skip165
\bibsep=\skip166
LaTeX Info: Redefining \cite on input line 694.
\c@NAT@ctr=\count310
)) (c:/texlive/2023/texmf-dist/tex/latex/pgf/frontendlayer/tikz.sty
(c:/texlive
/2023/texmf-dist/tex/latex/pgf/basiclayer/pgf.sty (c:/texlive/2023/texmf-
dist/t
ex/latex/pgf/utilities/pgfrcs.sty (c:/texlive/2023/texmf-
dist/tex/generic/pgf/u
tilities/pgfutil-common.tex
\pgfutil@everybye=\toks42
\pgfutil@tempdima=\dimen256
\pgfutil@tempdimb=\dimen257
) (c:/texlive/2023/texmf-dist/tex/generic/pgf/utilities/pgfutil-latex.def
\pgfutil@abb=\box69
```

```
) (c:/texlive/2023/texmf-dist/tex/generic/pgf/utilities/pgfrcs.code.tex
(c:/tex
live/2023/texmf-dist/tex/generic/pgf/pgf.revision.tex)
Package: pgfrcs 2023-01-15 v3.1.10 (3.1.10)
))
Package: pgf 2023-01-15 v3.1.10 (3.1.10)
(c:/texlive/2023/texmf-dist/tex/latex/pgf/basiclayer/pgfcore.sty
(c:/texlive/20
23/texmf-dist/tex/latex/pgf/systemlayer/pgfsys.sty
(c:/texlive/2023/texmf-dist/
tex/generic/pgf/systemlayer/pgfsys.code.tex
Package: pgfsys 2023-01-15 v3.1.10 (3.1.10)
(c:/texlive/2023/texmf-dist/tex/generic/pgf/utilities/pgfkeys.code.tex
\pgfkeys@pathtoks=\toks43
\pgfkeys@temptoks=\toks44

(c:/texlive/2023/texmf-
dist/tex/generic/pgf/utilities/pgfkeyslibraryfiltered.co
de.tex
\pgfkeys@tmptoks=\toks45
))
\pgf@x=\dimen258
\pgf@y=\dimen259
\pgf@xa=\dimen260
\pgf@ya=\dimen261
\pgf@xb=\dimen262
\pgf@yb=\dimen263
\pgf@xc=\dimen264
\pgf@yc=\dimen265
\pgf@xd=\dimen266
\pgf@yd=\dimen267
\w@pgf@writea=\write3
\r@pgf@reada=\read2
\c@pgf@counta=\count311
\c@pgf@countb=\count312
\c@pgf@countc=\count313
\c@pgf@countd=\count314
\t@pgf@toka=\toks46
\t@pgf@tokb=\toks47
\t@pgf@tokc=\toks48
\pgf@sys@id@count=\count315
(c:/texlive/2023/texmf-dist/tex/generic/pgf/systemlayer/pgf.cfg
File: pgf.cfg 2023-01-15 v3.1.10 (3.1.10)
)
Driver file for pgf: pgfsys-pdftex.def
(c:/texlive/2023/texmf-dist/tex/generic/pgf/systemlayer/pgfsys-pdftex.def
File: pgfsys-pdftex.def 2023-01-15 v3.1.10 (3.1.10)
(c:/texlive/2023/texmf-dist/tex/generic/pgf/systemlayer/pgfsys-common-
pdf.def
File: pgfsys-common-pdf.def 2023-01-15 v3.1.10 (3.1.10)
)))
(c:/texlive/2023/texmf-
dist/tex/generic/pgf/systemlayer/pgfsyssoftpath.code.tex
File: pgfsyssoftpath.code.tex 2023-01-15 v3.1.10 (3.1.10)
```

```
\pgfsyssoftpath@smallbuffer@items=\count316
\pgfsyssoftpath@bigbuffer@items=\count317
)
(c:/texlive/2023/texmf-
dist/tex/generic/pgf/systemlayer/pgfsysprotocol.code.tex
File: pgfsysprotocol.code.tex 2023-01-15 v3.1.10 (3.1.10)
)) (c:/texlive/2023/texmf-
dist/tex/generic/pgf/basiclayer/pgfcore.code.tex
Package: pgfcore 2023-01-15 v3.1.10 (3.1.10)
(c:/texlive/2023/texmf-dist/tex/generic/pgf/math/pgfmath.code.tex
(c:/texlive/2
023/texmf-dist/tex/generic/pgf/math/pgfmathutil.code.tex)
(c:/texlive/2023/texm
f-dist/tex/generic/pgf/math/pgfmathparser.code.tex
\pgfmath@dimen=\dimen268
\pgfmath@count=\count318
\pgfmath@box=\box70
\pgfmath@toks=\toks49
\pgfmath@stack@operand=\toks50
\pgfmath@stack@operation=\toks51
) (c:/texlive/2023/texmf-
dist/tex/generic/pgf/math/pgfmathfunctions.code.tex)
(c:/texlive/2023/texmf-
dist/tex/generic/pgf/math/pgfmathfunctions.basic.code.te
x)
(c:/texlive/2023/texmf-
dist/tex/generic/pgf/math/pgfmathfunctions.trigonometric
.code.tex)
(c:/texlive/2023/texmf-
dist/tex/generic/pgf/math/pgfmathfunctions.random.code.t
ex)
(c:/texlive/2023/texmf-
dist/tex/generic/pgf/math/pgfmathfunctions.comparison.co
de.tex)
(c:/texlive/2023/texmf-
dist/tex/generic/pgf/math/pgfmathfunctions.base.code.tex
)
(c:/texlive/2023/texmf-
dist/tex/generic/pgf/math/pgfmathfunctions.round.code.te
x)
(c:/texlive/2023/texmf-
dist/tex/generic/pgf/math/pgfmathfunctions.misc.code.tex
)
(c:/texlive/2023/texmf-
dist/tex/generic/pgf/math/pgfmathfunctions.integerarithm
etics.code.tex) (c:/texlive/2023/texmf-
dist/tex/generic/pgf/math/pgfmathcalc.co
de.tex) (c:/texlive/2023/texmf-
dist/tex/generic/pgf/math/pgfmathfloat.code.tex
\c@pgfmathroundto@lastzeros=\count319
)) (c:/texlive/2023/texmf-dist/tex/generic/pgf/math/pgfint.code.tex)
(c:/texliv
e/2023/texmf-dist/tex/generic/pgf/basiclayer/pgfcorepoints.code.tex
File: pgfcorepoints.code.tex 2023-01-15 v3.1.10 (3.1.10)
```

```
\pgf@picminx=\dimen269
\pgf@picmaxx=\dimen270
\pgf@picminy=\dimen271
\pgf@picmaxy=\dimen272
\pgf@pathminx=\dimen273
\pgf@pathmaxx=\dimen274
\pgf@pathminy=\dimen275
\pgf@pathmaxy=\dimen276
\pgf@xx=\dimen277
\pgf@xy=\dimen278
\pgf@yx=\dimen279
\pgf@yy=\dimen280
\pgf@zx=\dimen281
\pgf@zy=\dimen282
)
(c:/texlive/2023/texmf-
dist/tex/generic/pgf/basiclayer/pgfcorepathconstruct.cod
e.tex
File: pgfcorepathconstruct.code.tex 2023-01-15 v3.1.10 (3.1.10)
\pgf@path@lastx=\dimen283
\pgf@path@lasty=\dimen284
)
(c:/texlive/2023/texmf-
dist/tex/generic/pgf/basiclayer/pgfcorepathusage.code.te
x
File: pgfcorepathusage.code.tex 2023-01-15 v3.1.10 (3.1.10)
\pgf@shorten@end@additional=\dimen285
\pgf@shorten@start@additional=\dimen286
) (c:/texlive/2023/texmf-
dist/tex/generic/pgf/basiclayer/pgfcorescopes.code.tex
File: pgfcorescopes.code.tex 2023-01-15 v3.1.10 (3.1.10)
\pgfpic=\box71
\pgf@hbox=\box72
\pgf@layerbox@main=\box73
\pgf@picture@serial@count=\count320
)
(c:/texlive/2023/texmf-
dist/tex/generic/pgf/basiclayer/pgfcoregraphicstate.code
.tex
File: pgfcoregraphicstate.code.tex 2023-01-15 v3.1.10 (3.1.10)
\pgflinewidth=\dimen287
)
(c:/texlive/2023/texmf-
dist/tex/generic/pgf/basiclayer/pgfcoretransformations.c
ode.tex
File: pgfcoretransformations.code.tex 2023-01-15 v3.1.10 (3.1.10)
\pgf@pt@x=\dimen288
\pgf@pt@y=\dimen289
\pgf@pt@temp=\dimen290
) (c:/texlive/2023/texmf-
dist/tex/generic/pgf/basiclayer/pgfcorequick.code.tex
File: pgfcorequick.code.tex 2023-01-15 v3.1.10 (3.1.10)
) (c:/texlive/2023/texmf-
dist/tex/generic/pgf/basiclayer/pgfcoreobjects.code.te
```

```
x
File: pgfcoreobjects.code.tex 2023-01-15 v3.1.10 (3.1.10)
)
(c:/texlive/2023/texmf-
dist/tex/generic/pgf/basiclayer/pgfcorepathprocessing.co
de.tex
File: pgfcorepathprocessing.code.tex 2023-01-15 v3.1.10 (3.1.10)
) (c:/texlive/2023/texmf-
dist/tex/generic/pgf/basiclayer/pgfcorearrows.code.tex
File: pgfcorearrows.code.tex 2023-01-15 v3.1.10 (3.1.10)
\pgfarrowsep=\dimen291
) (c:/texlive/2023/texmf-
dist/tex/generic/pgf/basiclayer/pgfcoreshade.code.tex
File: pgfcoreshade.code.tex 2023-01-15 v3.1.10 (3.1.10)
\pgf@max=\dimen292
\pgf@sys@shading@range@num=\count321
\pgf@shadingcount=\count322
) (c:/texlive/2023/texmf-
dist/tex/generic/pgf/basiclayer/pgfcoreimage.code.tex
File: pgfcoreimage.code.tex 2023-01-15 v3.1.10 (3.1.10)
)
(c:/texlive/2023/texmf-
dist/tex/generic/pgf/basiclayer/pgfcoreexternal.code.tex
File: pgfcoreexternal.code.tex 2023-01-15 v3.1.10 (3.1.10)
\pgfexternal@startupbox=\box74
) (c:/texlive/2023/texmf-
dist/tex/generic/pgf/basiclayer/pgfcorelayers.code.tex
File: pgfcorelayers.code.tex 2023-01-15 v3.1.10 (3.1.10)
)
(c:/texlive/2023/texmf-
dist/tex/generic/pgf/basiclayer/pgfcoretransparency.code
.tex
File: pgfcoretransparency.code.tex 2023-01-15 v3.1.10 (3.1.10)
)
(c:/texlive/2023/texmf-
dist/tex/generic/pgf/basiclayer/pgfcorepatterns.code.tex
File: pgfcorepatterns.code.tex 2023-01-15 v3.1.10 (3.1.10)
) (c:/texlive/2023/texmf-
dist/tex/generic/pgf/basiclayer/pgfcorerdf.code.tex
File: pgfcorerdf.code.tex 2023-01-15 v3.1.10 (3.1.10)
))) (c:/texlive/2023/texmf-
dist/tex/generic/pgf/modules/pgfmoduleshapes.code.te
x
File: pgfmoduleshapes.code.tex 2023-01-15 v3.1.10 (3.1.10)
\pgfnodeparttextbox=\box75
) (c:/texlive/2023/texmf-
dist/tex/generic/pgf/modules/pgfmoduleplot.code.tex
File: pgfmoduleplot.code.tex 2023-01-15 v3.1.10 (3.1.10)
)
(c:/texlive/2023/texmf-dist/tex/latex/pgf/compatibility/pgfcomp-version-
0-65.st
y
Package: pgfcomp-version-0-65 2023-01-15 v3.1.10 (3.1.10)
\pgf@nodesepstart=\dimen293
```

```
\pgf@nodesepend=\dimen294
)
(c:/texlive/2023/texmf-dist/tex/latex/pgf/compatibility/pgfcomp-version-
1-18.st
y
Package: pgfcomp-version-1-18 2023-01-15 v3.1.10 (3.1.10)
)) (c:/texlive/2023/texmf-dist/tex/latex/pgf/utilities/pgffor.sty
(c:/texlive/2
023/texmf-dist/tex/latex/pgf/utilities/pgfkeys.sty
(c:/texlive/2023/texmf-dist/
tex/generic/pgf/utilities/pgfkeys.code.tex)) (c:/texlive/2023/texmf-
dist/tex/la
tex/pgf/math/pgfmath.sty (c:/texlive/2023/texmf-
dist/tex/generic/pgf/math/pgfma
th.code.tex)) (c:/texlive/2023/texmf-
dist/tex/generic/pgf/utilities/pgffor.code
.tex
Package: pgffor 2023-01-15 v3.1.10 (3.1.10)
\pgffor@iter=\dimen295
\pgffor@skip=\dimen296
\pgffor@stack=\toks52
\pgffor@toks=\toks53
)) (c:/texlive/2023/texmf-
dist/tex/generic/pgf/frontendlayer/tikz/tikz.code.tex
Package: tikz 2023-01-15 v3.1.10 (3.1.10)

(c:/texlive/2023/texmf-
dist/tex/generic/pgf/libraries/pgflibraryplothandlers.co
de.tex
File: pgflibraryplothandlers.code.tex 2023-01-15 v3.1.10 (3.1.10)
\pgf@plot@mark@count=\count323
\pgfplotmarksize=\dimen297
)
\tikz@lastx=\dimen298
\tikz@lasty=\dimen299
\tikz@lastxsaved=\dimen300
\tikz@lastysaved=\dimen301
\tikz@lastmovetox=\dimen302
\tikz@lastmovetoy=\dimen303
\tikzleveldistance=\dimen304
\tikzsiblingdistance=\dimen305
\tikz@figbox=\box76
\tikz@figbox@bg=\box77
\tikz@tempbox=\box78
\tikz@tempbox@bg=\box79
\tikztreelevel=\count324
\tikznumberofchildren=\count325
\tikznumberofcurrentchild=\count326
\tikz@fig@count=\count327
(c:/texlive/2023/texmf-
dist/tex/generic/pgf/modules/pgfmodulematrix.code.tex
File: pgfmodulematrix.code.tex 2023-01-15 v3.1.10 (3.1.10)
\pgfmatrixcurrentrow=\count328
\pgfmatrixcurrentcolumn=\count329
```

```
\pgf@matrix@numberofcolumns=\count330
)
\tikz@expandcount=\count331

(c:/texlive/2023/texmf-
dist/tex/generic/pgf/frontendlayer/tikz/libraries/tikzli
brarytopaths.code.tex
File: tikzlibrarytopaths.code.tex 2023-01-15 v3.1.10 (3.1.10)
))) (c:/texlive/2023/texmf-dist/tex/latex/amsmath/amsmath.sty
Package: amsmath 2023/05/13 v2.17o AMS math features
\@mathmargin=\skip167
For additional information on amsmath, use the `?' option.
(c:/texlive/2023/texmf-dist/tex/latex/amsmath/amstext.sty
Package: amstext 2021/08/26 v2.01 AMS text
(c:/texlive/2023/texmf-dist/tex/latex/amsmath/amsgen.sty
File: amsgen.sty 1999/11/30 v2.0 generic functions
\@emptytoks=\toks54
\ex@=\dimen306
)) (c:/texlive/2023/texmf-dist/tex/latex/amsmath/amsbsy.sty
Package: amsbsy 1999/11/29 v1.2d Bold Symbols
\pmbraise@=\dimen307
) (c:/texlive/2023/texmf-dist/tex/latex/amsmath/amsopn.sty
Package: amsopn 2022/04/08 v2.04 operator names
)
\inf@bad=\count332
LaTeX Info: Redefining \frac on input line 234.
\uproot@=\count333
\leftroot@=\count334
LaTeX Info: Redefining \overline on input line 399.
LaTeX Info: Redefining \colon on input line 410.
\classnum@=\count335
\DOTSCASE@=\count336
LaTeX Info: Redefining \ldots on input line 496.
LaTeX Info: Redefining \dots on input line 499.
LaTeX Info: Redefining \cdots on input line 620.
\Mathstrutbox@=\box80
\strutbox@=\box81
LaTeX Info: Redefining \big on input line 722.
LaTeX Info: Redefining \Big on input line 723.
LaTeX Info: Redefining \bigg on input line 724.
LaTeX Info: Redefining \Bigg on input line 725.
\big@size=\dimen308
LaTeX Font Info:    Redeclaring font encoding OML on input line 743.
LaTeX Font Info:    Redeclaring font encoding OMS on input line 744.
\macc@depth=\count337
LaTeX Info: Redefining \bmod on input line 905.
LaTeX Info: Redefining \pmod on input line 910.
LaTeX Info: Redefining \smash on input line 940.
LaTeX Info: Redefining \relbar on input line 970.
LaTeX Info: Redefining \Relbar on input line 971.
\c@MaxMatrixCols=\count338
\dotsspace@=\muskip20
\c@parentequation=\count339
\dspbrk@lvl=\count340
```

```
\tag@help=\toks55
\row@=\count341
\column@=\count342
\maxfields@=\count343
\andhelp@=\toks56
\eqnshift@=\dimen309
\alignsep@=\dimen310
\tagshift@=\dimen311
\tagwidth@=\dimen312
\totwidth@=\dimen313
\lineht@=\dimen314
\@envbody=\toks57
\multlinegap=\skip168
\multlinetaggap=\skip169
\mathdisplay@stack=\toks58
LaTeX Info: Redefining \[ on input line 2953.
LaTeX Info: Redefining \] on input line 2954.
) (c:/texlive/2023/texmf-dist/tex/latex/orcidlink/orcidlink.sty
Package: orcidlink 2023/12/30 v1.0.5 Linked ORCiD logo macro package
(c:/texlive/2023/texmf-dist/tex/latex/hyperref/hyperref.sty
Package: hyperref 2024-01-20 v7.01h Hypertext links for LaTeX
(c:/texlive/2023/texmf-dist/tex/generic/pdfescape/pdfescape.sty
Package: pdfescape 2019/12/09 v1.15 Implements pdfTeX's escape features
(HO)
) (c:/texlive/2023/texmf-dist/tex/latex/hycolor/hycolor.sty
Package: hycolor 2020-01-27 v1.10 Color options for hyperref/bookmark
(HO)
) (c:/texlive/2023/texmf-dist/tex/latex/hyperref/nameref.sty
Package: nameref 2023-11-26 v2.56 Cross-referencing by name of section
(c:/texlive/2023/texmf-dist/tex/latex/refcount/refcount.sty
Package: refcount 2019/12/15 v3.6 Data extraction from label references
(HO)
) (c:/texlive/2023/texmf-
dist/tex/generic/gettitlestring/gettitlestring.sty
Package: gettitlestring 2019/12/15 v1.6 Cleanup title references (HO)
)
\c@section@level=\count344
)
\@linkdim=\dimen315
\Hy@linkcounter=\count345
\Hy@pagecounter=\count346
(c:/texlive/2023/texmf-dist/tex/latex/hyperref/pd1enc.def
File: pd1enc.def 2024-01-20 v7.01h Hyperref: PDFDocEncoding definition
(HO)
Now handling font encoding PD1 ...
... no UTF-8 mapping file for font encoding PD1
)
\Hy@SavedSpaceFactor=\count347
(c:/texlive/2023/texmf-dist/tex/latex/hyperref/puenc.def
File: puenc.def 2024-01-20 v7.01h Hyperref: PDF Unicode definition (HO)
Now handling font encoding PU ...
... no UTF-8 mapping file for font encoding PU
)
Package hyperref Info: Hyper figures OFF on input line 4179.
```

```
Package hyperref Info: Link nesting OFF on input line 4184.
Package hyperref Info: Hyper index ON on input line 4187.
Package hyperref Info: Plain pages OFF on input line 4194.
Package hyperref Info: Backreferencing OFF on input line 4199.
Package hyperref Info: Implicit mode ON; LaTeX internals redefined.
Package hyperref Info: Bookmarks ON on input line 4446.
\c@Hy@tempcnt=\count348
LaTeX Info: Redefining \url on input line 4784.
\XeTeXLinkMargin=\dimen316
(c:/texlive/2023/texmf-dist/tex/generic/bitset/bitset.sty
Package: bitset 2019/12/09 v1.3 Handle bit-vector datatype (HO)
(c:/texlive/2023/texmf-dist/tex/generic/bigintcalc/bigintcalc.sty
Package: bigintcalc 2019/12/15 v1.5 Expandable calculations on big
integers (HO
)
))
\Fld@menulength=\count349
\Field@Width=\dimen317
\Fld@charsize=\dimen318
Package hyperref Info: Hyper figures OFF on input line 6063.
Package hyperref Info: Link nesting OFF on input line 6068.
Package hyperref Info: Hyper index ON on input line 6071.
Package hyperref Info: backreferencing OFF on input line 6078.
Package hyperref Info: Link coloring OFF on input line 6083.
Package hyperref Info: Link coloring with OCG OFF on input line 6088.
Package hyperref Info: PDF/A mode OFF on input line 6093.
(c:/texlive/2023/texmf-dist/tex/latex/base/atbegshi-ltx.sty
Package: atbegshi-ltx 2021/01/10 v1.0c Emulation of the original atbegshi
package with kernel methods
)
\Hy@abspage=\count350
\c@Item=\count351
\c@Hfootnote=\count352
)
Package hyperref Info: Driver (autodetected): hpdftex.
(c:/texlive/2023/texmf-dist/tex/latex/hyperref/hpdftex.def
File: hpdftex.def 2024-01-20 v7.01h Hyperref driver for pdfTeX
(c:/texlive/2023/texmf-dist/tex/latex/base/atveryend-ltx.sty
Package: atveryend-ltx 2020/08/19 v1.0a Emulation of the original
atveryend pac
kage
with kernel methods
)
\HyAnn@Count=\count353
\Fld@listcount=\count354
\c@bookmark@seq@number=\count355
(c:/texlive/2023/texmf-dist/tex/latex/rerunfilecheck/rerunfilecheck.sty
Package: rerunfilecheck 2022-07-10 v1.10 Rerun checks for auxiliary files
(HO)
(c:/texlive/2023/texmf-dist/tex/generic/uniquecounter/uniquecounter.sty
Package: uniquecounter 2019/12/15 v1.4 Provide unlimited unique counter
(HO)
)
```

```
Package uniquecounter Info: New unique counter `rerunfilecheck' on input
line 2
85.
)
\Hy@SectionHShift=\skip170
)
(c:/texlive/2023/texmf-
dist/tex/generic/pgf/frontendlayer/tikz/libraries/tikzli
brarysvg.path.code.tex
File: tikzlibrarysvg.path.code.tex 2023-01-15 v3.1.10 (3.1.10)

(c:/texlive/2023/texmf-
dist/tex/generic/pgf/libraries/pgflibrarysvg.path.code.t
ex
File: pgflibrarysvg.path.code.tex 2023-01-15 v3.1.10 (3.1.10)
(c:/texlive/2023/texmf-
dist/tex/generic/pgf/modules/pgfmoduleparser.code.tex
File: pgfmoduleparser.code.tex 2023-01-15 v3.1.10 (3.1.10)
\pgfparserdef@arg@count=\count356
)
\pgf@lib@svg@last@x=\dimen319
\pgf@lib@svg@last@y=\dimen320
\pgf@lib@svg@last@c@x=\dimen321
\pgf@lib@svg@last@c@y=\dimen322
\pgf@lib@svg@count=\count357
\pgf@lib@svg@max@num=\count358
))
\@curXheight=\skip171
) (c:/texlive/2023/texmf-dist/tex/generic/soul/soul.sty
Package: soul 2023-06-14 v3.1 Permit use of UTF-8 characters in soul (HO)
(c:/texlive/2023/texmf-dist/tex/generic/soul/soul-ori.sty
Package: soul-ori 2023-06-14 v3.1 letterspacing/underlining (mf)
\SOUL@word=\toks59
\SOUL@lasttoken=\toks60
\SOUL@syllable=\toks61
\SOUL@cmds=\toks62
\SOUL@buffer=\toks63
\SOUL@token=\toks64
\SOUL@syllgoal=\dimen323
\SOUL@syllwidth=\dimen324
\SOUL@charkern=\dimen325
\SOUL@hyphkern=\dimen326
\SOUL@dimen=\dimen327
\SOUL@dimeni=\dimen328
\SOUL@minus=\count359
\SOUL@comma=\count360
\SOUL@apo=\count361
\SOUL@grave=\count362
\SOUL@spaceskip=\skip172
\SOUL@ttwidth=\dimen329
\SOUL@uldp=\dimen330
\SOUL@ulht=\dimen331
)) (c:/texlive/2023/texmf-dist/tex/latex/siunitx/siunitx.sty
Package: siunitx 2024-02-15 v3.3.12 A comprehensive (SI) units package
```

```
\l__siunitx_number_uncert_offset_int=\count363
\l__siunitx_number_exponent_fixed_int=\count364
\l__siunitx_number_min_decimal_int=\count365
\l__siunitx_number_min_integer_int=\count366
\l__siunitx_number_round_precision_int=\count367
\l__siunitx_number_lower_threshold_int=\count368
\l__siunitx_number_upper_threshold_int=\count369
\l__siunitx_number_group_first_int=\count370
\l__siunitx_number_group_size_int=\count371
\l__siunitx_number_group_minimum_int=\count372
\l__siunitx_angle_tmp_dim=\dimen332
\l__siunitx_angle_marker_box=\box82
\l__siunitx_angle_unit_box=\box83
\l__siunitx_compound_count_int=\count373
(c:/texlive/2023/texmf-dist/tex/latex/translations/translations.sty
Package: translations 2022/02/05 v1.12 internationalization of LaTeX2e
packages
 (CN)
)
\l__siunitx_table_tmp_box=\box84
\l__siunitx_table_tmp_dim=\dimen333
\l__siunitx_table_column_width_dim=\dimen334
\l__siunitx_table_integer_box=\box85
\l__siunitx_table_decimal_box=\box86
\l__siunitx_table_uncert_box=\box87
\l__siunitx_table_before_box=\box88
\l__siunitx_table_after_box=\box89
\l__siunitx_table_before_dim=\dimen335
\l__siunitx_table_carry_dim=\dimen336
\l__siunitx_unit_tmp_int=\count374
\l__siunitx_unit_position_int=\count375
\l__siunitx_unit_total_int=\count376
) (c:/texlive/2023/texmf-dist/tex/latex/amsfonts/amssymb.sty
Package: amssymb 2013/01/14 v3.01 AMS font symbols
(c:/texlive/2023/texmf-dist/tex/latex/amsfonts/amsfonts.sty
Package: amsfonts 2013/01/14 v3.01 Basic AMSFonts support
\symAMSa=\mathgroup10
\symAMSb=\mathgroup11
LaTeX Font Info:    Redeclaring math symbol \hbar on input line 98.
LaTeX Info: Redefining \frak on input line 111.
)) (c:/texlive/2023/texmf-dist/tex/latex/lineno/lineno.sty
Package: lineno 2023/05/20 line numbers on paragraphs v5.3
\linenopenalty=\count377
\output=\toks65
\linenoprevgraf=\count378
\linenumbersep=\dimen337
\linenumberwidth=\dimen338
\c@linenumber=\count379
\c@pagewiselinenumber=\count380
\c@LN@truepage=\count381
\c@internallinenumber=\count382
\c@internallinenumbers=\count383
\quotelinenumbersep=\dimen339
\bframerule=\dimen340
```

```
\bframesep=\dimen341
\bframebox=\box90
\linenoamsmath@ams@eqpen=\count384
LaTeX Info: Redefining \\ on input line 3180.
)

! LaTeX Error: Option clash for package hyperref.

See the LaTeX manual or LaTeX Companion for explanation.
Type  H <return>  for immediate help.
 ...

l.67 \begin{document}

The package hyperref has already been loaded with options:
  []
There has now been an attempt to load it with options
  [colorlinks,allcolors=black,urlcolor=blue]
Adding the global options:
  ,colorlinks,allcolors=black,urlcolor=blue
to your \documentclass declaration may fix this.
Try typing  <return>  to proceed.

Package translations Info: No language package found. I am going to use
`englis
h' as default language. on input line 67.
LaTeX Font Info:    Trying to load font information for T1+Merriwthr-OsF
on inp
ut line 67.
(c:/texlive/2023/texmf-dist/tex/latex/merriweather/T1Merriwthr-OsF.fd
File: T1Merriwthr-OsF.fd 2020/08/30 (autoinst) Font definitions for
T1/Merriwth
r-OsF.
)
LaTeX Font Info:    Font shape `T1/Merriwthr-OsF/m/n' will be
(Font)              scaled to size 7.5pt on input line 67.
(./manuscript_file.aux)
\openout1 = `manuscript_file.aux'.

LaTeX Font Info:    Checking defaults for OML/cmm/m/it on input line 67.
LaTeX Font Info:    ... okay on input line 67.
LaTeX Font Info:    Checking defaults for OMS/cmsy/m/n on input line 67.
LaTeX Font Info:    ... okay on input line 67.
LaTeX Font Info:    Checking defaults for OT1/cmr/m/n on input line 67.
LaTeX Font Info:    ... okay on input line 67.
LaTeX Font Info:    Checking defaults for T1/cmr/m/n on input line 67.
LaTeX Font Info:    ... okay on input line 67.
LaTeX Font Info:    Checking defaults for TS1/cmr/m/n on input line 67.
LaTeX Font Info:    ... okay on input line 67.
LaTeX Font Info:    Checking defaults for OMX/cmex/m/n on input line 67.
LaTeX Font Info:    ... okay on input line 67.
LaTeX Font Info:    Checking defaults for U/cmr/m/n on input line 67.
LaTeX Font Info:    ... okay on input line 67.
LaTeX Font Info:    Checking defaults for PD1/pdf/m/n on input line 67.
```

```
LaTeX Font Info:    ... okay on input line 67.
LaTeX Font Info:    Checking defaults for PU/pdf/m/n on input line 67.
LaTeX Font Info:    ... okay on input line 67.
LaTeX Info: Redefining \microtypecontext on input line 67.
Package microtype Info: Applying patch `item' on input line 67.
Package microtype Info: Applying patch `toc' on input line 67.
Package microtype Info: Applying patch `eqnum' on input line 67.
Package microtype Info: Applying patch `footnote' on input line 67.
Package microtype Info: Applying patch `verbatim' on input line 67.
Package microtype Info: Generating PDF output.
Package microtype Info: Character protrusion enabled (level 2).
Package microtype Info: Using default protrusion set `alltext'.
Package microtype Info: Automatic font expansion enabled (level 2),
(microtype)             stretch: 20, shrink: 20, step: 1, non-selected.
Package microtype Info: Using default expansion set `alltext-nott'.
LaTeX Info: Redefining \showhyphens on input line 67.
Package microtype Info: No adjustment of tracking.
Package microtype Info: No adjustment of interword spacing.
Package microtype Info: No adjustment of character kerning.
Package microtype Info: Loading generic protrusion settings for font
family
(microtype)             `Merriwthr-OsF' (encoding: T1).
(microtype)             For optimal results, create family-specific
settings.
(microtype)             See the microtype manual for details.
LaTeX Font Info:    Redeclaring symbol font `operators' on input line 67.
LaTeX Font Info:    Encoding `OT1' has changed to `T1' for symbol font
(Font)              `operators' in the math version `normal' on input
line 67.
LaTeX Font Info:    Overwriting symbol font `operators' in version
`normal'
(Font)                  OT1/cmr/m/n --> T1/Merriwthr-OsF/m/up on input
line 67.

LaTeX Font Info:    Encoding `OT1' has changed to `T1' for symbol font
(Font)              `operators' in the math version `bold' on input line
67.
LaTeX Font Info:    Overwriting symbol font `operators' in version `bold'
(Font)                  OT1/cmr/bx/n --> T1/Merriwthr-OsF/m/up on input
line 67
.
LaTeX Font Info:    Overwriting symbol font `operators' in version `bold'
(Font)                  T1/Merriwthr-OsF/m/up --> T1/Merriwthr-OsF/b/up
on inpu
t line 67.
LaTeX Font Info:    Redeclaring math alphabet \mathbf on input line 67.
LaTeX Font Info:    Overwriting math alphabet `\mathbf' in version
`normal'
(Font)                  OT1/cmr/bx/n --> T1/Merriwthr-OsF/b/up on input
line 67
.
LaTeX Font Info:    Overwriting math alphabet `\mathbf' in version `bold'
(Font)                  OT1/cmr/bx/n --> T1/Merriwthr-OsF/b/up on input
line 67
```

```
.
LaTeX Font Info:    Redeclaring math alphabet \mathsf on input line 67.
LaTeX Font Info:    Overwriting math alphabet `\mathsf' in version
`normal'
(Font)                  OT1/cmss/m/n --> T1/MerriwthrSans-OsF/m/up on
input lin
e 67.
LaTeX Font Info:    Overwriting math alphabet `\mathsf' in version `bold'
(Font)                  OT1/cmss/bx/n --> T1/MerriwthrSans-OsF/m/up on
input li
ne 67.
LaTeX Font Info:    Redeclaring math alphabet \mathit on input line 67.
LaTeX Font Info:    Overwriting math alphabet `\mathit' in version
`normal'
(Font)                  OT1/cmr/m/it --> T1/Merriwthr-OsF/m/it on input
line 67
.
LaTeX Font Info:    Overwriting math alphabet `\mathit' in version `bold'
(Font)                  OT1/cmr/bx/it --> T1/Merriwthr-OsF/m/it on input
line 6
7.
LaTeX Font Info:    Redeclaring math alphabet \mathtt on input line 67.
LaTeX Font Info:    Overwriting math alphabet `\mathtt' in version
`normal'
(Font)                  OT1/cmtt/m/n --> T1/lmtt/m/up on input line 67.
LaTeX Font Info:    Overwriting math alphabet `\mathtt' in version `bold'
(Font)                  OT1/cmtt/m/n --> T1/lmtt/m/up on input line 67.
LaTeX Font Info:    Overwriting math alphabet `\mathsf' in version `bold'
(Font)                  T1/MerriwthrSans-OsF/m/up --> T1/MerriwthrSans-
OsF/b/up
 on input line 67.
LaTeX Font Info:    Overwriting math alphabet `\mathit' in version `bold'
(Font)                  T1/Merriwthr-OsF/m/it --> T1/Merriwthr-OsF/b/it
on inpu
t line 67.
\c@mv@tabular=\count385
\c@mv@boldtabular=\count386
(c:/texlive/2023/texmf-dist/tex/context/base/mkii/supp-pdf.mkii
[Loading MPS to PDF converter (version 2006.09.02).]
\scratchcounter=\count387
\scratchdimen=\dimen342
\scratchbox=\box91
\nofMPsegments=\count388
\nofMParguments=\count389
\everyMPshowfont=\toks66
\MPscratchCnt=\count390
\MPscratchDim=\dimen343
\MPnumerator=\count391
\makeMPintoPDFobject=\count392
\everyMPtoPDFconversion=\toks67
) (c:/texlive/2023/texmf-dist/tex/latex/epstopdf-pkg/epstopdf-base.sty
Package: epstopdf-base 2020-01-24 v2.11 Base part for package epstopdf
Package epstopdf-base Info: Redefining graphics rule for `.eps' on input
line 4
```

```
85.
(c:/texlive/2023/texmf-dist/tex/latex/latexconfig/epstopdf-sys.cfg
File: epstopdf-sys.cfg 2010/07/13 v1.3 Configuration of (r)epstopdf for
TeX Liv
e
))
Package newfloat Info: `float' package detected.
*geometry* driver: auto-detecting
*geometry* detected driver: pdftex
*geometry* verbose mode - [ preamble ] result:
* driver: pdftex
* paper: a4paper
* layout: <same size as paper>
* layoutoffset:(h,v)=(0.0pt,0.0pt)
* modes: includefoot twoside
* h-part:(L,W,R)=(54.64pt, 488.22787pt, 54.64pt)
* v-part:(T,H,B)=(66.0pt, 745.04684pt, 34.0pt)
* \paperwidth=597.50787pt
* \paperheight=845.04684pt
* \textwidth=488.22787pt
* \textheight=715.04684pt
* \oddsidemargin=-17.62999pt
* \evensidemargin=-17.62999pt
* \topmargin=-47.76999pt
* \headheight=17.5pt
* \headsep=24.0pt
* \topskip=10.0pt
* \footskip=30.0pt
* \marginparwidth=48.0pt
* \marginparsep=10.0pt
* \columnsep=18.0pt
* \skip\footins=22.0pt plus 2.0pt
* \hoffset=0.0pt
* \voffset=0.0pt
* \mag=1000
* \@twocolumntrue
* \@twosidetrue
* \@mparswitchtrue
* \@reversemarginfalse
* (1in=72.27pt=25.4mm, 1cm=28.453pt)

Package caption Info: Begin \AtBeginDocument code.
Package caption Info: hyperref package is loaded.
Package caption Info: End \AtBeginDocument code.
Package hyperref Info: Link coloring OFF on input line 67.
(./manuscript_file.out) (./manuscript_file.out)
\@outlinefile=\write4
\openout4 = `manuscript_file.out'.


(c:/texlive/2023/texmf-dist/tex/latex/translations/translations-basic-
dictionar
y-english.trsl
```

```
File: translations-basic-dictionary-english.trsl (english translation
file `tra
nslations-basic-dictionary')
)
Package translations Info: loading dictionary `translations-basic-
dictionary' f
or `english'. on input line 67.
\@gscitedetails=\box92
\@gscitedetailsheight=\skip173
\@gsheadbox=\box93
\@gsheadboxheight=\skip174
LaTeX Font Info:    Font shape `T1/Merriwthr-OsF/b/n' will be
(Font)              scaled to size 6.5pt on input line 67.
LaTeX Font Info:    Calculating math sizes for size <7.5> on input line
67.

LaTeX Font Warning: Font shape `T1/Merriwthr-OsF/m/up' undefined
(Font)              using `T1/Merriwthr-OsF/m/n' instead on input line
67.

LaTeX Font Info:    Font shape `T1/Merriwthr-OsF/m/up' will be
(Font)              scaled to size 6.24973pt on input line 67.
LaTeX Font Info:    Font shape `T1/Merriwthr-OsF/m/up' will be
(Font)              scaled to size 5.24997pt on input line 67.
LaTeX Font Info:    Trying to load font information for U+eur on input
line 67.

(c:/texlive/2023/texmf-dist/tex/latex/amsfonts/ueur.fd
File: ueur.fd 2013/01/14 v3.01 Euler Roman
) (c:/texlive/2023/texmf-dist/tex/latex/microtype/mt-eur.cfg
File: mt-eur.cfg 2006/07/31 v1.1 microtype config. file: AMS Euler Roman
(RS)
)

LaTeX Font Warning: Font shape `OMS/cmsy/m/n' in size <7.5> not available
(Font)              size <7> substituted on input line 67.

LaTeX Font Info:    Trying to load font information for U+euf on input
line 67.

(c:/texlive/2023/texmf-dist/tex/latex/amsfonts/ueuf.fd
File: ueuf.fd 2013/01/14 v3.01 Euler Fraktur
) (c:/texlive/2023/texmf-dist/tex/latex/microtype/mt-euf.cfg
File: mt-euf.cfg 2006/07/03 v1.1 microtype config. file: AMS Euler
Fraktur (RS)

)
LaTeX Font Info:    Trying to load font information for U+eus on input
line 67.

(c:/texlive/2023/texmf-dist/tex/latex/amsfonts/ueus.fd
File: ueus.fd 2013/01/14 v3.01 Euler Script
) (c:/texlive/2023/texmf-dist/tex/latex/microtype/mt-eus.cfg
```

```
File: mt-eus.cfg 2006/07/28 v1.2 microtype config. file: AMS Euler Script
(RS)
)
LaTeX Font Info:    Trying to load font information for U+euex on input
line 67
.
(c:/texlive/2023/texmf-dist/tex/latex/amsfonts/ueuex.fd
File: ueuex.fd 2013/01/14 v3.01 Euler extra symbols
)

LaTeX Font Warning: Font shape `OML/cmm/m/it' in size <7.5> not available
(Font)              size <7> substituted on input line 67.

LaTeX Font Info:    Font shape `T1/Merriwthr-OsF/m/n' will be
(Font)              scaled to size 6.24973pt on input line 67.
LaTeX Font Info:    Font shape `T1/Merriwthr-OsF/m/n' will be
(Font)              scaled to size 5.24997pt on input line 67.
LaTeX Font Info:    Font shape `T1/Merriwthr-OsF/m/it' will be
(Font)              scaled to size 7.5pt on input line 67.
LaTeX Font Info:    Font shape `T1/Merriwthr-OsF/m/it' will be
(Font)              scaled to size 6.24973pt on input line 67.
LaTeX Font Info:    Font shape `T1/Merriwthr-OsF/m/it' will be
(Font)              scaled to size 5.24997pt on input line 67.
LaTeX Font Info:    Trying to load font information for U+msa on input
line 67.

(c:/texlive/2023/texmf-dist/tex/latex/amsfonts/umsa.fd
File: umsa.fd 2013/01/14 v3.01 AMS symbols A
) (c:/texlive/2023/texmf-dist/tex/latex/microtype/mt-msa.cfg
File: mt-msa.cfg 2006/02/04 v1.1 microtype config. file: AMS symbols (a)
(RS)
)
LaTeX Font Info:    Trying to load font information for U+msb on input
line 67.

(c:/texlive/2023/texmf-dist/tex/latex/amsfonts/umsb.fd
File: umsb.fd 2013/01/14 v3.01 AMS symbols B
) (c:/texlive/2023/texmf-dist/tex/latex/microtype/mt-msb.cfg
File: mt-msb.cfg 2005/06/01 v1.0 microtype config. file: AMS symbols (b)
(RS)
)
LaTeX Font Info:    Font shape `T1/Merriwthr-OsF/m/n' will be
(Font)              scaled to size 8.0pt on input line 67.
LaTeX Font Info:    Font shape `T1/Merriwthr-OsF/m/it' will be
(Font)              scaled to size 8.0pt on input line 67.
LaTeX Font Info:    Font shape `T1/Merriwthr-OsF/b/it' will be
(Font)              scaled to size 8.0pt on input line 67.
TextBlockOrigin set to 4pc+6.64pt x 4pc+6pt
<example-grid-100x100pt.png, id=152, 125.46875pt x 125.46875pt>
File: example-grid-100x100pt.png Graphic file (type png)
<use example-grid-100x100pt.png>
Package pdftex.def Info: example-grid-100x100pt.png  used on input line
82.
(pdftex.def)             Requested size: 42.00021pt x 42.0pt.
```

```
Overfull \hbox (54.64pt too wide) in paragraph at lines 82--82
[][]
 []

LaTeX Font Info:    Font shape `T1/Merriwthr-OsF/m/n' will be
(Font)              scaled to size 14.0pt on input line 82.
LaTeX Font Info:    Font shape `T1/Merriwthr-OsF/m/n' will be
(Font)              scaled to size 8.99997pt on input line 82.
LaTeX Font Info:    Calculating math sizes for size <14> on input line
82.
LaTeX Font Info:    Font shape `T1/Merriwthr-OsF/m/up' will be
(Font)              scaled to size 14.0pt on input line 82.
LaTeX Font Info:    Font shape `T1/Merriwthr-OsF/m/up' will be
(Font)              scaled to size 11.66617pt on input line 82.
LaTeX Font Info:    Font shape `T1/Merriwthr-OsF/m/up' will be
(Font)              scaled to size 9.79996pt on input line 82.
LaTeX Font Info:    Font shape `T1/Merriwthr-OsF/m/n' will be
(Font)              scaled to size 11.66617pt on input line 82.
LaTeX Font Info:    Font shape `T1/Merriwthr-OsF/m/n' will be
(Font)              scaled to size 9.79996pt on input line 82.
LaTeX Font Info:    Font shape `T1/Merriwthr-OsF/m/it' will be
(Font)              scaled to size 14.0pt on input line 82.
LaTeX Font Info:    Font shape `T1/Merriwthr-OsF/m/it' will be
(Font)              scaled to size 11.66617pt on input line 82.
LaTeX Font Info:    Font shape `T1/Merriwthr-OsF/m/it' will be
(Font)              scaled to size 9.79996pt on input line 82.
LaTeX Font Info:    Font shape `T1/Merriwthr-OsF/b/n' will be
(Font)              scaled to size 18.0pt on input line 82.
LaTeX Font Info:    Font shape `T1/Merriwthr-OsF/m/n' will be
(Font)              scaled to size 13.0pt on input line 82.
LaTeX Font Info:    Calculating math sizes for size <13> on input line
82.
LaTeX Font Info:    Font shape `T1/Merriwthr-OsF/m/up' will be
(Font)              scaled to size 13.0pt on input line 82.
LaTeX Font Info:    Font shape `T1/Merriwthr-OsF/m/up' will be
(Font)              scaled to size 10.83287pt on input line 82.
LaTeX Font Info:    Font shape `T1/Merriwthr-OsF/m/up' will be
(Font)              scaled to size 9.09996pt on input line 82.

LaTeX Font Warning: Font shape `OMS/cmsy/m/n' in size <13> not available
(Font)              size <12> substituted on input line 82.


LaTeX Font Warning: Font shape `OML/cmm/m/it' in size <13> not available
(Font)              size <12> substituted on input line 82.

LaTeX Font Info:    Font shape `T1/Merriwthr-OsF/m/n' will be
(Font)              scaled to size 10.83287pt on input line 82.
LaTeX Font Info:    Font shape `T1/Merriwthr-OsF/m/n' will be
(Font)              scaled to size 9.09996pt on input line 82.
LaTeX Font Info:    Font shape `T1/Merriwthr-OsF/m/it' will be
(Font)              scaled to size 13.0pt on input line 82.
LaTeX Font Info:    Font shape `T1/Merriwthr-OsF/m/it' will be
```

```
(Font)              scaled to size 10.83287pt on input line 82.
LaTeX Font Info:    Font shape `T1/Merriwthr-OsF/m/it' will be
(Font)              scaled to size 9.09996pt on input line 82.
LaTeX Font Info:    Trying to load font information for TS1+Merriwthr-OsF
on in
put line 82.
(c:/texlive/2023/texmf-dist/tex/latex/merriweather/TS1Merriwthr-OsF.fd
File: TS1Merriwthr-OsF.fd 2020/08/30 (autoinst) Font definitions for
TS1/Merriw
thr-OsF.
)
LaTeX Font Info:    Font shape `TS1/Merriwthr-OsF/m/n' will be
(Font)              scaled to size 10.83287pt on input line 82.
Package microtype Info: Loading generic protrusion settings for font
family
(microtype)             `Merriwthr-OsF' (encoding: TS1).
(microtype)             For optimal results, create family-specific
settings.
(microtype)             See the microtype manual for details.
LaTeX Font Info:    Font shape `T1/Merriwthr-OsF/m/n' will be
(Font)              scaled to size 9.0pt on input line 82.
LaTeX Font Info:    Font shape `T1/Merriwthr-OsF/m/up' will be
(Font)              scaled to size 9.0pt on input line 82.
LaTeX Font Info:    Font shape `T1/Merriwthr-OsF/m/up' will be
(Font)              scaled to size 7.0pt on input line 82.
LaTeX Font Info:    Font shape `T1/Merriwthr-OsF/m/up' will be
(Font)              scaled to size 5.0pt on input line 82.
LaTeX Font Info:    Font shape `T1/Merriwthr-OsF/m/n' will be
(Font)              scaled to size 7.0pt on input line 82.
LaTeX Font Info:    Font shape `T1/Merriwthr-OsF/m/n' will be
(Font)              scaled to size 5.0pt on input line 82.
LaTeX Font Info:    Font shape `T1/Merriwthr-OsF/m/it' will be
(Font)              scaled to size 9.0pt on input line 82.
LaTeX Font Info:    Font shape `T1/Merriwthr-OsF/m/it' will be
(Font)              scaled to size 7.0pt on input line 82.
LaTeX Font Info:    Font shape `T1/Merriwthr-OsF/m/it' will be
(Font)              scaled to size 5.0pt on input line 82.
LaTeX Font Info:    Font shape `T1/Merriwthr-OsF/m/n' will be
(Font)              scaled to size 6.5pt on input line 82.
LaTeX Font Info:    Calculating math sizes for size <6.5> on input line
82.
LaTeX Font Info:    Font shape `T1/Merriwthr-OsF/m/up' will be
(Font)              scaled to size 6.5pt on input line 82.
LaTeX Font Info:    Font shape `T1/Merriwthr-OsF/m/up' will be
(Font)              scaled to size 5.41643pt on input line 82.
LaTeX Font Info:    Font shape `T1/Merriwthr-OsF/m/up' will be
(Font)              scaled to size 4.54997pt on input line 82.

LaTeX Font Warning: Font shape `OMS/cmsy/m/n' in size <6.5> not available
(Font)              size <6> substituted on input line 82.


LaTeX Font Warning: Font shape `OMS/cmsy/m/n' in size <5.41643> not
available
```

```
(Font)              size <5> substituted on input line 82.


LaTeX Font Warning: Font shape `OMS/cmsy/m/n' in size <4.54997> not
available
(Font)              size <5> substituted on input line 82.


LaTeX Font Warning: Font shape `OML/cmm/m/it' in size <6.5> not available
(Font)              size <6> substituted on input line 82.


LaTeX Font Warning: Font shape `OML/cmm/m/it' in size <5.41643> not
available
(Font)              size <5> substituted on input line 82.


LaTeX Font Warning: Font shape `OML/cmm/m/it' in size <4.54997> not
available
(Font)              size <5> substituted on input line 82.

LaTeX Font Info:    Font shape `T1/Merriwthr-OsF/m/n' will be
(Font)              scaled to size 5.41643pt on input line 82.
LaTeX Font Info:    Font shape `T1/Merriwthr-OsF/m/n' will be
(Font)              scaled to size 4.54997pt on input line 82.
LaTeX Font Info:    Font shape `T1/Merriwthr-OsF/m/it' will be
(Font)              scaled to size 6.5pt on input line 82.
LaTeX Font Info:    Font shape `T1/Merriwthr-OsF/m/it' will be
(Font)              scaled to size 5.41643pt on input line 82.
LaTeX Font Info:    Font shape `T1/Merriwthr-OsF/m/it' will be
(Font)              scaled to size 4.54997pt on input line 82.
LaTeX Font Info:    Font shape `TS1/Merriwthr-OsF/m/n' will be
(Font)              scaled to size 5.41643pt on input line 82.

Overfull \hbox (54.64pt too wide) in paragraph at lines 82--82
[][][]
 []

LaTeX Font Info:    Font shape `T1/Merriwthr-OsF/b/n' will be
(Font)              scaled to size 10.0pt on input line 82.
LaTeX Font Info:    Font shape `T1/Merriwthr-OsF/b/n' will be
(Font)              scaled to size 8.0pt on input line 82.

Overfull \hbox (54.64pt too wide) in paragraph at lines 82--82
[][][]
 []

Package mdframed Info: mdframed works in twoside mode on input line 85.
LaTeX Font Info:    Font shape `T1/Merriwthr-OsF/b/n' will be
(Font)              scaled to size 8.2pt on input line 85.
LaTeX Font Info:    Font shape `TS1/Merriwthr-OsF/m/n' will be
(Font)              scaled to size 7.5pt on input line 87.
Package mdframed Info: mdframed inside float
 mdframed uses option nobreak mdframed on input line 93.
```

```
Package mdframed Info: mdframed inside a box
 mdframed uses option nobreak mdframed on input line 93.
LaTeX Font Info:    Font shape `T1/Merriwthr-OsF/b/n' will be
(Font)              scaled to size 7.5pt on input line 96.

Package natbib Warning: Citation `openai_chatgpt' on page 1 undefined on
input
line 96.


Package natbib Warning: Citation `touvron2023llama' on page 1 undefined
on inpu
t line 96.


Package natbib Warning: Citation `ji2023survey' on page 1 undefined on
input li
ne 97.


Package natbib Warning: Citation `liu2021towards' on page 1 undefined on
input
line 97.


Package natbib Warning: Citation `kang2020improved' on page 1 undefined
on inpu
t line 97.


Underfull \vbox (badness 10000) has occurred while \output is active []


Package natbib Warning: Citation `pan2024unifying' on page 1 undefined on
input
 line 99.


Package natbib Warning: Citation `han2023medalpaca' on page 1 undefined
on inpu
t line 99.


Package natbib Warning: Citation `yunxiang2023chatdoctor' on page 1
undefined o
n input line 99.


Package natbib Warning: Citation `singhal2022large' on page 1 undefined
on inpu
t line 99.
```

Package natbib Warning: Citation `wang2023huatuo' on page 1 undefined on
input
line 99.

Package natbib Warning: Citation `xiong2023doctorglm' on page 1 undefined
on in
put line 99.

Package natbib Warning: Citation `zhang2023huatuogpt' on page 1 undefined
on in
put line 99.

Package natbib Warning: Citation `yang2023zhongjing' on page 1 undefined
on inp
ut line 99.

Package natbib Warning: Citation `tian2023chimed' on page 1 undefined on
input
line 99.

Package natbib Warning: Citation `ouyang2022training' on page 1 undefined
on in
put line 99.

Package natbib Warning: Citation `luo2023empirical' on page 1 undefined
on inpu
t line 99.

Package natbib Warning: Citation `li2017learning' on page 1 undefined on
input
line 99.

Underfull \vbox (badness 10000) has occurred while \output is active []

LaTeX Font Info: Font shape `T1/Merriwthr-OsF/m/n' will be
(Font)   scaled to size 7.8pt on input line 101.
LaTeX Font Info: Font shape `T1/Merriwthr-OsF/b/n' will be
(Font)   scaled to size 7.8pt on input line 101.
[1{c:/texlive/2023/texmf-
var/fonts/map/pdftex/updmap/pdftex.map}{c:/texlive/202
3/texmf-
dist/fonts/enc/dvips/merriweather/merriwthr_posqbl.enc}{c:/texlive/2023
/texmf-dist/fonts/enc/dvips/merriweather/merriwthr_owzwzj.enc}

<c:/texlive/2023/texmf-dist/tex/latex/mwe/example-grid-100x100pt.png>]

Package natbib Warning: Citation `liu2022design' on page 2 undefined on input l
ine 104.

Package natbib Warning: Citation `wei2022chain' on page 2 undefined on input li
ne 104.

Package natbib Warning: Citation `zhou2022large' on page 2 undefined on input l
ine 104.

Package natbib Warning: Citation `sun2018open' on page 2 undefined on input lin
e 106.

Package natbib Warning: Citation `sun2019pullnet' on page 2 undefined on input
line 106.

Package natbib Warning: Citation `zhang2022subgraph' on page 2 undefined on inp
ut line 106.

Package natbib Warning: Citation `chen2019bidirectional' on page 2 undefined on
 input line 106.

Package natbib Warning: Citation `saxena2020improving' on page 2 undefined on i
nput line 106.

Package natbib Warning: Citation `lan2021survey' on page 2 undefined on input l
ine 106.

Package natbib Warning: Citation `das2021case' on page 2 undefined on input lin
e 106.

Package natbib Warning: Citation `jiang2022unikgqa' on page 2 undefined on inpu
t line 106.

```
Package natbib Warning: Citation `jiang2023structgpt' on page 2 undefined
on in
put line 106.


Package natbib Warning: Citation `sunthink' on page 2 undefined on input
line 1
06.


Package natbib Warning: Citation `luoreasoning' on page 2 undefined on
input li
ne 106.


Package natbib Warning: Citation `kim2023kg' on page 2 undefined on input
line
106.


Underfull \vbox (badness 3260) has occurred while \output is active []



pdfTeX warning: pdflatex.exe (file ./first.pdf): PDF inclusion: found PDF
versi
on <1.7>, but at most version <1.5> allowed
<first.pdf, id=167, 352.43669pt x 415.5525pt>
File: first.pdf Graphic file (type pdf)
<use first.pdf>
Package pdftex.def Info: first.pdf  used on input line 111.
(pdftex.def)             Requested size: 235.11394pt x 277.22665pt.
LaTeX Font Info:    Font shape `T1/Merriwthr-OsF/m/n' will be
(Font)              scaled to size 6.0pt on input line 112.
LaTeX Font Info:    Font shape `T1/Merriwthr-OsF/b/n' will be
(Font)              scaled to size 6.0pt on input line 112.

Package natbib Warning: Citation `feng2024knowledge' on page 2 undefined
on inp
ut line 116.


Underfull \vbox (badness 10000) has occurred while \output is active []

LaTeX Font Info:    Font shape `T1/Merriwthr-OsF/m/it' will be
(Font)              scaled to size 7.8pt on input line 117.
[2 <./first.pdf>]
LaTeX Font Info:    Font shape `T1/Merriwthr-OsF/b/n' will be
(Font)              scaled to size 8.5pt on input line 119.
LaTeX Font Info:    Font shape `T1/Merriwthr-OsF/m/up' will be
(Font)              scaled to size 7.5pt on input line 120.
```

```
Package natbib Warning: Citation `wang2022synlethdb' on page 3 undefined
on inp
ut line 122.


Package natbib Warning: Citation `zhu2022multimodal' on page 3 undefined
on inp
ut line 122.


Package natbib Warning: Citation `feng2024knowledge' on page 3 undefined
on inp
ut line 122.

LaTeX Font Info:    Font shape `T1/Merriwthr-OsF/b/n' will be
(Font)              scaled to size 7.0pt on input line 127.

Package natbib Warning: Citation `zhang2018variational' on page 3
undefined on
input line 140.


Package natbib Warning: Citation `kim2023factkg' on page 3 undefined on
input l
ine 140.


Underfull \vbox (badness 10000) has occurred while \output is active []

LaTeX Font Info:    Font shape `T1/Merriwthr-OsF/b/sl' in size <7.5> not
availa
ble
(Font)              Font shape `T1/Merriwthr-OsF/b/it' tried instead on
input l
ine 142.
LaTeX Font Info:    Font shape `T1/Merriwthr-OsF/b/it' will be
(Font)              scaled to size 7.5pt on input line 142.

Underfull \vbox (badness 3098) has occurred while \output is active []

[3]

Package natbib Warning: Citation `achiam2023gpt' on page 4 undefined on
input l
ine 192.


Package natbib Warning: Citation `feng2024knowledge' on page 4 undefined
on inp
ut line 192.


Overfull \hbox (4.30591pt too wide) in paragraph at lines 198--252
 [][]
```

```
 []



pdfTeX warning: pdflatex.exe (file ./framework.pdf): PDF inclusion: found
PDF v
ersion <1.7>, but at most version <1.5> allowed
<framework.pdf, id=213, 1178.48276pt x 597.19106pt>
File: framework.pdf Graphic file (type pdf)
<use framework.pdf>
Package pdftex.def Info: framework.pdf  used on input line 260.
(pdftex.def)             Requested size: 488.22787pt x 247.40138pt.
LaTeX Font Info:    Font shape `T1/Merriwthr-OsF/m/up' will be
(Font)              scaled to size 6.0pt on input line 261.
LaTeX Font Info:    Font shape `T1/Merriwthr-OsF/m/it' will be
(Font)              scaled to size 6.0pt on input line 261.

Package natbib Warning: Citation `achiam2023gpt' on page 4 undefined on
input l
ine 308.


Package natbib Warning: Citation `zhang2019bertscore' on page 4 undefined
on in
put line 308.


Package natbib Warning: Citation `lin2004rouge' on page 4 undefined on
input li
ne 308.

[4]

Package natbib Warning: Citation `kim2023kg' on page 5 undefined on input
line
313.


Package natbib Warning: Citation `wei2022chain' on page 5 undefined on
input li
ne 313.


Package natbib Warning: Citation `dong2022survey' on page 5 undefined on
input
line 313.


Package natbib Warning: Citation `kim2023kg' on page 5 undefined on input
line
313.
```

```
Package natbib Warning: Citation `roziere2023code' on page 5 undefined on
input
 line 313.


Package natbib Warning: Citation `openai_chatgpt' on page 5 undefined on
input
line 315.


Package natbib Warning: Citation `luo2023taiyi' on page 5 undefined on
input li
ne 315.


Underfull \vbox (badness 10000) has occurred while \output is active []

[5 <./framework.pdf>]

Package natbib Warning: Citation `tunstall2023zephyr' on page 6 undefined
on in
put line 357.


Package natbib Warning: Citation `touvron2023llama' on page 6 undefined
on inpu
t line 357.


Package natbib Warning: Citation `roziere2023code' on page 6 undefined on
input
 line 357.

<Scalability.pdf, id=253, 459.6372pt x 348.34142pt>
File: Scalability.pdf Graphic file (type pdf)
<use Scalability.pdf>
Package pdftex.def Info: Scalability.pdf  used on input line 360.
(pdftex.def)             Requested size: 235.11394pt x 178.18329pt.
[6 <./Scalability.pdf>]

Package natbib Warning: Citation `he2022analysis' on page 7 undefined on
input
line 398.


Package natbib Warning: Citation `gan2018prospective' on page 7 undefined
on in
put line 399.



pdfTeX warning: pdflatex.exe (file ./examples.pdf): PDF inclusion: found
PDF ve
rsion <1.7>, but at most version <1.5> allowed
```

```
<examples.pdf, id=266, 438.67888pt x 501.79468pt>
File: examples.pdf Graphic file (type pdf)
<use examples.pdf>
Package pdftex.def Info: examples.pdf  used on input line 406.
(pdftex.def)             Requested size: 235.11394pt x 268.94371pt.

Package natbib Warning: Citation `gottesman2002mechanisms' on page 7
undefined
on input line 413.


Package natbib Warning: Citation `alshareef2016use' on page 7 undefined
on inpu
t line 414.


Package natbib Warning: Citation `simionato2015current' on page 7
undefined on
input line 414.


Underfull \vbox (badness 10000) has occurred while \output is active []

[7 <./examples.pdf>]
Underfull \hbox (badness 3396) in paragraph at lines 440--441
[]\T1/Merriwthr-OsF/m/up/7.5 (+20) Project home-page:
[]https://github.com/yich
un10/bioKGQA-
 []


Package natbib Warning: Citation `feng2024knowledge' on page 8 undefined
on inp
ut line 452.


Package natbib Warning: Citation `bioKGQA2024github' on page 8 undefined
on inp
ut line 452.


Package natbib Warning: Citation `openai_chatgpt' on page 8 undefined on
input
line 452.


Package natbib Warning: Citation `touvron2023llama' on page 8 undefined
on inpu
t line 452.


Package natbib Warning: Citation `achiam2023gpt' on page 8 undefined on
input l
ine 452.
```

```
Package natbib Warning: Citation `roziere2023code' on page 8 undefined on
input
 line 452.


Package natbib Warning: Citation `luo2023taiyi' on page 8 undefined on
input li
ne 452.


Package natbib Warning: Citation `tunstall2023zephyr' on page 8 undefined
on in
put line 452.

LaTeX Font Info:    Font shape `T1/Merriwthr-OsF/m/n' will be
(Font)              scaled to size 10.0pt on input line 474.
[8]
No file manuscript_file.bbl.

Package natbib Warning: There were undefined citations.

[9

]
enddocument/afterlastpage: lastpage setting LastPage.
(./manuscript_file.aux)
 ***********
LaTeX2e <2023-11-01> patch level 1
L3 programming layer <2020/03/25>
 ***********

LaTeX Font Warning: Size substitutions with differences
(Font)              up to 1.0pt have occurred.


LaTeX Font Warning: Some font shapes were not available, defaults
substituted.

Package rerunfilecheck Info: File `manuscript_file.out' has not changed.
(rerunfilecheck)             Checksum:
DC875499719793A87CE0F8A3D5054831;6166.
 )
Here is how much of TeX's memory you used:
 36599 strings out of 474121
 729607 string characters out of 5747949
 1981190 words of memory out of 5000000
 57618 multiletter control sequences out of 15000+600000
 1888318 words of font info for 608 fonts, out of 8000000 for 9000
 1141 hyphenation exceptions out of 8191
 123i,18n,131p,2124b,1217s stack positions out of
10000i,1000n,20000p,200000b,200000s
```

```
<c:/texlive/2023/texmf-dist/fonts/type1/sorkin/merriweather/Merriwthr-
Bold.pf
b><c:/texlive/2023/texmf-dist/fonts/type1/sorkin/merriweather/Merriwthr-
BoldIta
lic.pfb><c:/texlive/2023/texmf-
dist/fonts/type1/sorkin/merriweather/Merriwthr-I
talic.pfb><c:/texlive/2023/texmf-
dist/fonts/type1/sorkin/merriweather/Merriwthr
-Regular.pfb><c:/texlive/2023/texmf-
dist/fonts/type1/public/amsfonts/cmextra/cm
ex8.pfb><c:/texlive/2023/texmf-
dist/fonts/type1/public/amsfonts/cm/cmsy6.pfb><c
:/texlive/2023/texmf-
dist/fonts/type1/public/amsfonts/cm/cmsy7.pfb><c:/texlive/
2023/texmf-
dist/fonts/type1/public/amsfonts/euler/euex8.pfb><c:/texlive/2023/te
xmf-dist/fonts/type1/public/amsfonts/symbols/msam7.pfb>
Output written on manuscript_file.pdf (9 pages, 736393 bytes).
PDF statistics:
 355 PDF objects out of 1000 (max. 8388607)
 300 compressed objects within 3 object streams
 68 named destinations out of 1000 (max. 500000)
 213318 words of extra memory for PDF output out of 221844 (max.
10000000)
```

Manuscript

Click here to access/download;Manuscript;Revision_Knowledge Graph-

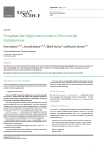

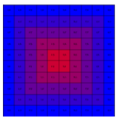

Journal of X, 2023, 1–10

Manuscript in Preparation
Research

RESEARCH

Knowledge Graph-based Thought: a knowledge graph enhanced LLMs framework for pan-cancer question answering

Yichun Feng[1,2,†], Lu Zhou[2,†], Chao Ma[3,†], Yikai Zheng[2], Ruikun He[4,5,*] and Yixue Li[1,2,*]

[1]Hangzhou Institute for Advanced Study, University of Chinese Academy of Sciences, 310024 Hangzhou, China and [2]Guangzhou National Laboratory, No. 9 XingDaoHuanBei Road, Guangzhou International Bio Island, 510005 Guangzhou, China and [3]Smartquerier Gene Technology (Shanghai) Co., Ltd., 200100 Shanghai, China and [4]BYHEALTH Institute of Nutrition & Health, 510663 Guangzhou, China and [5]Shanghai Institute of Nutrition and Health, Chinese Academy of Sciences Shanghai, 200030 Shanghai, China

[*]Correspondence address. Yixue Li, Guangzhou National Laboratory, No. 9 XingDaoHuanBei Road, Guangzhou International Bio Island, 510005 Guangzhou, China. E-mail: yxli@sibs.ac.cn; Ruikun He, BYHEALTH Institute of Nutrition & Health, 510663 Guangzhou, China. E-mail: herk@by-health.com
[†]Contributed equally.

Abstract

Background. In recent years, Large Language Models (LLMs) have shown promise in various domains, notably in biomedical sciences. However, their real-world application is often limited by issues like erroneous outputs and hallucinatory responses.
Results. We developed the Knowledge Graph-based Thought (KGT) framework, an innovative solution that integrates LLMs with Knowledge Graphs (KGs) to improve their initial responses by utilizing verifiable information from KGs, thus significantly reducing factual errors in reasoning. The KGT framework demonstrates strong adaptability and performs well across various open-source LLMs. Notably, KGT can facilitate the discovery of new uses for existing drugs through potential drug-cancer associations, and can assist in predicting resistance by analyzing relevant biomarkers and genetic mechanisms. To evaluate the Knowledge Graph Question Answering (KGQA) task within biomedicine, we utilize a pan-cancer knowledge graph to develop a pan-cancer question answering benchmark, named the Pan-cancer Question Answering (PcQA).
Conclusions. The KGT framework substantially improves the accuracy and utility of LLMs in the biomedical field. This study serves as a proof-of-concept, demonstrating its exceptional performance in biomedical question answering.

Key words: pan-cancer knowledge graph, large language model, knowledge graph question answering, prompt engineering

Introduction

With the increasing prominence of Large Language Models (LLMs) in the field of artificial intelligence, the advent of influential models such as ChatGPT [1] and Llama [2] consequently catalyze the development of a wide array of applications in biomedicine and healthcare. However, LLMs still face the challenge of factual hallucination, where they generate incorrect statements due to limited inherent knowledge [3]. Factual hallucination presents a significant challenge for the practical use of LLMs, especially in real-world scenarios where factual accuracy is crucial. Consequently, there is a growing focus on addressing factual hallucinations in LLMs within the field of Natural Language Processing (NLP) [4, 5].

LLMs often struggle to capture and access factual knowledge, primarily due to three aspects: the inability to comprehend questions due to the lack of contextual information, the insufficient

Compiled on: October 1, 2024.
Draft manuscript prepared by the author.

Key Points

- We introduce a framework combining LLMs with KGs to improve factual accuracy in LLM reasoning.
- Our system is a flexible architecture that seamlessly integrates various LLMs.
- Utilizing a pan-cancer knowledge graph, we have proposed the first KGQA benchmark in the field of biomedicine.
- Case studies reveal our method enhanced LLMs in addressing biomedical challenges such as drug repositioning, resistance research, individualized treatment, and biomarker analysis.
- The method performs favorably in comparison to existing methods.

knowledge to generate accurate answers, and the incapacity to recall specific facts [6]. Consequently, researchers consider the fine-tuning technique as a solution to address these issues. For example, MedAlpaca [7] builds upon medical data to fine-tune Stanford Alpaca for applications related to medical question-answering and dialogue. ChatDoctor [8] is designed to simulate a conversation between a doctor and a patient by fine-tuning LLaMA with medical literature. Additionally, Med-PaLM [9] shows promising performance on the MedQA exam based on clinical corpora and human feedback. Meanwhile, aiming at the Chinese medical domain, LLMs such as BenTsao [10], DoctorGLM [11], and HuatuoGPT [12], are developed on the Chinese medical dialogue data. More recently, Zhongjing [13] and ChiMed-GPT [14] adopted full pipeline training from pre-training, SFT, to Reinforcement Learning with Human Feedback (RLHF) [15]. While fine-tuning can reduce hallucinations in large language models (LLMs), it brings about considerable training expenses. Additionally, it poses a critical challenge known as catastrophic forgetting. This issue manifests when a model forgets its previously learned information as a consequence of parameter modifications during the acquisition of new tasks. This forgetfulness results in a deterioration of performance on prior tasks, consequently constraining the model's practical applicability [16, 17].

In addition to fine-tuning, researchers also enhance the output of LLMs through the field of prompt engineering. Prompt engineering focuses on the creation and optimization of prompts to improve the effectiveness of LLMs across various applications and research domains [18]. It can enhance the capabilities of LLMs in a wide range of complex tasks, including question answering, sentiment classification, and common-sense reasoning. Chain-of-thought (CoT) prompts [19] enable complex reasoning capabilities by incorporating intermediate reasoning steps. The Automatic Prompt Engineer (APE) proposes an automatic prompt generation method aimed at enhancing the performance of LLMs [20]. Prompt engineering offers a straightforward approach to harnessing the potential of LLMs without fine-tuning.

On the other hand, Knowledge Graphs (KGs) are repositories of vast quantities of high-quality structured data, offering the potential to effectively mitigate the issue of factual hallucinations when integrated with LLMs. Hence, employing KGs for question-answering can enhance the precision of the responses and furnish a dependable foundation for the factual verification of information produced by LLMs. Knowledge Graph Question Answering (KGQA) has long been a hot research topic. Before the advent of LLMs, certain studies [21, 22, 23] typically begin by retrieving a subgraph related to the question to reduce the search space, then perform multi-hop reasoning on this basis. This retrieval-plus-reasoning paradigm has shown its advantages over direct reasoning across the entire KG [24, 25]. Additionally, Researchers tackle KGQA by parsing the question into a structured query language (e.g., SPARQL) and using a query engine to obtain accurate answers [26, 27]. UniKGQA [28] introduces a unified fine-tuning framework for retrieval and reasoning, more closely linking these two stages. However, traditional KGQA methods usually perform poorly in accurate semantic understanding and high-quality text generation due to the lack of LLMs for retrieval and reasoning. Hence, recent research is in-

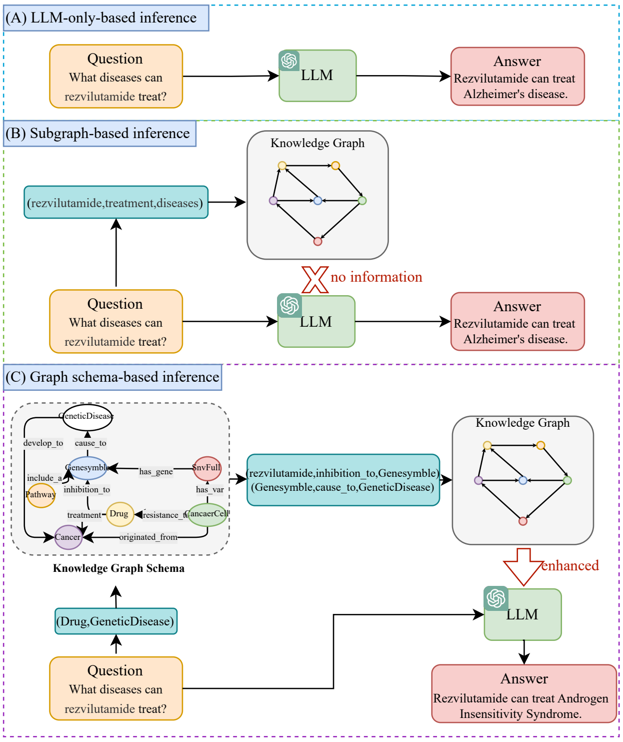

Figure 1. Illustrative examples contrasting our work with previous efforts. **(A) LLM-only-based inference**, answering questions solely through the inherent knowledge of LLMs. **(B) Subgraph-based inference**, enhancing LLMs by retrieving the knowledge from KGs based on the question. If intermediate entities are not provided in the multi-hop question, no appropriate knowledge can be retrieved. **(C) Graph schema-based inference**, enhancing retrieval capabilities by reasoning intermediary entity types on the schema of the KG, using the knowledge of the KG to enhance LLMs' responses.

creasingly utilizing external KGs to enhance LLMs in addressing KGQA challenges. For instance, StructGPT [29] navigates through knowledge graphs by identifying pathways from an initial seed entity to the target answer entity, while Think-on-Graph (ToG) [30] introduces iterative exploration of the knowledge graph, which can become inefficient with very large KGs. Additionally, RoG [31] necessitates fine-tuning to accurately generate and plan the relation paths. KG-GPT [32] opts for retrieving an entire subgraph from the knowledge graph and then deduces the answer through inference. Although these methods have achieved gratifying results in general areas, as shown in Figure 1(B), when the intermediate entity in the multi-hop question is unknown, it is impossible to retrieve the appropriate knowledge from the KG.

In this paper, we introduce an innovative framework called Knowledge Graph-based Thought (KGT), which integrates LLMs with KGs through employing LLMs for reasoning on the schema of KGs to mitigate factual hallucinations of LLMs, as shown in Fig. 1(C). Unlike traditional methods, KGT does not directly retrieve factual information based on the question. Instead, it uses LLMs to infer en-

Table 1. Comparison of SOKG with SynLethKG and SDKG

	Entity Types	Relational Types	Nodes	Edges	Attributes
SynLethKG	11	24	54,012	2,231,921	0
SDKG	7	12	165,062	727,318	0
**SOKG**	**24**	**21**	**3,640,259**	**10,656,273**	**98**

tity information on the schema of the knowledge graph, generating an optimal subgraph based on key information directly extracted from the question and inferred information from the schema. Subsequently, the optimal subgraph is used to infer the answer to the question through LLMs. KGT requires no fine-tuning, offers seamless integration with multiple LLMs, and is plug-and-play, facilitating easy deployment. It demonstrates generalizability, making it adaptable for use with diverse knowledge graphs. This framework is tailored for wide-ranging applications in numerous biomedical challenges, such as: (1) enhancing clinical decision-making for physicians and medical organizations; (2) delivering medical advice to patients and healthcare providers; (3) uncovering crucial biomarkers for early disease detection and tailored therapy; and (4) exploring novel therapeutic applications for existing medications through insights into their mechanisms, side effects, and the biological processes of associated diseases. Furthermore, we utilize the SmartQuerier Oncology Knowledge Graph (SOKG), a pan-cancer knowledge graph developed by SmartQuerier, to create a benchmark for the Knowledge Graph Question Answering task within biomedicine, named the Pan-cancer Question Answering (PcQA). We release this benchmark and its accompanying knowledge graph, which is a subgraph of the SOKG, in [33]. This benchmark is currently the sole question answering dataset available in the domain of biomedical knowledge graphs.

Materials and Methods

Knowledge graph introduction

In this work, we tackle the problem of logical reasoning over the KG $\mathcal{K} : E \times R$ that store entities (E) and relations (R). Without loss of generality, KG can be organized as a set of triplets $\{(e_1, r, e_2)\} \subseteq \mathcal{K}$, where each relation $r \in R$ exists between the pair of entities $(e_1, e_2) \in E \times E$. We define a relational path $\{(t_1, r, t_2)\}$ as a sequence of entity types (T) and the relation between them, where $(t_1, t_2) \in T \times T$. In contrast, a relational chain $\{(e_1, r, e_2)\}$ refers to a specific set of relational triplets between entities. To further enrich the KG, attribute information is included through pairs $(e, attr)$, where $attr$ represents an attribute associated with an entity e, thereby enhancing the KG's semantic richness and precision by incorporating detailed characteristics of each entity.

Within the specialized realm of pan-cancer research, we use a subgraph of the SOKG that provides detailed oncological information. As depicted in Table 1, SOKG includes a collection of over 3 million entities, which is substantially larger than the entity count in the compared knowledge graphs, SynLethKG [34] and SDKG [35], with 540,012 and 165,062 entities, respectively. Furthermore, SOKG's nearly 6 million unique concept relations exceed those of SynLethKG and SDKG, which have 2,231,921 and 727,318 relations, respectively. Additionally, SOKG includes 98 distinct attribute types, enriching data comprehension and improving the efficiency and precision of queries, a capability not matched by SynLethKG or SDKG, which do not include comparable attributes. For this research, we utilize only a subgraph of the SOKG, which is available as open data [33], while the full knowledge graph remains proprietary.

Tasks description

In order to tackle a diverse array of challenges in the field of biomedicine, we have designed four categories of problems: one-hop problems, multi-hop problems, intersection problems, and attribute problems, as illustrated in Table 2. Based on these four types of tasks, we leverage the SOKG to establish a benchmark for the Knowledge Graph Question Answering task within biomedicine, named the Pan-cancer Question Answering (PcQA). Unlike KGQA tasks in general domains, such as MetaQA[36] and FACTKG[37], which typically provide the entity types of intermediate entities, KGQA problems in the biomedical domain often do not have any information about intermediate entities. Instead, the information about intermediate entities must be inferred from the question itself rather than being directly provided as shown in Supplementary Material Table.S1. Additionally, our PcQA dataset includes attributes such as whether a drug is targeted therapy or if a mutated gene is oncogenic. This makes our tasks slightly more challenging and better suited to the actual needs of biomedical KGQA.

One-hop problems

One-hop problems involve single-relation chain reasoning, where the objective is to deduce the tail entity $T_?$ given a head entity H_1 and a relation R_1, or to infer the relation $R_?$ when a head entity H_1 and a tail entity T_1 are known, as depicted in Equ.1 and Equ.2.

$$H_1 + R_1 \rightarrow T_? \tag{1}$$

$$H_1 + T_1 \rightarrow R_? \tag{2}$$

Multi-hop problems

Multi-hop problems involve multiple-relation chain reasoning, that can be broadly categorized into two types. The first category involves deducing potential relationships between entities by navigating through indirect relations. By examining the indirect relations (R_1,R_2) between a head entity H_1 and a tail entity T_1, it is possible to infer an unknown or potential relation $R_?$ linking them directly. This inference process is encapsulated in the following equation:

$$H_1 + T_1 \rightarrow R_1 + R_2 \rightarrow R_? \tag{3}$$

The second category extends the reasoning to include the discovery of entities themselves, by following a path from a head entity through intermediate relations to a final tail entity. Starting with a head entity H_1, coupled with an indirect relation R_1, an intermediary entity M can be inferred. This intermediary entity M is then applied with an indirect relation R_2 to deduce the final tail entity $T_?$. This inference process is summarized in the following equation:

$$H_1 + R_1 \rightarrow M + R_2 \rightarrow T_? \tag{4}$$

Intersection problems

Intersection problems refer to taking the intersection of multiple relational chains. Two head entities (H_1,H_2) lead to the deduction of two types of tail entities (T_1,T_2) based on different relations (R_1,R_2). The final tail entity $T_?$ is determined by intersecting these two types of tail entities (T_1,T_2). This inference process is summarized

as following:

$$H_1 + R_1 \rightarrow T_1 \tag{5}$$

$$H_2 + R_2 \rightarrow T_2 \tag{6}$$

$$T_1 \cap T_2 \rightarrow T_? \tag{7}$$

Attribute problems

Attribute problems refer to the attribute information of entity, where the task involves retrieving the attributes of a known head entity H_1 or determining whether the tail entity T_1, identified through a known head entity H_1 and relation R_1, satisfies the attributes specified in the query, as illustrated in Equ.8 and Equ.9.

$$H_1 \rightarrow P_{H_1} \tag{8}$$

$$H_1 + R_1 \rightarrow P_{T_1} \tag{9}$$

Datasets

In the continuously evolving field of biomedical research, the integration of LLMs with KGs offers a more efficient and effective method for knowledge discovery and utilization, particularly in advancing cancer research. Nonetheless, we note a scarcity of appropriate datasets for evaluating these sophisticated methodologies within this field. To address this, we leverage the SOKG to establish a benchmark for the Knowledge Graph Question Answering task within biomedicine, named the Pan-cancer Question Answering (PcQA). Our questions were carefully crafted by experts based on the content of the knowledge graph. GPT-4 [38] was then employed to generate Cypher queries, which were used to retrieve answers from the knowledge graph. The generated Cypher queries and corresponding answers underwent an initial review by a biomedical PhD candidate, who manually verified and corrected the dataset against the knowledge graph. Finally, the entire dataset was thoroughly reviewed by two biomedical experts to ensure its accuracy and reliability. This multi-step process was meticulously designed to uphold the highest standards of quality throughout the dataset creation. This dataset, along with the accompanying knowledge graph, is completely open-source[33]. The PcQA includes 405 data entries, covering a wide range of applications in the field of pan-cancer research, including genetic predisposition to cancer, medication treatment planning, drug repositioning, identification of potential drug targets, studies on drug resistance, and predictions of cancer progression and metastasis. By deeply exploring cancer-related reasoning and information retrieval challenges, this dataset can inspire researchers and clinicians to gain a deeper understanding of cancer and explore more effective treatment methods.

KGT framework

The overall framework of KGT is laid out in Fig. 2. When users input their question in natural language, the first step is to analyze the question, extracting the main information with the goal of breaking down the question into smaller, more manageable units. This main information is then passed to a LLM, which applies graph reasoning on the schema graph of the knowledge graph, yielding the optimal relational path. Subsequently, a retrieval statement is generated, and a subgraph is constructed within the KG through search. The relational chains and attributes in the subgraph are then fed back into the LLM to finalize the reasoning and generate an output in natural language.

Question analysis

Key information extraction. The user inputs a question text (Q) in natural language, which is initially deconstructed and parsed. A LLM is applied to analyze the question, resulting in the identification of the head entity name (H_n), the tail entity type (T_t), and the attributes of tail entity (T_a). The prompt for the LLM to extract key information from the question is presented in Supplementary Material Fig.S1.

Retrieving key information from KG. Based on H_n, a fixed Cypher format is set to query the head entity type (H_t), facilitating subsequent reasoning.

Graph Schema-based inference

Construction of a graph based on KG schema. Based on the entity types (E_t) and the relations (R) between them in the SOKG, an undirected graph $\mathcal{G}$ is established where E_t serve as nodes $\mathcal{N}$ and R act as edges $\mathcal{P}$.

Candidate Path Search. Breadth-First Search (BFS) is employed to identify the shortest paths connecting H_t and T_t from the constructed graph $\mathcal{G}$. Initiate the search at H_t, creating a queue to hold nodes encountered along the way. Simultaneously, form a set to track nodes that have been visited to avoid revisiting them. Insert H_t into the queue. Continue processing as long as the queue remains non-empty, removing a node from the queue at each step. For each of its unvisited neighbors, enqueue the neighbor, mark it as visited, and log the pathway from H_t to this neighbor. Upon arrival at T_t, use the accumulated path data to compile the set of shortest paths (SPs) from H_t to T_t, with each individual path within the set referred to as an SP. The nodes in each SP represent entity types, while the edges denote the relationships between these entity types.

Optimal path selection. By utilizing embedding technology, textual information is mapped into a low-dimensional space, resulting in N-dimensional real-value vectors. The similarity between each SP and the Q is calculated based on their respective real-value vectors, with the SP exhibiting the highest similarity being selected as the optimal path (OP).

$$\begin{aligned}\text{Similarity}(Q, SP) &= \frac{Q \cdot SP}{\|Q\| \times \|SP\|} \\ &= \frac{\sum_{i=1}^{n}(Q_i \times SP_i)}{\sqrt{\sum_{i=1}^{n} Q_i^2} \times \sqrt{\sum_{i=1}^{n} SP_i^2}}\end{aligned} \tag{10}$$

$$\text{OP} = \max_{Q,SP} \text{Similarity}(Q, SP) \tag{11}$$

Subgraph construction

Query statement generation. Input H_t, H_n, T_t, T_a, and OP into an LLM to generate a query statement, such as Cypher. Text2Cypher Prompt is presented in Supplementary Material Fig.S2.

Subgraph generation. Enter the query statement in the KG to obtain a reasonable subgraph.

Inference

Subgraph inference. Based on the relational chains and attribute data in the subgraph, determine the relevance to the question text. Prune any erroneous information, retaining only the correct relational chains.

Natural language output. The LLM divides the subgraph into multiple relational chains, each of which outputs a sentence in natural language, and then the LLM generates natural language output. LLMs Inference and Output Prompt is presented in Supplementary Material Fig.S3.

Results

Table 2. Four different reasoning types of task. Each reasoning type may include overlapping questions, so the sum across the four different reasoning types of the task may exceed the total number of questions.

Reasoning Type	Claim Example	Graph	Question Number
One-hop	What types of cancer can be treated with diethylstilbestrol?	$H1 \xrightarrow{R_1} T1$	243
Multi-hop	What genetic mutations are present in adenoid cystic carcinoma?	$H1 \xrightarrow{R_1} M \xrightarrow{R_2} T1$	124
Intersection	Which drugs are ALK in basaloid large cell carcinoma of the lung sensitivity to?	$H_1 \xrightarrow{R_1} T_1$, $H_2 \xrightarrow{R_2} T_2$; $T_1, T_2 \rightarrow \cap \rightarrow T_?$	37
Attribute	What is the maximum age for recruitment of clinical trials for patients with meningioma?	$H1 \xrightarrow{R_1} P_{T1}$	59

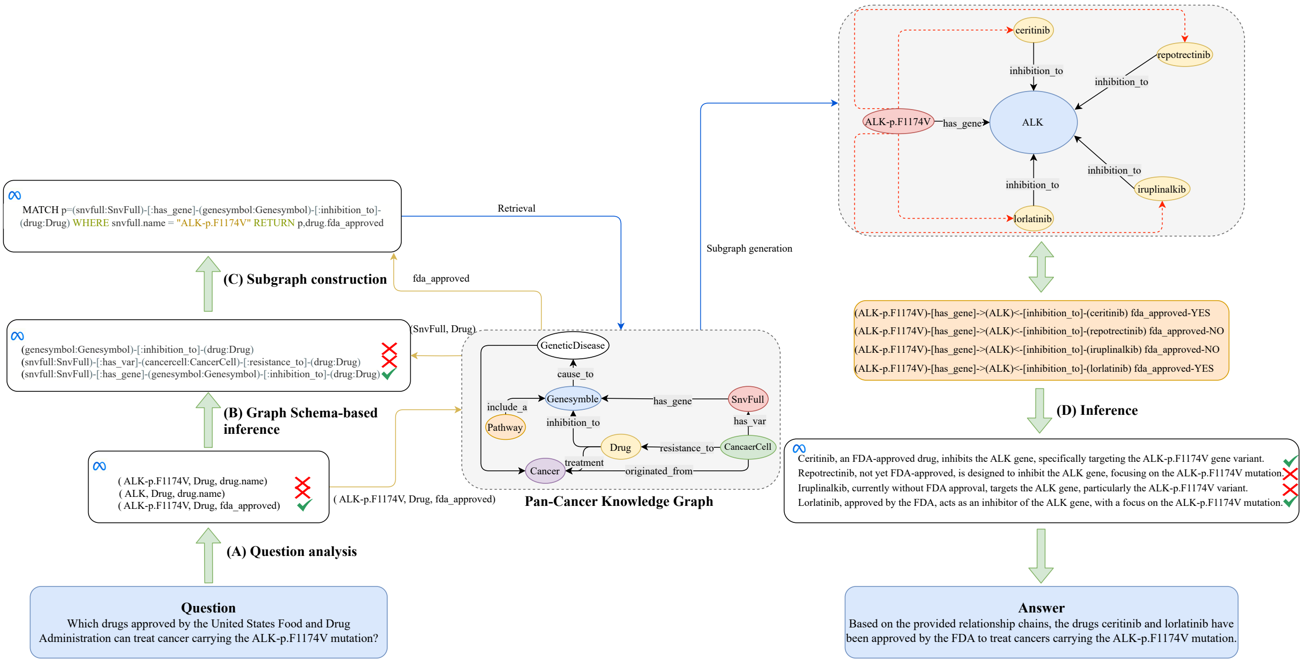

Figure 2. Framework of KGT. (A) Question analysis. Decompose the question and extract its key information. (B) Graph Schema-based inference. Input the types of the head and tail entities into the graph schema of the knowledge graph, complete the graph reasoning, and obtain the optimal relational path. (C) Subgraph construction. Generate a query statement and retrieve the subgraph. (D) Inference. Complete the final reasoning and output the results in natural language. Note: The symbol "×" represents content that has been filtered out by the LLM, while "✓" denotes the optimal content selected by the LLM.

Evaluation criteria

We use evaluators based on GPT-4 [38], BERTScore [39], and ROUGE [40] to assess the accuracy of the generated answers. As a scoring bot, GPT-4 evaluates and assigns scores based on the similarity in meaning between two sentences. GPT-4-based Evaluation Prompt is presented in Supplementary Material Fig.S4. BERTScore evaluates semantic similarity using context-sensitive embeddings, offering a comprehensive evaluation of language model outputs. ROUGE, on the other hand, evaluates the longest common subsequence (LCS) between the generated text and the reference text, focusing on sequence-based similarity to assess the fluency and the preservation of semantic content.

Baselines

To assess the advantages of our framework, we compare it with several approaches that can be directly applied for KGQA tasks without fine-tuning. We introduce a straightforward baseline approach, named Base, which is similar to KG-GPT [32], currently the leading method in the KGQA field, excluding the sentence segmentation step of KG-GPT. Initially, this involves leveraging a LLM to retrieve relevant information from the KG by generating a query statement. Then, another LLM is used to answer the question with the retrieved information. To enhance the baseline, we incorporate Chain-of-Thought (CoT) prompting [19] and In-Context Learning (ICL) techniques [41] , collectively referred to as CoT&ICL. The prompts for these methods are illustrated in Supplementary Material Table.S5. Additionally, we implement KG-GPT [32] to enhance the retrieval and reasoning capabilities of the LLMs. For a fair comparison, all methods are based on Code-Llama-13B [42].

To further underscore the efficacy of our framework, we conduct a comparative analysis of KGT, which is built upon Code-Llama-13B, against two highly capable large language models that are prominent in the general and biomedical domains: ChatGPT-3.5 [1] and Taiyi [43]. ChatGPT-3.5, a leader in tasks across the general domain, has exhibited competitive performance in a wide range of applications. To compensate for its limited biomedical knowledge, we employed two methodologies previously described, Base and CoT&ICL, as advanced baselines to augment ChatGPT-3.5's capabilities. Taiyi, a cutting-edge LLM in biomedicine, pre-trained on two trillion tokens, leverages its extensive biomedical knowledge base

Table 3. Comparison of results between KGT and other commonly used methods based on the Code-Llama-13B. Display the best results in bold for each indicator.

Method	GPT-4 Eval (%)	BERTScore (%)	ROUGE (%)		
			Recall	Precision	F1-score
Base	46.6	85.3	25.3	28.5	24.5
CoT&ICL	57.9	88.8	38.9	39.4	37.6
KG-GPT	68.2	93.5	55.2	55.8	53.3
**KGT(ours)**	**92.4**	**97.7**	**87.4**	**87.7**	**86.8**

Table 4. Comparison of KGT based on Code-Llama-13B with results from other commonly used models. Display the best results in bold for each indicator.

Model	Method	GPT-4 Eval (%)	BERTScore (%)	ROUGE (%)		
				Recall	Precision	F1-score
ChatGPT-3.5	Base	65.4	91.0	42.7	32.3	34.1
	CoT&ICL	70.3	93.3	57.0	50.6	50.5
taiyi	\	40.6	85.3	15.4	39.6	19.5
**Code-Llama-13B**	**KGT(ours)**	**92.4**	**97.7**	**87.4**	**87.7**	**86.8**

for direct question answering, bypassing the need for knowledge graph retrieval.

Due to the scarcity of KGQA datasets within the biomedical domain, all experiments are conducted on our newly proposed benchmark, named PcQA.

Comparative analysis across different KGQA methods

We evaluated the capabilities of various methods based on Code-Llama-13B, with the experimental results presented in Table 3. The experimental results indicate that the Code-Llama-13B model, enhanced with KGT, consistently surpasses competing methods across all metrics assessed. Notably, KG-GPT improves the F1 score by 15.7% over previous methods CoT&ICL, while our method KGT increases the F1 score by 33% over KG-GPT. Because KG-GPT overlooks the impact of entity types and attributes on answers within the biomedical domain. This achievement positions our approach as a pioneering benchmark in biomedical KGQA, eclipsing previously established best practices.

Comparative analysis across diverse LLMs

We presents a comparative study of KGT applied to Code-Llama-13B against two highly capable LLMs in the general and biomedical domains, with experimental results displayed in Table 4. Code-Llama-13B, enhanced by KGT, significantly outperforms its peers, achieving the highest marks in every assessment metric: a GPT-4 Eval score of 92.4, a BERTScore of 97.7, and a ROUGE F1-score of 86.8. Remarkably, our approach's F1 score surpasses that of ChatGPT-3.5 with the Base method by 52.7%, the CoT&ICL method by 36.3%, and Taiyi's base model by 67.3%. These results highlight KGT's substantial contribution to improving the performance of large language models for the pan-cancer KGQA task. Even when integrated with open-source general models, KGT exhibits remarkable performance, outstripping both the recognized state-of-the-art closed-source large language models and those specifically tailored for the biomedical domain. This showcases KGT's adeptness at parsing and leveraging knowledge graph data, setting a new standard for future research and applications in the field.

Assessing KGT's effectiveness on diverse LLM platforms

To underscore the adaptability and effectiveness of our KGT framework when applied to a range of large language models, we conduct experiments on several LLMs: Zephyr [44], Llama-2 [2], and Code-Llama [42]. The outcomes, illustrated in Fig. 3, reveal that while the CoT&ICL techniques significantly boost performance in terms

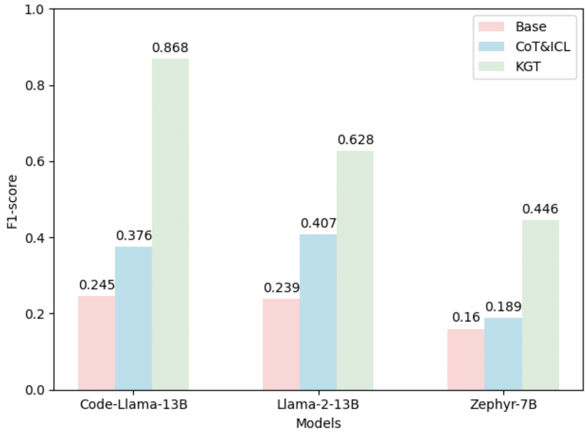

Figure 3. Performance of various models using different strategies.

of F1-score, our KGT methodology delivers even more substantial enhancements across all evaluated models. This demonstrates not only the effectiveness of CoT&ICL as a performance-enhancing strategy but also highlights the superior advancements and impact of KGT, establishing its dominance and efficiency in knowledge graph question-answering tasks.

Ablation study for dissecting the components of KGT

In our effort to illuminate the individual contributions of the components that constitute our KGT framework and their collective impact on enhancing the performance of LLMs, we define four foundational modules: (1) question analysis for the extraction of pivotal information, (2) graph schema-based inference to identify the optimal relational chains in the knowledge graph, (3) the generation of query statements to facilitate subgraph construction, and (4) the inference process coupled with the articulation of results in natural language. This ablation study, grounded on the Code-Llama-13B model, is meticulously designed to evaluate the efficacy of these components. Since graph schema-based inference requires the process of question analysis, the question analysis module cannot be removed in isolation; simultaneously, subgraph construction is indispensable for knowledge graph retrieval. If the subgraph construction module is independently omitted, the outputs of the initial two modules will not impact the final results, making the isolated exclusion of this component illogical. Therefore, we introduce three specific ablated configurations for examination: (1) excluding graph schema-based inference (*w/o* GSBI), (2) omitting both ques-

Table 5. Ablation study of the KGT framework under Code-Llama-13B.

Method	GPT-4 Eval (%)	BERTScore (%)	ROUGE (%)		
			Recall	Precision	F1-score
KGT(ours)	92.4	97.7	87.4	87.7	86.8
w/o GSBI	71.8	95.5	68.1	69.8	66.8
w/o QA&GSBI	69.7	94.7	55.0	66.3	58.2
w/o QA&GSBI&SC	24.7	77.4	14.8	12.3	12.2

tion analysis and graph schema-based inference (*w/o* QA&GSBI), and (3) removing question analysis, graph schema-based inference, and subgraph construction (*w/o* QA&GSBI&SC), effectively bypassing the structured query of the SOKG and relying solely on the LLM's inherent knowledge for question answering.

The results of the ablation study, as shown in Table 5, demonstrate that when we remove the GSBI, we observe a 20% decrease in the F1 score. Removing both GSBI and QA results in an additional 8.6% decrease in the F1 score compared to removing GSBI alone. Furthermore, removing GSBI, QA, and SC together leads to a 46% decrease in the F1 score compared to removing just GSBI and QA. The experiments reveal that SC is crucial; its absence forces the LLM to rely solely on its inherent knowledge, significantly reducing effectiveness. GSBI is also key, as it aids in navigating complex multi-hop questions by providing necessary intermediate entity information for subgraph construction. QA is equally essential, ensuring accurate identification of entities and properties for correct subgraph construction. All these variants underperform compared to the complete KGT, indicating that each of the three modules is vital for the final performance. Furthermore, such observations confirm that our KGT can indeed leverage knowledge to enhance the final performance of LLMs.

Implementation Settings

Our knowledge graph is quite large, with a complex schema, and typically involves input tokens within 1300. Our experiment does not require fine-tuning, and the inference time is related to the model size and computational resources. For example, when using our method, KGT, with the Code-Llama-13B model on an 80GB A100 GPU, it occupies 33GB of VRAM. Without any acceleration frameworks, the inference requires four passes, each taking around 20 seconds.

Case studies

Drug repositioning

Drug repositioning emerge as a promising strategy to accelerate the process of drug development. This approach involves identifying new therapeutic uses for existing drugs, thereby saving time and resources typically required for bringing a new drug to market [45]. Our system is capable of investigating the potential repositioning of carteolol for the treatment of hemangiomas. The example is shown in Supplementary Material Table.S2 and relational diagram is shown in Fig. 4(A). Utilizing the system's knowledge graph, a relational chain is delineated, illustrating that propranolol, another inhibitor of ADRB1, is effectively employed in the treatment of hemangiomas. The system harnesses this insight to formulate a hypothesis that carteolol, by virtue of its similar mechanism of inhibition, could be potentially repositioning for treating hemangiomas [46]. This hypothesis would serve as a precursor to clinical trials and research, potentially expediting the availability of an additional therapeutic option for hemangiomas patients.

Drug resistance research

Drug resistance in cancer treatment poses a significant challenge in clinical oncology. Understanding the genetic basis of resistance can lead to more effective treatment strategies and personalized medicine approaches. Research in drug resistance involves determining why certain cancer carrying mutated gene are not responsive to specific drugs and finding ways to overcome this resistance [47]. Our system is capable of exploring drug resistance in cancer. The example is shown in Supplementary Material Table.S3 and relational diagram is shown in Fig. 4(B). The KG data indicates that the ALK-p.L1196M mutation, which is associated with gastric cancer, has a known resistance to Nalatinib [48, 49]. The LLM processes this information and infers that due to this resistance, Nalatinib might not be an effective medication for treating cancers caused by the ALK-p.L1196M mutation. The case highlights the critical importance of understanding specific gene-drug interactions in drug resistance research. It demonstrates how certain gene mutations could render a drug ineffective, which in turn could guide oncologists in choosing alternative treatments or developing new drugs that can bypass or target the resistance mechanisms. By accelerating the process of understanding drug resistance, these AI-driven systems can contribute to improved patient outcomes and the optimization of cancer treatment protocols.

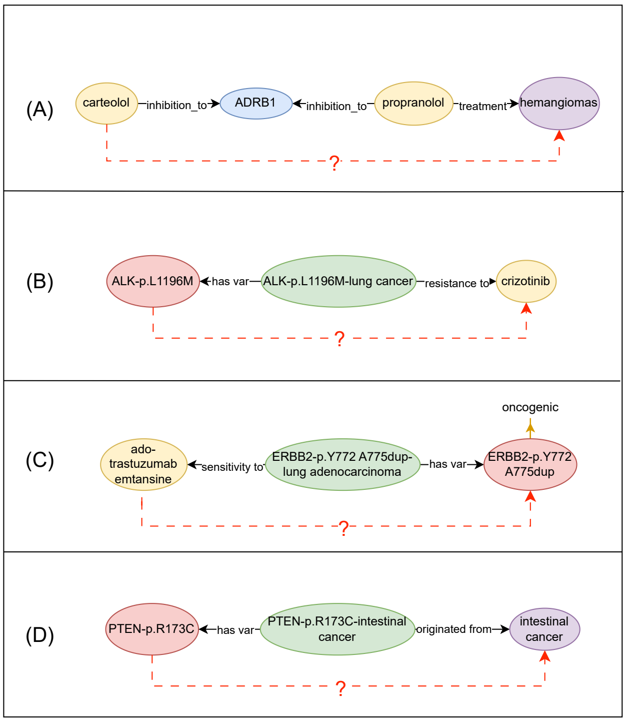

Figure 4. (A), (B), (C) and (D) respectively represent the relational diagrams of drug repositioning, drug resistance research, individualized treatmen and selection and understanding of biomarkers.

Individualized treatment

Details on individualized treatment are provided in Supplementary Material Case studies A. It is important to note that this example is included solely to illustrate the technical capabilities of the proposed method. The output generated in this example has not been validated for clinical use, and further validation in clinical settings would be required before any such application.

Selection and understanding of biomarkers

Details on selection and understanding of biomarkers are provided in Supplementary Material Case studies B.

Discussion

In this paper, we introduce a novel framework KGT, which employs LLMs for reasoning on the schema of KGs, to enhance the reasoning abilities of LLMs in areas with missing domain data by utilizing domain-specific knowledge graphs, such as oncology knowledge graphs, thereby addressing the issue of factual hallucinations in LLMs. Our method excels in extracting, validating, and refining factual knowledge throughout the LLMs' reasoning process. It seamlessly integrates with various LLMs, including open-source models like Code-Llama, and enhances the capabilities of LLMs solely through prompt engineering and in-context learning, without any fine-tuning. This grants it significant generalizability.

We possess an extensive oncology knowledge graph and have established a benchmark based on it to evaluate the capabilities of various methods. When tested on PcQA using various open-source LLMs, the KGT framework performs exceptionally well, surpassing the current best methods by 33%. This significant improvement positions our approach as a pioneering benchmark in biomedical KGQA, setting a new standard that advances beyond previously established best practices. Additionally, through case studies, our approach has been shown to effectively provide therapeutic plans, generate valuable hypotheses for drug repositioning, identify potential drug targets, and study drug resistance. This underscores the practical value of the KGT framework in delivering insightful contributions that aid in the development and optimization of treatment strategies. Each case study's conclusions are further validated by evidence from previously published research papers, enhancing the credibility and impact of our findings.

However, it is important to note that the constructed QA dataset and the corresponding published subset of the SmartQuerier Oncology Knowledge Graph (SOKG) were specifically designed to validate the effectiveness of the KGT framework within this study. While the dataset is highly relevant to biomedical applications, its scope is primarily focused on validating the proposed method. Therefore, it may not cover all potential use cases. Additionally, our system currently has the drawback of not performing fuzzy matching; if a drug name is misspelled by even one letter, it fails to retrieve information from the knowledge graph. Therefore, we plan to improve this aspect in the future to enhance the system's usability and reliability. Our ultimate goal is to create a robust framework applicable to the rapidly evolving domain of medical knowledge, supporting healthcare professionals in delivering personalized, precise medication tailored to the individual needs of each patient.

Finally, we affirm that this study serves as a proof-of-concept, aiming to showcase the technical feasibility and initial efficacy of the method, which has not been validated in actual clinical practice. In any clinical or medical decision-making, reliance should always be placed on the judgment and guidance of professional healthcare practitioners.

Availability of Source Code and Requirements

Project name: bioKGQA-KGT

- Project homepage: https://github.com/yichun10/bioKGQA-KGT.git.
- Operating system(s): Linux (Ubuntu)
- Resource usage in inference step: A Linux (Ubuntu) system with at least 2 CPU cores and 32 GB of VRAM. The GPU card needs at least 60GB VRAM(either two 32GB V100s or one 80GB A100).
- Programming language: Shell Script (Bash) with Python 3.10.13
- Other requirements: Python 3.10.13 with GPU/CPU support, neo4j 5.13.0 (please see more requirements on Github repository).
- Licenses: MIT license
- Research Resource Identifier (#RRID): SCR_025176

Data Availability

We have publicly provided a subset of the SmartQuerier Oncology Knowledge Graph necessary for reproducing the research. An archival copy of the code and the subgraph of the Knowledge Graph used in this research is available via Software Heritage [33], and the code and datasets can be accessed via GitHub [50]. Additionally, the prompts used in interactions with LLMs [1, 2, 38, 42, 43, 44] during this research are available in the supplemental material. For access to the complete SmartQuerier Oncology Knowledge Graph data, please contact at service@smartquerier.com.

Supplementary material

Supplementary material is available at Supplementary material.pdf.

Abbreviations

KG: knowledge graph; LLM: large language model; NLP: natural language processing; SFT: supervised fine-tuning; RLHF: reinforcement learning with human feedback; CF: catastrophic forgetting; CoT: Chain-of-thought; APE: automatic prompt engineer; KGQA: knowledge graph question answering; BFS: breadth-first search; PcQA: Pan-cancer Question Answering; ICL: in-context learning; GPT: generative pre-trained transformer.

Competing Interests

Author Chao Ma is employed by Smartquerier Gene Technology (Shanghai) Co., a company active in the biomedical field relevant to the content of this research. The SmartQuerier Oncology Knowledge Graph (SOKG) used in this study is proprietary to Smartquerier Gene Technology (Shanghai) Co. The other authors declare that they have no competing interests.

Ethical Statement

This study involves the generation of a biomedical question-answer dataset derived from a biomedical knowledge graph developed by our team. The knowledge graph has been meticulously constructed using non-personalized data obtained from various credible biomedical sources. The data collection and utilization processes strictly comply with all relevant legal regulations and ethical guidelines, ensuring the highest standards of data security and privacy. The dataset adheres rigorously to data protection principles and contains no sensitive personal information or identifiable individual health data. Furthermore, as the data collection and processing activities in this study do not involve human subjects, this research did not require ethical review or approval.

Funding

This work was supported in part by funds from the National Key R&D Program (No. 2022YFF1202101, 2023YFC3041600); the CAS Research Fund (No. XDB38050200); the Self-supporting Program of Guangzhou National Laboratory (No. SRPG22001 and SRPG22007)

Authors' Contributions

Y.F. and L.Z. conceived the project. Y.F. proposed a KGQA benchmark, developed the KGT framework, implemented the code, conducted the experiments, and drafted the manuscript. C.M. contributed the SmartQuerier Oncology Knowledge Graph. Y.L. and L.Z. supervised the study. All authors read and approved the final manuscript.

References

1. OpenAI, ChatGPT (Nov 30 version) [Large language model]; 2022. https://chat.openai.com/chat.
2. Touvron H, Martin L, Stone K, Albert P, Almahairi A, Babaei Y, et al. Llama 2: Open foundation and fine-tuned chat models [Large language model]. arXiv preprint arXiv:230709288 2023;.
3. Ji Z, Lee N, Frieske R, Yu T, Su D, Xu Y, et al. Survey of hallucination in natural language generation. ACM Computing Surveys 2023;55(12):1–38.
4. Liu T, Zheng X, Chang B, Sui Z. Towards faithfulness in open domain table-to-text generation from an entity-centric view. In: Proceedings of the AAAI Conference on Artificial Intelligence, vol. 35; 2021. p. 13415–13423.
5. Kang D, Hashimoto T. Improved natural language generation via loss truncation. arXiv preprint arXiv:200414589 2020;.
6. Pan S, Luo L, Wang Y, Chen C, Wang J, Wu X. Unifying large language models and knowledge graphs: A roadmap. IEEE Transactions on Knowledge and Data Engineering 2024;.
7. Han T, Adams LC, Papaioannou JM, Grundmann P, Oberhauser T, Löser A, et al. MedAlpaca–An Open-Source Collection of Medical Conversational AI Models and Training Data. arXiv preprint arXiv:230408247 2023;.
8. Yunxiang L, Zihan L, Kai Z, Ruilong D, You Z. Chatdoctor: A medical chat model fine-tuned on llama model using medical domain knowledge. arXiv preprint arXiv:230314070 2023;.
9. Singhal K, Azizi S, Tu T, Mahdavi SS, Wei J, Chung HW, et al. Large language models encode clinical knowledge. arXiv preprint arXiv:221213138 2022;.
10. Wang H, Liu C, Xi N, Qiang Z, Zhao S, Qin B, et al. Huatuo: Tuning llama model with chinese medical knowledge. arXiv preprint arXiv:230406975 2023;.
11. Xiong H, Wang S, Zhu Y, Zhao Z, Liu Y, Wang Q, et al. Doctorglm: Fine-tuning your chinese doctor is not a herculean task. arXiv preprint arXiv:230401097 2023;.
12. Zhang H, Chen J, Jiang F, Yu F, Chen Z, Li J, et al. HuatuoGPT, towards Taming Language Model to Be a Doctor. arXiv preprint arXiv:230515075 2023;.
13. Yang S, Zhao H, Zhu S, Zhou G, Xu H, Jia Y, et al. Zhongjing: Enhancing the chinese medical capabilities of large language model through expert feedback and real-world multi-turn dialogue. arXiv preprint arXiv:230803549 2023;.
14. Tian Y, Gan R, Song Y, Zhang J, Zhang Y. ChiMed-GPT: A Chinese Medical Large Language Model with Full Training Regime and Better Alignment to Human Preferences. arXiv preprint arXiv:231106025 2023;.
15. Ouyang L, Wu J, Jiang X, Almeida D, Wainwright C, Mishkin P, et al. Training language models to follow instructions with human feedback. Advances in Neural Information Processing Systems 2022;35:27730–27744.
16. Luo Y, Yang Z, Meng F, Li Y, Zhou J, Zhang Y. An empirical study of catastrophic forgetting in large language models during continual fine-tuning. arXiv preprint arXiv:230808747 2023;.
17. Li Z, Hoiem D. Learning without forgetting. IEEE transactions on pattern analysis and machine intelligence 2017;40(12):2935–2947.
18. Liu V, Chilton LB. Design guidelines for prompt engineering text-to-image generative models. In: Proceedings of the 2022 CHI Conference on Human Factors in Computing Systems; 2022. p. 1–23.
19. Wei J, Wang X, Schuurmans D, Bosma M, Xia F, Chi E, et al. Chain-of-thought prompting elicits reasoning in large language models. Advances in Neural Information Processing Systems 2022;35:24824–24837.
20. Zhou Y, Muresanu AI, Han Z, Paster K, Pitis S, Chan H, et al. Large language models are human-level prompt engineers. arXiv preprint arXiv:221101910 2022;.
21. Sun H, Dhingra B, Zaheer M, Mazaitis K, Salakhutdinov R, Cohen WW. Open domain question answering using early fusion of knowledge bases and text. arXiv preprint arXiv:180900782 2018;.
22. Sun H, Bedrax-Weiss T, Cohen WW. Pullnet: Open domain question answering with iterative retrieval on knowledge bases and text. arXiv preprint arXiv:190409537 2019;.
23. Zhang J, Zhang X, Yu J, Tang J, Tang J, Li C, et al. Subgraph retrieval enhanced model for multi-hop knowledge base question answering. arXiv preprint arXiv:220213296 2022;.
24. Chen Y, Wu L, Zaki MJ. Bidirectional attentive memory networks for question answering over knowledge bases. arXiv preprint arXiv:190302188 2019;.
25. Saxena A, Tripathi A, Talukdar P. Improving multi-hop question answering over knowledge graphs using knowledge base embeddings. In: Proceedings of the 58th annual meeting of the association for computational linguistics; 2020. p. 4498–4507.
26. Lan Y, He G, Jiang J, Jiang J, Zhao WX, Wen JR. A survey on complex knowledge base question answering: Methods, challenges and solutions. arXiv preprint arXiv:210511644 2021;.
27. Das R, Zaheer M, Thai D, Godbole A, Perez E, Lee JY, et al. Case-based reasoning for natural language queries over knowledge bases. arXiv preprint arXiv:210408762 2021;.
28. Jiang J, Zhou K, Zhao WX, Wen JR. Unikgqa: Unified retrieval and reasoning for solving multi-hop question answering over knowledge graph. arXiv preprint arXiv:221200959 2022;.
29. Jiang J, Zhou K, Dong Z, Ye K, Zhao WX, Wen JR. Structgpt: A general framework for large language model to reason over structured data. arXiv preprint arXiv:230509645 2023;.
30. Sun J, Xu C, Tang L, Wang S, Lin C, Gong Y, et al. Think-on-Graph: Deep and Responsible Reasoning of Large Language Model on Knowledge Graph. In: The Twelfth International Conference on Learning Representations;. .
31. LUO L, Li YF, Haf R, Pan S. Reasoning on Graphs: Faithful and Interpretable Large Language Model Reasoning. In: The Twelfth International Conference on Learning Representations;. .
32. Kim J, Kwon Y, Jo Y, Choi E. KG-GPT: A general framework for reasoning on knowledge graphs using large language models. arXiv preprint arXiv:231011220 2023;.
33. Feng Y, Zhou L, Ma C, Zheng Y, He R, Li Y, Knowledge Graph-based Thought: a knowledge graph enhanced LLMs framework for pan-cancer question answering (Version 1); 2024. [Computer software]. https://archive.softwareheritage.org/swh:1:dir:4d5d3acbd7784d97229a0a5ba0453f67f73ed6cf;origin=https://github.com/yichun10/bioKGQA-KGT;visit=swh:1:snp:1906dbbfc88c9d1c8b7acf7deb7495e8002cbafa;anchor=swh:1:rev:9a0244de046118fb6d2423912fd0b34df7fd052c.
34. Wang J, Wu M, Huang X, Wang L, Zhang S, Liu H, et al. SynLethDB 2.0: a web-based knowledge graph database on synthetic lethality for novel anticancer drug discovery. Database 2022;2022:baac030.
35. Zhu C, Yang Z, Xia X, Li N, Zhong F, Liu L. Multimodal reasoning based on knowledge graph embedding for specific diseases. Bioinformatics 2022;38(8):2235–2245.
36. Zhang Y, Dai H, Kozareva Z, Smola A, Song L. Variational reasoning for question answering with knowledge graph. In: Proceedings of the AAAI conference on artificial intelligence,

vol. 32; 2018. .

37. Kim J, Park S, Kwon Y, Jo Y, Thorne J, Choi E. FactKG: Fact verification via reasoning on knowledge graphs. arXiv preprint arXiv:230506590 2023;.
38. Achiam J, Adler S, Agarwal S, Ahmad L, Akkaya I, Aleman FL, et al. GPT-4 Technical Report (Mar 14 version) [Large language model]. arXiv preprint arXiv:230308774 2023;.
39. Zhang T, Kishore V, Wu F, Weinberger KQ, Artzi Y. Bertscore: Evaluating text generation with bert. arXiv preprint arXiv:190409675 2019;.
40. Lin CY. Rouge: A package for automatic evaluation of summaries. In: Text summarization branches out; 2004. p. 74–81.
41. Dong Q, Li L, Dai D, Zheng C, Wu Z, Chang B, et al. A survey for in-context learning. arXiv preprint arXiv:230100234 2022;.
42. Roziere B, Gehring J, Gloeckle F, Sootla S, Gat I, Tan XE, et al. Code llama: Open foundation models for code [Large language model]. arXiv preprint arXiv:230812950 2023;.
43. Luo L, Ning J, Zhao Y, Wang Z, Ding Z, Chen P, et al. Taiyi: a bilingual fine-tuned large language model for diverse biomedical tasks [Large language model]. arXiv preprint arXiv:231111608 2023;.
44. Tunstall L, Beeching E, Lambert N, Rajani N, Rasul K, Belkada Y, et al., Zephyr: Direct Distillation of LM Alignment [Large language model]; 2023.
45. He S, Liu X, Ye X, Tetsuya S. Analysis of Drug Repositioning and Prediction Techniques: A Concise Review. Current Topics in Medicinal Chemistry 2022;22(23):1897–1906.
46. Gan Lq, Wang H, Ni Sl, Tan Ch. A prospective study of topical carteolol therapy in Chinese infants with superficial infantile hemangioma. Pediatric Dermatology 2018;35(1):121–125.
47. Gottesman MM. Mechanisms of cancer drug resistance. Annual review of medicine 2002;53(1):615–627.
48. Alshareef A, Zhang HF, Huang YH, Wu C, Zhang JD, Wang P, et al. The use of cellular thermal shift assay (CETSA) to study Crizotinib resistance in ALK-expressing human cancers. Scientific reports 2016;6(1):33710.
49. Simionato F, Frizziero M, Carbone C, Tortora G, Melisi D. Current strategies to overcome resistance to ALK-inhibitor agents. Current drug metabolism 2015;16(7):585–596.
50. Feng Y, Zhou L, Ma C, Zheng Y, He R, Li Y, bioKGQA-KGT: Knowledge Graph-based Thought; 2024. `https://github.com/yichun10/bioKGQA-KGT`.

Click here to access/download
Supplementary Material
Knowledge Graph-based Thought_Supplementary material.pdf
