# Supplementary material for: Knowledge graph–based thought: a knowledge graph–enhanced LLM framework for pan-cancer question answering
Source: Gigascience. 2025 Jan 6;14:giae082. doi: 10.1093/gigascience/giae082 (PMC11702363; doi:10.1093/gigascience/giae082)
Supplement: giae082_Response_to_Reviewer_Comments_evision_4 [file giae082_response_to_reviewer_comments_evision_4.pdf]

Author's Response To Reviewer Comments

Thanks for your suggestion. We have added the ORCIDs for the authors to the title page. The revised LaTeX source files, including the manuscript, bibliography, style files, and figures, have been uploaded to the Editorial Manager as requested.
