# Supplementary material for: Knowledge graph–based thought: a knowledge graph–enhanced LLM framework for pan-cancer question answering
Source: Gigascience. 2025 Jan 6;14:giae082. doi: 10.1093/gigascience/giae082 (PMC11702363; doi:10.1093/gigascience/giae082)
Supplement: giae082_Response_to_Reviewer_Comments_Original_Submission [file giae082_response_to_reviewer_comments_original_submission.pdf]

Author's Response To Reviewer Comments

Revision Notes for GIGA-D-24-00191

The authors would like to thank editor and reviewers for their constructive comments and suggestions on our submission. We have addressed the comments and modified the manuscript accordingly. In this revised version, changes to our manuscript within the document have been highlighted by using blue colored text. Please refer to the file “Revision_highlight_Knowledge Graph-based Thought.pdf” in the supplementary materials. Point-by-point responses to the editor and reviewers are listed below.

Response to EIC:
#Q1: Please pay particular attention to the questions of reviewer 1 regarding details of the methodological procedures.
Response: Thanks for your suggestion. We have addressed Reviewer 1's concerns regarding methodological details with detailed responses and additional evidence in the revised manuscript.

Response to Reviewer #1:
#Q1: This paper construct a new dataset denoted as PcQA form a customized KG called SOKG for the evaluation of pan-cancer question answering. This is a great contribution to the community. However, it is unclear how to constuct such a dataset. More details about the construnction process and statistics of the final datasets should be disscussed in the paper. For example, how to generate the natural language questions and answers? How to link the question with relatived KG information (i.e., entity and relation)? How many questions can be answered by the KGs (i.e., answer converage rate). How many questions have been generated? What is the ratio of each quetion types defined in Table 2?
Response: Thanks for your suggestion.
(1)We have detailed our dataset construction method (lines 205 - lines 208): first, we used GPT-4 to generate a portion of the questions and answers based on SOKG. These were then manually annotated and filtered to select appropriate QA pairs, followed by further manual expansion to enrich the dataset.
(2) The first step in our method (KGT) is Question Analysis, which involves identifying and linking relevant entities. The second step, Graph Schema-based inference, is used to deduce the relationships between these entities. We do not assume that entities and relationships are explicitly marked in the questions; rather, they are determined through our analysis process.
(3) Since the generated QA pairs are based on SOKG, and SOKG contains all the answers, the knowledge graph is capable of answering all the generated questions.
(4) We have added the count of each question type in Table 2 on Pages 4.

#Q2: In Table2, the author define 4 reasoning types. How about other reasoning types such union and negation? Can we incorpate these types into the datasets?
Response: Thanks for your suggestion. In the biomedical domain, questions like "Is carteolol effective in treating hemangioma?" are more common. The key difference between this type of yes/no question and a negation question, such as "Is carteolol ineffective in treating hemangioma?" lies in the final reasoning step. Although the LLM can correctly answer either type of question when the relational chain is provided, we have chosen not to include negation questions at this time. Similarly, in the biomedical field, intersection questions, such as "How to treat cholangiocarcinoma carrying FGFR2?" are more common. While intersection and union questions differ only in the final reasoning step, our dataset focuses on intersection questions and does not include union questions. In future research, we plan to incorporate negation and union questions to enhance the diversity of the dataset.

#Q3: The propsed method is novel and interesting. However some details are unclear. In the candidate path search, do we want to search reasoning paths or relational chains? The definition of these two paths are also unclear. Please give clear definition of them in prelimary. If is the reasoning paths, do we only keep the type information during the BFS?
Response: Thanks for your suggestion. We have provided clear definitions in the paper (lines 120 - lines 123). In the candidate path search, the paths being searched refer to relational paths, which are the relational chains in the knowledge graph schema constructed in the previous step. These paths consist of

the types of entities and the relationships between these types. For example: (Genesymbol) <-(inhibition_to)- (Drug). During BFS, we only retain the information of the entities' types and the relationships between these types (lines 258 - lines 259).

#Q4: I do not understand why we need to generatea cypher query to retrieve subgraph then construct relation paths from KG. We can directly retrieved relational paths from KGs by BFS. What are the benefits and motivations of using this two-stage pipeline?
Response: Thanks for your suggestion. Cypher and BFS are both fundamentally querying methods, with Cypher being a structured query language. There are several advantages to generating Cypher queries for retrieving subgraphs before constructing relational paths:
(1)Efficiency in Complex Biomedical Knowledge Graphs: Biomedical knowledge graphs are typically large and complex, and are often managed within graph databases like Neo4j. Cypher is the query language for these graph databases, and databases often optimize heavily for Cypher queries. By using Cypher, the query can leverage built-in indexes and optimizers within the database, enabling efficient retrieval of relational paths.
(2)Readability and Maintainability: Cypher queries are relatively readable and easier to understand for those familiar with graph databases. Using Cypher provides a declarative approach to expressing complex graph structures and paths, making it easier to maintain and comprehend the queries.
(3)Simplification of Complex Operations: When retrieving complex relational chains that might require multiple steps, Cypher can simplify these operations through its declarative nature, reducing the complexity of the code.

#Q5: What are the meanings of the X and √ in the figure. How to get them?

Response: Thanks for your suggestion. We have explained the meanings of the X and √ symbols in the caption of Figure 2 on Pages 5. For example, in the Candidate Path Search: BFS is used to find all possible paths that match the head and tail entity types, which may result in three potential paths as shown. In the Optimal Path Selection: the LLM uses similarity matching to recall the path most relevant to the question (marked with √), which is the correct path, and excludes the incorrect paths (marked with X). The most relevant path is then used as the input for the next reasoning step.

#Q6: In experiments, other advanced KGQA methods can be compared, e.g., RoG and ToG.
Response: Thanks for your suggestion. We have already compared our method with advanced KGQA approaches, such as KG-GPT, in the experimental section. Additionally, we have included a discussion in the Introduction highlighting the differences between our approach and RoG and ToG (lines 74 - lines 78). Think-on-Graph (ToG) introduces iterative exploration of the knowledge graph, which can become inefficient with very large KGs. Additionally, RoG necessitates fine-tuning to accurately generate and plan the relation paths.

#Q7: The analysis of used token, time, and cost should be disscussed in the paper.
Response: Thanks for your suggestion. We have added a section titled "Inference Time and Computational Cost" in the experimental part of the paper to discuss the analysis of used tokens, time, and cost (lines 412 - lines 419). Our knowledge graph is quite large, with a complex schema, and typically involves input tokens within 1300. Our experiment does not require fine-tuning, and the inference time is related to the model size and computational resources. For example, when using our method, KGT, with the Code-Llama-13B model on an 80GB A100 GPU, it occupies 33GB of VRAM. Without any acceleration frameworks, the inference requires four passes, each taking around 20 seconds.

#Q8: Can we apply the proposed method to other KGs (i.e., SynLethKG, and SDKG) or KGQA tasks (MetaQA, and FACTKG) to show the generability.
Response: Thanks for your suggestion. The biomedical knowledge graphs SynLethKG and SDKG do not include a question-answering dataset. To demonstrate the versatility of our method, we manually designed several QA pairs based on SDKG (the data for this knowledge graph is available at https://github.com/ZhuChaoY/SDKG-11/tree/main/Dataset) and tested them using our approach (KGT). The test results are as follows:
(1)"question": "What types of disease can be treated with Chloroquine?",
"answer": "Output: Chloroquine can treat autoimmune disease and rheumatoid arthritis."

(2)"question": "What is the relationship between Oxaliplatin and HEPATOCELLULAR CARCINOMA?",
"answer": " Output: Oxaliplatin is a treatment for hepatocellular carcinoma."
(3)"question": " What drugs can affect Cutanese Melanoma?",
"answer": " Output: Cutaneous Melanoma can be affected by the following drugs: 5'-S-methyl-5'-thioadenosine."
(4)"question": "What is the relationship between MALAT1 and BODY MASS INDEX QUANTITATIVE TRAIT LOCUS 20?",
"answer": " Output: MALAT1 indirectly affects the disease phenotype of BODY MASS INDEX QUANTITATIVE TRAIT LOCUS 20 by inhibiting E-cadherin."
Additionally, we created a webpage using Gradio and recorded a test video. The examples have been uploaded at https://github.com/yichun10/bioKGQA-KGT/tree/main?tab=readme-ov-file#sdkg-demo.

Response to Reviewer #2:
#Q1: The use of LLMs for code generation and interpretation within the same request is also innovative. The application of your framework to MeSH (https://www.nlm.nih.gov/mesh/meshhome.html) and other broader linked ontologies would be very interesting. You might also consider integrating tool calling as well (which in a way you are with subgraphs), to either further reduce the demential space or accessing data that does not otherwise have a graph structure. In this case, the content of your subgraph nodes might be the result of a function call.
Response: Thank you for your valuable feedback and for recognizing our contribution in bridging LLMs and structured data. We appreciate your suggestion regarding the application of our framework to MeSH and other broader linked ontologies, and we will certainly consider this direction in our future work. Additionally, we find your idea of integrating tool calling very promising and will explore this further, particularly in cases where subgraph nodes could be the result of function calls.
