# Supplementary material for: Knowledge graph–based thought: a knowledge graph–enhanced LLM framework for pan-cancer question answering
Source: Gigascience. 2025 Jan 6;14:giae082. doi: 10.1093/gigascience/giae082 (PMC11702363; doi:10.1093/gigascience/giae082)
Supplement: giae082_Response_to_Reviewer_Comments_Revision_1 [file giae082_response_to_reviewer_comments_revision_1.pdf]

Author's Response To Reviewer Comments

The authors would like to thank editor and reviewers for their constructive comments and suggestions on our submission. We have addressed the comments and modified the manuscript accordingly. In this revised version, changes to our manuscript within the document have been highlighted by using blue colored text. Please refer to the file “Revision_highlight_Knowledge Graph-based Thought.pdf” in the supplementary materials. Point-by-point responses to the editor and reviewers are listed below.

Response to EIC:
#Q1: I'd like to emphasize that I agree with the reviewer that it's crucial to evaluate and consider the correctness of the GPT-based outputs; and to make sure that readers of your article use the output responsibly and fully understand limitations, such as potential factual errors. As the reviewer suggests, this may also need some further discussion in the manuscript, also considering ethical aspects how this method and the answers it produces should (and should not) be used and interpreted.
Response: Thank you for your suggestion. We have expanded the manuscript to include more detailed information on the dataset construction process and have incorporated a formal ethical statement to ensure clarity regarding the ethical standards and data handling procedures associated with the study.

Response to Reviewer #1:
#Q1: Dataset construction and evaluation: The author adopts GPT-4 to generate the question-answer pairs. Is there any quality control method to ensure or evaluate the quality of the generated pairs? Has the author asked any medical experts to check the correctness of the generated datasets? Considering the medical-related nature of the dataset, it would be better to include an ethical statement in the paper.
Response: Thanks for your suggestion.
(1)We have added more detailed explanations of the data construction process in the manuscript (lines 205 - lines 214). Our questions were carefully crafted by experts based on the content of the knowledge graph. GPT-4 was then employed to generate Cypher queries, which were used to retrieve answers from the knowledge graph. The generated Cypher queries and corresponding answers underwent an initial review by a biomedical PhD candidate, who manually verified and corrected the dataset against the knowledge graph. Finally, the entire dataset was thoroughly reviewed by two biomedical experts to ensure its accuracy and reliability. This multi-step process was meticulously designed to uphold the highest standards of quality throughout the dataset creation.

(2)We have addressed this concern by adding an ethical statement to the paper (lines 541 - lines 552): This study involves the generation of a biomedical question-answer dataset derived from a proprietary biomedical knowledge graph developed by our team. The knowledge graph has been meticulously constructed using non-personalized data obtained from various credible biomedical sources. The data collection and utilization processes strictly comply with all relevant legal regulations and ethical guidelines, ensuring the highest standards of data security and privacy. The dataset adheres rigorously to data protection principles and contains no sensitive personal information or identifiable individual health data. Furthermore, as the data collection and processing activities in this study do not involve human subjects, this research did not require ethical review or approval.

#Q2: The author states that the Cypher query-based retrieval and BFS-based retrieval are complementary. It would be beneficial to include an ablation study to compare them.
Response: Thanks for your suggestion. Our knowledge graph is stored in Neo4j, where Cypher serves as the native query language, specifically optimized for seamless integration with the Neo4j graph database. Cypher queries are not only highly readable but also leverage the database's built-in optimizations, such as automatic index utilization. For instance, when querying nodes based on indexed properties, Cypher uses these indexes to quickly retrieve relevant data, which BFS may struggle with due to its exhaustive search nature, potentially leading to inefficiencies in large KGs. This results in more efficient operations for complex graph retrievals. Given our current application scenario, BFS-based retrieval is not suitable, but we will consider a more detailed comparison of these two methods in future work.
