# Supplementary material for: Knowledge graph–based thought: a knowledge graph–enhanced LLM framework for pan-cancer question answering
Source: Gigascience. 2025 Jan 6;14:giae082. doi: 10.1093/gigascience/giae082 (PMC11702363; doi:10.1093/gigascience/giae082)
Supplement: giae082_Response_to_Reviewer_Comments_Revision_2 [file giae082_response_to_reviewer_comments_revision_2.pdf]

Author's Response To Reviewer Comments

The authors would like to thank editor and reviewers for their constructive comments and suggestions on our submission. We have addressed the comments and modified the manuscript accordingly. In this revised version, changes to our manuscript within the document have been highlighted by using blue colored text. Please refer to the file “Revision_highlight_Knowledge Graph-based Thought.pdf” in the supplementary materials. Point-by-point responses to the editor and reviewers are listed below.

Response to EIC:
#Q1: Please include a statement that this is a "proof-of-concept study" in the abstract, and that the method helps experts to develop hypotheses in a research context.
Response: Thank you for your suggestion. I have added the sentence 'This study serves as a proof-of-concept' to the abstract to clarify that this research is intended to demonstrate the feasibility of the proposed method.

#Q2: Please discuss appropriate use cases as well as limitations in the "discussion" section, along the lines of the reviewer's comment: "The author should include a disclaimer that this is a proof-of-principle study that is not validated for clinical practice, and it is a tool that in its current form helps to develop hypotheses - but these need to be judged by experts. "
Response: Thank you for your suggestion. We have followed the reviewers' suggestions to discuss appropriate application scenarios and limitations, and have added a disclaimer accordingly.

#Q3: While most of your use cases deal with research applications, the use case "treatment plan" may be problematic in light of the above concern. If it is not central for the manuscript, you may consider deleting this use case, or alternatively you may make it very clear in the respective section that this example is only shown for illustrating the technical capabilities of your method, but the output is not validated for clinical use.
Response: Thank you for your suggestion. We have carefully considered your feedback and have decided to retain the "treatment plan" use case. We have revised the relevant section to clearly state that this example is solely intended to demonstrate the technical capabilities of our method, and that the output generated has not been validated for clinical use (lines 469 - lines 473).

#Q4: Regarding the knowledge graph, I have another question: In the footnote on page 4, you indicate that questions&answers and the knowledge graph are available via github (which is under an open licence, as we agreed prior to peer review) , but elsewhere in the paper (e.g. in the added ethics statement) your refer to the "proprietary" knowledge graph. Please clarify access to this data in the "data availability" section - as we said prior to review, at least the subset of the KG data that is needed to reproduce the study needs to be freely available.
Response: Thank you for your suggestion. We understand the importance of clarity regarding the availability of the knowledge graph data. To address this, we have explicitly added a statement in the "Data Availability" section, specifying that we have publicly provided a subset of the SmartQuerier Oncology Knowledge Graph necessary for reproducing the research (lines 535 - lines 537). This subset, along with the questions and answers, is available via GitHub under an open license, as agreed upon prior to peer review. The term "proprietary" in the ethics statement refers to the full, unabridged version of the knowledge graph, which is not publicly available. However, the subset provided is fully sufficient for reproducing the study's results.

#Q5: On a different note, please also include a statement on "Competing Interests" - I see at least one author seems to be affiliated with a commercial enterprise. If this enterprise is active in an area related to the content of the paper, the employment of the author(s) should be explicitly declared in the COI statement .
Response: Thank you for your suggestion. We have included a statement on "Competing Interests" in the manuscript (lines 552 - lines 555). Specifically, we have disclosed that author Chao Ma is employed by Smartquerier Gene Technology (Shanghai) Co., a company active in the biomedical field relevant to the content of this research. The other authors declare that they have no competing interests.

Response to Reviewer #1:
#Q1: Data source transparency: the knowledge graph (SOKG) used in this paper is a proprietary knowledge graph. It is hard to valid the construction process and quality of it. It would be great for author to provide further explanation about the construction process of the KG. What is the data source of this KG. How is the construction process established. What quality control mechanism has been involved? How is the sensitive information checked and filtered during the construction. Does the whole construction process get verified and provided by any ethical review committee?
Response: Thank you for your suggestion.
(1) Regarding the transparency of our data sources. We are happy to provide more details about the construction and data sources of the knowledge graph (SOKG) used in our study:
1) Gene Interaction Database: The gene interaction data is primarily derived from the KEGG database, which provides well-curated pathways and interactions relevant to various biological processes.
2) Drug-Gene Interactions: The drug-gene interaction information is sourced from DrugBank and the Therapeutic Target Database (TTD), both of which are widely recognized for their comprehensive and reliable datasets in pharmacology.
3) Cancer Gene Variants and Drug Efficacy: Evidence-based data on cancer gene variants and their impact on drug efficacy are mainly obtained from reputable guidelines and databases, including the NCCN Guidelines, COSO Guidelines, FDA@Drug, and the PubMed database. Additionally, we incorporate data from major conferences and journal literature, such as those presented at ASCO and WCLC, to ensure the inclusion of the most up-to-date clinical evidence.
4) Cancer Ontology Relationships: The cancer ontology relationship data is primarily sourced from OncoTree, a well-established resource for cancer classification (DOI: 10.1200/CCI.20.00108).
We hope this detailed explanation clarifies the data sources and their integration into our knowledge graph. Should you require further details, we are more than willing to provide additional information.
(2)The construction of the SOKG (Structured Oncology Knowledge Graph) involves a combination of manual curation and data integration. Initially, data from the aforementioned sources undergoes a preliminary entity recognition process (NLP) to filter out cancer-related entities and concepts. These entities are then mapped, organized, and manually reviewed to ensure accuracy before being integrated into a unified knowledge graph schema. The design and methodology of the knowledge graph construction have been detailed in a related patent (Patent Publication Number: CN113836931B, see Attachment 2021113984441.pdf). This patent provides a comprehensive description of the methods used for data integration and the schema design that supports the coherent structure of the knowledge graph. We hope this clarification on the construction process addresses your concerns. If there are any further questions or if more detailed information is required, please let us know.
(3)As mentioned earlier, the data sources for the SOKG (Structured Oncology Knowledge Graph) consist of two main types:
1)Integration of Public Data Sources: A portion of the data comes from publicly available sources, such as gene regulation data from KEGG and drug-target relationship data from the Therapeutic Target Database (TTD). These datasets are parsed and mapped into the schema design framework of the SOKG, ensuring data completeness and authenticity.
2)Evidence-Based Medicine (EBM) Data: Another significant part of the data consists of evidence-based medicine (EBM) information, which is crucial for defining the relationships between hub nodes in the knowledge graph (e.g., CancerCell) and drug efficacy. This data is manually curated and cross-verified to ensure consistency with the original literature. To maintain traceability and transparency, we preserve the original references for each relationship within the knowledge graph as edge attributes, allowing for easy verification and source tracking. It is important to note that our proposed knowledge graph-enhanced LLMs framework does not utilize edge information for inference; hence, these attributes are not included in the dataset provided in the paper.
The SOKG (Structured Oncology Knowledge Graph) does not involve any patient privacy or demographic information. For any potentially sensitive information derived from clinical research literature, we have conducted manual screening and filtering to ensure compliance with ethical standards. Consequently, the construction of SOKG did not require submission to an ethical review board for clinical trials. Nonetheless, we strictly adhere to the guidelines provided by ethical committees to ensure the legality and ethical compliance of both the construction and application of the knowledge graph, thereby maintaining the highest standards of ethical integrity throughout our research.

#Q2: Limitations statement: the constructed QA dataset and corresponding published subset of the SOKG seems only designed to reproduce the proposed studies. It is unclear about the potential impact and limitations of how these datasets can be used for potential. The author should make a clear statement of the usage scenarios and limitations.
Response: Thanks for your suggestion. We acknowledge that the constructed QA dataset and the corresponding published subset of the SmartQuerier Oncology Knowledge Graph (SOKG) were specifically designed to validate the effectiveness of our KGT framework within the context of this study. These datasets are tailored to reflect scenarios relevant to biomedical applications, particularly within the domain of oncology.
However, we recognize that the current dataset's scope is primarily focused on the validation of the proposed method. To address this, we have included a detailed statement in the manuscript that clearly outlines the intended usage scenarios (lines 505 - lines 511).

#Q3: Disclaimer statement: The proposed method is only evaluated on the constructed dataset and is not valid in any other published dataset or clinical practice. The author should include a disclaimer that this is a proof-of-principle study that is not validated for clinical practice, and it is a tool that in its current form helps to develop hypotheses - but these need to be judged by experts.
Response: Thanks for your suggestion. We have incorporated the following disclaimer into our paper according to your suggestion (lines 557 - lines 561): We affirm that this study serves as a proof-of-concept, aiming to showcase the technical feasibility and initial efficacy of the method, which has not been validated in actual clinical practice. In any clinical or medical decision-making, reliance should always be placed on the judgment and guidance of professional healthcare practitioners.
