# Supplementary material for: Knowledge graph–based thought: a knowledge graph–enhanced LLM framework for pan-cancer question answering
Source: Gigascience. 2025 Jan 6;14:giae082. doi: 10.1093/gigascience/giae082 (PMC11702363; doi:10.1093/gigascience/giae082)
Supplement: giae082_Response_to_Reviewer_Comments_Revision_3 [file giae082_response_to_reviewer_comments_revision_3.pdf]

# Author's Response To Reviewer Comments

The authors would like to thank editor and curators for their constructive comments and suggestions on our submission. We [illegible] the comments and modified the manuscript accordingly. In this revised version, changes to our manuscript within the docum[illegible] highlighted by using blue colored text. Please refer to the file “Revision_highlight_Knowledge Graph-based Thought.pdf” in th[illegible] materials. Point-by-point responses to the editor are listed below.

Response to EIC:
#Q1: Please add your new Software Heritage repository as a numbered item to the bibliography, including the (long!) URL be[illegible] is: [xx] Feng Y, Zhou L, Ma C, Zheng Y, He R, Li Y. Knowledge Graph-based Thought: a knowledge graph enhanced LLMs fra[illegible] cancer question answering (Version 1). [Computer software]. Software Heritage 2024
https://archive.softwareheritage.org/swh:1:dir:4d5d3acbd7784d97229a0a5ba0453f67f73ed6cf;origin=https://github.com/yi[illegible] KGT;visit=swh:1:snp:1906dbbfc88c9d1c8b7acf7deb7495e8002cbafa;anchor=swh:1:rev:9a0244de046118fb6d2423912fd0b3[illegible]
# Response: Thanks for your suggestion. I have added the Software Heritage repository to the bibliography as a numbered it[illegible] provided URL.

#Q2: In the data availability section, please cite this new Software Heritage archival repo. For example, write something alo[illegible] "An archival copy of the code and the subgraph of the Knowledge Graph used in this research is available via Software Herita[illegible]
# Response: Thanks for your suggestion. I have updated the data availability section to cite the Software Heritage archival re[illegible] requested (lines 545 - lines 546).

#Q3: Analogous to the example above, please also move the github URL in line 538 as a numbered item to the bibliography, [illegible] reference number. ("Code and datasets are available for access via github [yy]")
# Response: Thanks for your suggestion. I have moved the GitHub URL to the bibliography and cited it by reference number [illegible] (lines 546 - lines 547) .

#Q4: Also in the Data Availability section, please mention that prompts used in the interactions with GPT (and other LLMs, if [illegible] available in the supplemental material, and cite the relevant version of chatGPT / other LLms , using the following example fo[illegible] the LLMs in the bibliography: [xx] OpenAI. ChatGPT (Mar 14 version) [Large language model]. 2024 https://chat.openai.com[illegible] make sure that all LLM prompts that were used in the research are included in the supplemental material.
# Response: Thanks for your suggestion. I have updated the Data Availability section to mention that the prompts used in in[illegible] GPT and other LLMs are available in the supplemental material. I have also added the relevant LLM citations in the bibliograp[illegible] (lines 548 - lines 549).

#Q5: Minor formatting point: Please note that the article format doesn't support footnotes. Please incorporate all other URLs [illegible] text to the bibliography and cite them by number, rather than having footnotes . (e.g. footnote 2 on page 4); for other expla[illegible] please incorporate them in the text.

# Response: Thanks for your suggestion. I will remove all footnotes, incorporate the relevant URLs into the bibliography, and [illegible] accordingly (lines 218).

#Q6: In lines 128-139 (second paragraph of the methods section) I recommend to be clearer that, for this research, only a s[illegible] knowledge graph is used, and this subgraph is available as open data (cite the repository here again); and be clear that the f[illegible] graph is proprietary. Also, when describing the size and features of the full knowledge graph, compared to other databases, I [illegible] using more moderate language, especially considering that the full knowledge graph is not openly available and the claims m[illegible] verified with the data provided. At the moment this section reads a bit like advertising in parts rather than a neutral scientific [illegible] example, "As depicted in Table 1, SOKG boasts an extensive collection of over 3 million entities, which dwarfs the entity coun[illegible] compared knowledge graphs," - instead of "boasts" and "dwarfs", I recommend using "SOKG *includes* an extensive collecti[illegible] million entities, which *is substantially larger than the compared knowledge graphs*", or similar wording.
# Response: Thanks for your suggestion. I have revised the wording to use more neutral language and clarified that only a s[illegible] SOKG was used for this research, with the full knowledge graph being proprietary (lines 128 - lines 142).

#Q7: Minor wording point: In line 110, you write " We will fully open-source this dataset and its accompanying knowledge gr[illegible]
subgraph of the SOKG" - as this is released now, if I understand correctly, you can use the present tense and cite e.g. the Gi[illegible]
# Response: Thanks for your suggestion. I have updated the wording to use the present tense and included the relevant cita[illegible]
repository (lines 110 - lines 111).

#Q8: A minor formatting point: the "disclaimer " is great, but please just incorporate this text within the discussion, rather th[illegible]
section.
# Response: Thanks for your suggestion. I have incorporated the disclaimer into the discussion section as requested (lines 5[illegible]

#Q9: In the supplemental material, there is a Chinese-language PDF which I believe is some sort of patent document - as we[illegible]
language journal, this is not useful to publish alongside the paper. But you may cite an English source for this patent, if you f[illegible]
appropriate.
# Response: Thanks for your suggestion. The Chinese-language PDF is indeed a patent document, and its purpose was to pro[illegible]
details regarding the process of constructing the knowledge graph. However, since the construction process is not the main f[illegible]
we do not believe it is necessary to publish this document alongside the paper.

#Q10: Related to point 8, in the COI section, you could be more specific about the involvement of Smartquerier Gene Techno[illegible]
Co (e.g. if the full KG is proprietary to this company).
# Response: Thanks for your suggestion. I have revised the COI section to clarify the involvement of Smartquerier Gene Tec[illegible]
(Shanghai) Co., including the proprietary nature of the SmartQuerier Oncology Knowledge Graph (SOKG) used in the study ([illegible]
567).
