# Supplementary material for: Knowledge graph–based thought: a knowledge graph–enhanced LLM framework for pan-cancer question answering
Source: Gigascience. 2025 Jan 6;14:giae082. doi: 10.1093/gigascience/giae082 (PMC11702363; doi:10.1093/gigascience/giae082)
Supplement: giae082_Reviewer_1_Report_Original_Submission — Linhao Luo -- 7/2/2024 [file giae082_reviewer_1_report_original_submission.pdf]

Reviewer Report

Title: Knowledge Graph-based Thought: a knowledge graph enhanced LLMs framework for pan-cancer question answering

Version: Original Submission **Date:** 7/2/2024

Reviewer name: Linhao Luo

Reviewer Comments to Author:

Sumary:
This paper proposes a novel framework called KGT that integrates Large Language Models (LLMs) with Knowledge Graphs (KGs) for pan-cancer question answering. The KGT framework can effectively retrieve knowledge from KGs and improve the accuracy of LLMs for question answering. Moreover, it can provide interpretable and faithful explanations with the help of structured KGs.
Comments:
1. This paper construct a new dataset denoted as PcQA form a customized KG called SOKG for the evaluation of pan-cancer question answering. This is a great contribution to the community. However, it is unclear how to constuct such a dataset. More details about the construnction process and statistics of the final datasets should be disscussed in the paper. For example, how to generate the natural language questions and answers? How to link the question with relatived KG information (i.e., entity and relation)? How many questions can be answered by the KGs (i.e., answer converage rate). How many questions have been generated? What is the ratio of each quetion types defined in Table 2?
2. In Table2, the author define 4 reasoning types. How about other reasoning types such union and negation? Can we incorpate these tpes into the datasets?
3. The propsed method is novel and interesting. However some details are unclear. In the candidate path search, do we want to search reasoning paths or relational chains? The definition of these two paths are also unclear. Please give clear definition of them in prelimary. If is the reasoning paths, do we only keep the type information during the BFS?
4. I do not understand why we need to generatea cypher query to retrieve subgraph then construct relation paths from KG. We can directly retrieved relational paths from KGs by BFS. What are the benefits and motivations of using this two-stage pipeline?
5. What are the meanings of the X and âˆš in the figure. How to get them?
6. In experiments, other advanced KGQA methods can be compared, e.g., RoG [1] and ToG [2].
7. The analysis of used token, time, and cost should be disscussed in the paper.
8. Can we apply the proposed metod to other KGs (i.e., SynLethKG, and SDKG) or KGQA tasks (MetaQA, and FACTKG) to show the generability.
[1] LUO, L., Li, Y. F., Haf, R., & Pan, S. Reasoning on Graphs: Faithful and Interpretable Large Language Model Reasoning. In The Twelfth International Conference on Learning Representations.
[2] Sun, J., Xu, C., Tang, L., Wang, S., Lin, C., Gong, Y., ... & Guo, J. (2023). Think-on-graph: Deep and responsible reasoning of large language model with knowledge graph. arXiv preprint arXiv:2307.07697.

Methods

Are the methods appropriate to the aims of the study, are they well described, and are necessary controls included? Choose an item.

Conclusions

Are the conclusions adequately supported by the data shown? Choose an item.

Reporting Standards

Does the manuscript adhere to the journal's guidelines on minimum standards of reporting? Choose an item.

Choose an item.

Statistics

Are you able to assess all statistics in the manuscript, including the appropriateness of statistical tests used? Choose an item.

Quality of Written English

Please indicate the quality of language in the manuscript: Choose an item.

Declaration of Competing Interests

Please complete a declaration of competing interests, considering the following questions:

- Have you in the past five years received reimbursements, fees, funding, or salary from an organisation that may in any way gain or lose financially from the publication of this manuscript, either now or in the future?
- Do you hold any stocks or shares in an organisation that may in any way gain or lose financially from the publication of this manuscript, either now or in the future?
- Do you hold or are you currently applying for any patents relating to the content of the manuscript?
- Have you received reimbursements, fees, funding, or salary from an organization that holds or has applied for patents relating to the content of the manuscript?
- Do you have any other financial competing interests?
- Do you have any non-financial competing interests in relation to this paper?

If you can answer no to all of the above, write 'I declare that I have no competing interests' below. If your reply is yes to any, please give details below.

I declare that I have no competing interests

I agree to the open peer review policy of the journal. I understand that my name will be included on my report to the authors and, if the manuscript is accepted for publication, my named report including any attachments I upload will be posted on the website along with the authors' responses. I agree for my report to be made available under an Open Access Creative Commons CC-BY license (http://creativecommons.org/licenses/by/4.0/). I understand that any comments which I do not wish to be included in my named report can be included as confidential comments to the editors, which will not be published.

Choose an item.

To further support our reviewers, we have joined with Publons, where you can gain additional credit to further highlight your hard work (see: https://publons.com/journal/530/gigascience). On publication of this paper, your review will be automatically added to Publons, you can then choose whether or not to claim your Publons credit. I understand this statement.

Yes Choose an item.
